# Supplementary material for: Cooperative Cu/azodiformate system-catalyzed allylic C–H amination of unactivated internal alkenes directed by aminoquinoline
Source: Nat Commun. 2024 Feb 19;15:1483. doi: 10.1038/s41467-024-45875-y (PMC10876528; doi:10.1038/s41467-024-45875-y)
Supplement: Supplementary file 1 — Supplementary Information [file 41467_2024_45875_MOESM1_ESM.pdf]

Supplementary Information

Cooperative Cu/Azodiformate-Catalyzed Allylic C−H Amination of Unactivated Internal Alkenes Directed by Aminoquinoline

Le Wang, Cheng-Long Wang, Zi-Hao Li, Peng-Fei Lian, Jun-Chen Kang, Jia Zhou, Yu Hao, Ru-Xin Liu, He-Yuan Bai and Shu-Yu Zhang*

Shanghai Key Laboratory for Molecular Engineering of Chiral Drugs/School of Chemistry and Chemical Engineering, Shanghai Jiao Tong University,

Shanghai 200240, P. R. China

E-mail: zhangsy16@sjtu.edu.cn

1. General information S2
2. General procedure for synthesis of 3-alkenamide substrates S2
3. Investigation the substrate scopes of azo compounds S15
4. General procedure for Cu-catalyzed allylic C−H amination reactions S15
5. Synthetic utilities S29
6. Primary kinetic isotope effects S32
7. General procedure for KIE experiments S32
8. Crystal structure information for compound **3a** S38
9. Computational details S39
10. NMR spectra S41
11. References S87

1. General information

Reagents: All commercial materials were used as received from Energy Chemical or Adamas-beta, Alfa Aesar, TCI and Acros unless otherwise noted.

Chromatography: Thin layer chromatography (TLC) was carried out on silica gel 60 F254 pre-coated glass plates. Visualization was detected by irradiation with UV light (254 nm), or by treatment with a solution of phosphomolybdic acid in ethanol followed by heating. Flash chromatography was carried out on 200 – 300 mesh silica gel, eluting with a mixture of petroleum ether (b.p. 60 – 90 °C) and ethyl acetate.

NMR Spectroscope: ^{1}H NMR and ^{13}C NMR spectra were recorded on a Bruker AVANCE III HD 400 or 500 spectrometer, operating at 400 (or 500) MHz and 100 (or 125) MHz respectively. Chemical shifts (δ) were given in parts per million (ppm), and referenced relative to residual solvent $CHCl_3$ (7.26 ppm) in $CDCl_3$, or tetramethylsilane (0.00 ppm) as an internal standard for ^{1}H NMR spectra and deuterated solvent $CDCl_3$ (77.0 ppm) for ^{13}C NMR spectra. Coupling constants (*J*) were reported in hertz (Hz). The following abbreviations are used to indicate the multiplicity of the signals: s = singlet, d = doublet, t = triplet, q = quartet, m = multiplet, and associated combinations, e.g. dd = doublet of doublets.

Mass Spectrometry: High-resolution mass spectra (HRMS) were obtained on a Bruker Buker impact II using the electrospray ionization (ESI) technique.

2. Synthesis of 3-Alkenamide Substrates

2.1 Most of the alkenamide substrates are known compounds and were synthesized according to the literature reports[1-3].

2.2 General procedure for the preparation alkenamide substrates.

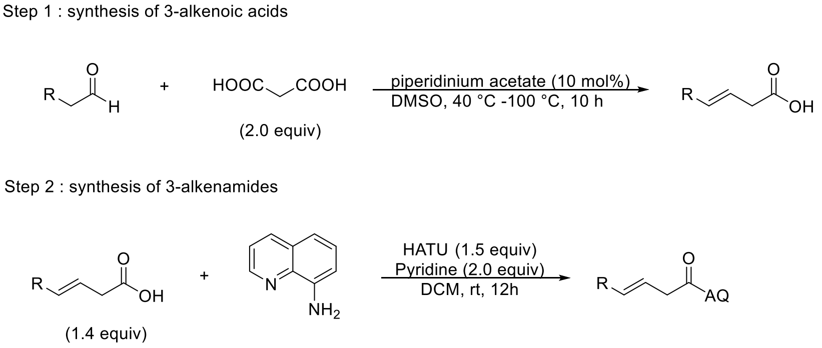

Step 1: The (*E*)-3-alkenoic acids were synthesized according to the reported procedures. To a stirred solution of aldehyde in DMSO (1M), malonic acid (2.0 equiv), acetic acid (6 μL) and piperidine (10 μL) were added in one portion at room temperature. The mixture was heated at 40 ºC for 4 h and then heat to 100 ºC for 12 h. After being cooled to room temperature, the reaction mixture was poured into brine, and extracted with ethyl acetate. The combined organic layer was dried over

Na_2SO_4, filtered and concentrated *in vacuo*. The resulting residue was purified by silica gel flash chromatography to give the desired product.

Step 2: A mixture of 8-Aminoquinoline (1.0 equiv.), corresponding carboxylic acid (1.2 equiv.), HATU (1.5 equiv.), pyridine (2.0 equiv.) in anhydrous DCM (0.2 M) was stirred at room temperature overnight. Water was added and the mixture was extracted with DCM. The combined organic layer was washed with water and brine, dried over anhydrous Na_2SO_4, and concentrated *in vacuo*. The resulting residue was purified by silica gel flash chromatography to give the desired product.

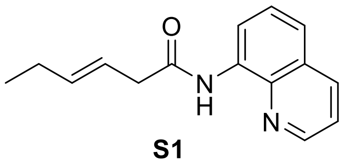

S1

(*E*)-N-(quinolin-8-yl)hex-3-enamide (S1) : Pale yellow solid, 75% yield. **^{1}H NMR** (400 MHz, Chloroform-d) δ 10.1 (s, 1H), 9.0 – 8.6 (m, 2H), 8.4 – 7.9 (m, 1H), 7.6 – 7.4 (m, 3H), 6.0 – 5.3 (m, 2H), 3.3 (d, J = 7.0 Hz, 2H), 2.3 – 2.0 (m, 2H), 1.2 – 0.9 (m, 3H). **^{13}C NMR** (101 MHz, Chloroform-d) δ 170.0, 148.1, 138.7, 138.5, 136.2, 134.5, 127.9, 127.3, 121.5, 121.4, 121.4, 116.2, 42.1, 25.8, 13.5. **HRMS:** calculated for $C_{15}H_{16}N_2ONa^+$ [M+Na$^+$]: 263.1155; found: 263.1154.

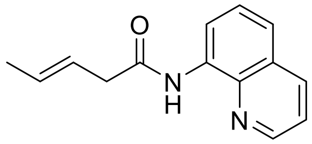

S2

(*E*)-N-(quinolin-8-yl)pent-3-enamide (S2) : Pale yellow solid, 76% yield. **^{1}H NMR** (400 MHz, Chloroform-d) δ 10.0 (s, 1H), 9.0 – 8.5 (m, 2H), 8.1 (dt, J = 8.3, 1.4 Hz, 1H), 7.7 – 7.4 (m, 3H), 6.1 – 5.4 (m, 2H), 3.3 (dd, J = 6.2, 1.4 Hz, 2H), 2.0 – 1.3 (m, 3H). **^{13}C NMR** (126 MHz, Chloroform-d) δ 170.1, 148.2, 138.5, 136.3, 134.4, 131.3, 127.9, 127.4, 123.5, 121.5, 121.5, 116.3, 42.1, 18.1. **HRMS:** calculated for $C_{14}H_{14}N_2ONa^+$ [M+Na$^+$]: 249.0998; found: 249.0994.

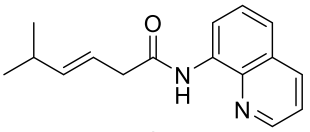

S3

(*E*)-5-methyl-N-(quinolin-8-yl)hex-3-enamide (S3) : Pale yellow solid, 82% yield. **^{1}H NMR** (400 MHz, Chloroform-d) δ 10.1 (s, 1H), 9.2 – 8.5 (m, 2H), 8.1 (dt, J = 8.2, 1.9 Hz, 1H), 7.7 – 7.3 (m,

3H), 6.1 – 5.3 (m, 2H), 3.6 – 3.0 (m, 2H), 2.6 – 2.2 (m, 1H), 1.4 – 0.7 (m, 6H). **^{13}C NMR** (101 MHz, Chloroform-d) δ 170.1, 148.0, 144.5, 138.5, 136.2, 134.4, 127.9, 127.3, 121.5, 121.4, 119.4, 116.2, 42.1, 31.4, 22.3. **HRMS:** calculated for $C_{16}H_{18}N_2ONa^+$ [M+Na$^+$]: 277.1311; found: 277.1314.

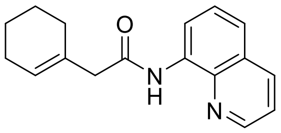

S4

2-(cyclohex-1-en-1-yl)-N-(quinolin-8-yl)acetamide (S4) : White solid, 86% yield. **^{1}H NMR** (400 MHz, Chloroform-d) δ 10.2 (s, 1H), 8.9 – 8.5 (m, 2H), 8.1 (dd, J = 8.3, 1.7 Hz, 1H), 7.7 – 7.3 (m, 3H), 5.9 (d, J = 3.9 Hz, 1H), 3.2 (s, 2H), 2.5 – 1.8 (m, 4H), 1.8 – 1.2 (m, 4H). **^{13}C NMR** (101 MHz, Chloroform-d) δ 170.0, 148.2, 138.7, 136.3, 134.5, 132.5, 128.0, 127.9, 127.4, 121.6, 121.4, 116.2, 47.9, 28.7, 25.6, 22.8, 22.0. **HRMS:** calculated for $C_{17}H_{18}N_2ONa^+$ [M+Na$^+$]: 289.1311; found: 289.1310.

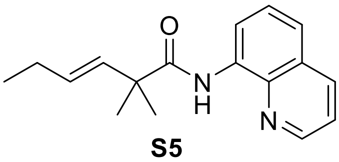

S5

(*E*)-2,2-dimethyl-N-(quinolin-8-yl)hex-3-enamide (S5) : Pale yellow liquid, 73% yield. **^{1}H NMR** (400 MHz, Chloroform-d) δ 10.4 (s, 1H), 9.1 – 8.3 (m, 2H), 8.1 (dd, J = 8.3, 1.6 Hz, 1H), 7.7 – 7.3 (m, 3H), 6.2 – 5.3 (m, 2H), 2.2 (tt, J = 8.0, 6.6 Hz, 2H), 1.5 (s, 6H), 1.2 (t, J = 7.4 Hz, 3H). **^{13}C NMR** (101 MHz, Chloroform-d) δ 175.8, 148.1, 138.9, 136.2, 134.8, 133.8, 133.1, 127.9, 127.4, 121.5, 121.2, 116.0, 46.0, 26.0, 25.5, 13.8. **HRMS:** calculated for $C_{17}H_{20}N_2ONa^+$ [M+Na$^+$]: 291.1468; found: 291.1463.

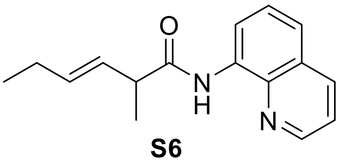

S6

(*E*)-2-methyl-N-(quinolin-8-yl)hex-3-enamide (S6) : Pale yellow liquid, 75% yield. **^{1}H NMR** (500 MHz, Chloroform-d) δ 10.1 (s, 1H), 9.1 – 8.5 (m, 2H), 8.1 (dd, J = 8.3, 1.7 Hz, 1H), 7.6 – 7.4 (m, 3H), 6.0 – 5.8 (m, 1H), 5.8 – 5.5 (m, 1H), 3.5 – 3.0 (m, 1H), 2.5 – 1.8 (m, 2H), 1.4 (d, J = 7.0 Hz, 3H), 1.1 (t, J = 7.5 Hz, 3H). **^{13}C NMR** (126 MHz, Chloroform-d) δ 173.3, 148.1, 138.7, 136.3,

135.8, 134.7, 128.7, 128.0, 127.4, 121.5, 121.3, 116.2, 46.0, 25.8, 17.3, 13.6. **HRMS:** calculated for $C_{16}H_{18}N_2ONa^+$ [M+Na$^+$]: 277.1311; found: 277.1306.

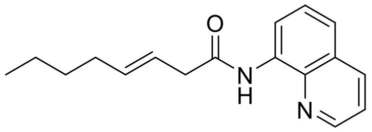

S7

(*E*)-N-(quinolin-8-yl)oct-3-enamide (S7) : Pale yellow liquid, 86% yield. **^{1}H NMR** (400 MHz, Chloroform-d) δ 10.1 (s, 1H), 8.9 – 8.6 (m, 2H), 8.1 (dd, J = 8.3, 1.7 Hz, 1H), 7.7 – 7.3 (m, 3H), 5.9 – 5.6 (m, 2H), 3.3 (d, J = 6.8 Hz, 2H), 2.2 (q, J = 7.0 Hz, 2H), 1.6 – 1.3 (m, 4H), 0.9 (t, J = 7.2 Hz, 3H). **^{13}C NMR** (126 MHz, Chloroform-d) δ 170.1, 148.1, 138.6, 137.3, 136.3, 134.5, 127.9, 127.4, 122.2, 121.5, 121.5, 116.3, 42.2, 32.4, 31.5, 22.3, 14.0. **HRMS:** calculated for $C_{17}H_{20}N_2ONa^+$ [M+Na$^+$]: 291.1468; found: 291.1464.

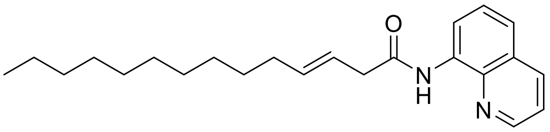

S8

(*E*)-N-(quinolin-8-yl)oct-3-enamide (S8) : Pale yellow liquid, 68% yield. **^{1}H NMR** (400 MHz, Chloroform-d) δ 10.1 (s, 1H), 8.9 – 8.6 (m, 2H), 8.1 (d, J = 8.2 Hz, 1H), 7.8 – 7.3 (m, 3H), 5.9 – 5.7 (m, 2H), 3.3 (d, J = 6.8 Hz, 2H), 2.2 (q, J = 7.1 Hz, 2H), 1.5 (q, J = 7.3 Hz, 2H), 1.3 – 1.2 (m, 14H), 0.9 (t, J = 6.8 Hz, 3H). **^{13}C NMR** (101 MHz, Chloroform-d) δ 170.2, 148.1, 138.6, 137.4, 136.3, 134.5, 128.0, 127.4, 122.2, 121.5, 121.5, 116.4, 42.2, 32.8, 31.9, 29.7, 29.6, 29.6, 29.4, 29.3, 29.3, 22.7, 14.1. **HRMS:** calculated for $C_{23}H_{32}N_2ONa^+$ [M+Na$^+$]: 374.2407; found: 374.2411.

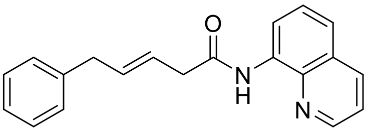

S9

(*E*)-5-phenyl-N-(quinolin-8-yl)pent-3-enamide (S9) : Pale yellow liquid, 72% yield. **^{1}H NMR** (500 MHz, Chloroform-d) δ 10.0 (s, 1H), 8.8 – 8.5 (m, 2H), 8.1 (dd, J = 8.3, 1.7 Hz, 1H), 7.6 – 7.4 (m, 3H), 7.3 – 7.3 (m, 4H), 7.2 – 7.2 (m, 1H), 6.0 (dtt, J = 14.6, 6.6, 1.3 Hz, 1H), 5.9 – 5.7 (m, 1H), 3.5 (dd, J = 6.6, 1.4 Hz, 2H), 3.3 (dd, J = 7.1, 1.3 Hz, 2H). **^{13}C NMR** (126 MHz, Chloroform-d) δ 169.7, 148.1, 139.9, 138.5, 136.3, 135.3, 134.4, 128.7, 128.5, 127.9, 127.3, 126.1, 123.7, 121.5,

116.4, 42.0, 39.1. **HRMS:** calculated for $C_{20}H_{18}N_2ONa^+$ [M+Na$^+$]: 325.1311; found: 325.1307.

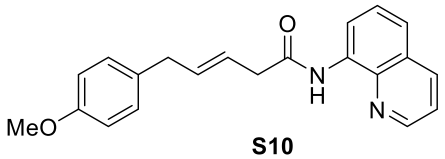

S10

(*E*)-5-(4-methoxyphenyl)-N-(quinolin-8-yl)pent-3-enamide (S10) : Yellow liquid, 85% yield. **^{1}H NMR** (400 MHz, Chloroform-d) δ 10.0 (s, 1H), 8.8 (dt, J = 6.3, 1.6 Hz, 2H), 8.1 (dd, J = 8.3, 1.7 Hz, 1H), 7.8 – 7.3 (m, 3H), 7.2 – 7.2 (m, 2H), 6.9 – 6.4 (m, 2H), 6.1 – 5.9 (m, 1H), 5.9 – 5.7 (m, 1H), 3.8 (s, 3H), 3.4 (d, J = 6.5 Hz, 2H), 3.3 – 3.1 (m, 2H).

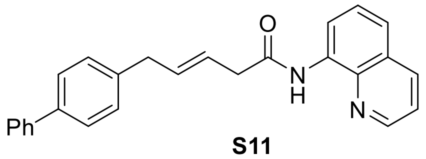

S11

(*E*)-5-([1,1'-biphenyl]-4-yl)-N-(quinolin-8-yl)pent-3-enamide (S11) : Yellow liquid, 82% yield. **^{1}H NMR** (400 MHz, Chloroform-d) δ 10.1 (s, 1H), 8.8 (dd, J = 12.9, 5.8 Hz, 2H), 8.1 (d, J = 8.3 Hz, 1H), 7.7 – 7.3 (m, 12H), 6.3 – 5.6 (m, 2H), 3.5 (d, J = 6.5 Hz, 2H), 3.3 (d, J = 7.0 Hz, 2H). **^{13}C NMR** (101 MHz, Chloroform-d) δ 169.7, 148.2, 141.0, 139.2, 139.0, 138.5, 136.3, 135.2, 134.4, 129.1, 128.7, 127.9, 127.4, 127.2, 127.1, 127.0, 123.9, 121.6, 116.4, 42.0, 38.7. **HRMS:** calculated for $C_{26}H_{22}N_2ONa^+$ [M+Na$^+$]: 401.1624; found: 401.1628.

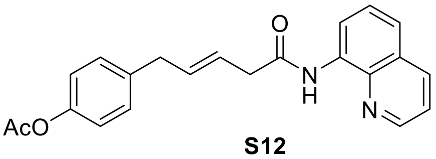

S12

(*E*)-4-(5-oxo-5-(quinolin-8-ylamino)pent-2-en-1-yl)phenyl acetate (S12) : Yellow liquid, 82% yield. **^{1}H NMR** (500 MHz, Chloroform-d) δ 10.0 (s, 1H), 8.9 – 8.7 (m, 2H), 8.2 (dd, J = 8.3, 1.7 Hz, 1H), 7.9 – 7.8 (m, 2H), 7.6 – 7.5 (m, 2H), 7.5 (dd, J = 8.3, 4.2 Hz, 1H), 7.4 – 7.4 (m, 2H), 6.1 – 5.7 (m, 2H), 3.6 (d, J = 6.5 Hz, 2H), 3.3 (dd, J = 7.0, 1.2 Hz, 2H), 2.6 (s, 3H). **^{13}C NMR** (126 MHz, Chloroform-d) δ 197.9, 169.5, 148.2, 145.6, 138.4, 136.4, 135.3, 134.3, 134.0, 128.9, 128.6, 127.9, 127.4, 124.7, 121.7, 121.6, 116.4, 41.9, 39.0, 26.6. **HRMS:** calculated for $C_{22}H_{20}N_2O_3Na^+$ [M+Na$^+$]: 383.1366; found: 383.1362.

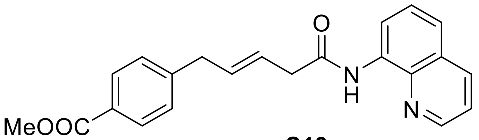

S13

Methyl (*E*)-4-(5-oxo-5-(quinolin-8-ylamino)pent-2-en-1-yl)benzoate (S13) : Yellow liquid, 75% yield. **^{1}H NMR** (400 MHz, Chloroform-d) δ 10.0 (s, 1H), 8.8 – 8.7 (m, 2H), 8.2 (dt, J = 8.3, 1.8 Hz, 1H), 8.0 – 7.9 (m, 2H), 7.5 – 7.5 (m, 2H), 7.5 (dd, J = 8.2, 4.1 Hz, 1H), 7.4 (d, J = 8.1 Hz, 2H), 6.0 – 5.8 (m, 2H), 3.9 (s, 3H), 3.6 (d, J = 6.4 Hz, 2H), 3.5 – 3.2 (m, 2H). **^{13}C NMR** (101 MHz, Chloroform-d) δ 169.5, 167.1, 148.2, 145.4, 138.5, 136.4, 134.2, 129.8, 128.8, 128.2, 128.0, 127.4, 124.6, 121.6, 116.4, 52.0, 42.0, 39.0. **HRMS:** calculated for $C_{22}H_{20}N_2O_3Na^+$ [M+Na$^+$]: 383.1366; found: 383.1365.

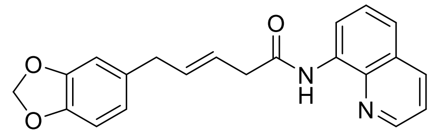

S14

(*E*)-5-(benzo[d][1,3]dioxol-5-yl)-N-(quinolin-8-yl)pent-3-enamide (S14) : Pale yellow solid, 84% yield. **^{1}H NMR** (400 MHz, Chloroform-d) δ 10.0 (s, 1H), 9.1 – 8.7 (m, 2H), 8.2 (dd, J = 8.2, 1.7 Hz, 1H), 7.7 – 7.4 (m, 3H), 6.8 (d, J = 38.7 Hz, 2H), 6.0 – 5.9 (m, 2H), 3.4 (d, J = 6.4 Hz, 2H), 3.3 (d, J = 7.3 Hz, 2H). **^{13}C NMR** (101 MHz, Chloroform-d) δ 169.7, 148.3, 147.7, 145.9, 138.5, 136.3, 135.6, 134.4, 133.7, 128.0, 127.4, 123.6, 121.6, 121.6, 121.5, 116.4, 109.3, 108.2, 100.8, 42.0, 38.8. **HRMS:** calculated for $C_{21}H_{18}N_2O_3Na^+$ [M+Na$^+$]: 369.1210; found: 369.1203.

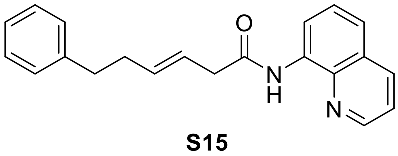

S15

(*E*)-6-phenyl-N-(quinolin-8-yl)hex-3-enamide (S15) : Yellow liquid, 81% yield. **^{1}H NMR** (400 MHz, Chloroform-d) δ 10.0 (s, 1H), 9.0 – 8.6 (m, 2H), 8.1 (dd, *J* = 8.3, 1.7 Hz, 1H), 7.5 – 7.4 (m, 2H), 7.4 (dd, *J* = 8.2, 4.2 Hz, 1H), 7.3 – 7.1 (m, 5H), 6.1 – 5.5 (m, 2H), 3.2 (d, *J* = 6.3 Hz, 2H), 2.8 (dd, *J* = 9.2, 6.6 Hz, 2H), 2.5 – 2.1 (m, 2H). **^{13}C NMR** (101 MHz, Chloroform-d) δ 148.2, 141.8, 138.6, 136.3, 136.1, 134.5, 128.5, 128.4, 128.0, 127.4, 125.9, 123.1, 121.6, 121.5, 116.4, 42.1, 35.8, 34.7. **HRMS:** calculated for $C_{21}H_{20}N_2ONa^+$ [M+Na$^+$]: 339.1468; found: 339.1465.

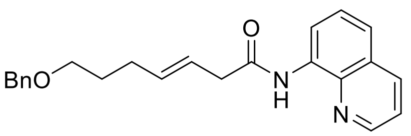

S16

(*E*)-7-(benzyloxy)-N-(quinolin-8-yl)hept-3-enamide (S16) : Pale yellow solid, 82% yield. **^{1}H NMR** (400 MHz, Chloroform-d) δ 10.0 (s, 1H), 9.0 – 8.5 (m, 2H), 8.1 (dd, J = 8.3, 1.7 Hz, 1H), 7.6 – 7.4 (m, 4H), 7.3 – 7.1 (m, 5H), 6.0 – 5.5 (m, 2H), 4.5 (d, J = 15.0 Hz, 2H), 3.5 (dt, J = 17.5, 6.5 Hz, 2H), 3.3 (dd, J = 36.0, 6.4 Hz, 2H), 2.5 – 2.1 (m, 2H), 2.0 – 1.6 (m, 2H). **^{13}C NMR** (126 MHz, Chloroform-d) δ 170.0, 148.2, 138.6, 136.4, 136.3, 134.5, 128.4, 127.7, 127.4, 122.9, 121.6, 116.3, 72.9, 69.7, 42.2, 29.4, 29.3. **HRMS:** calculated for $C_{23}H_{24}N_2O_2Na^+$ [M+Na$^+$]: 383.1730; found: 383.1723.

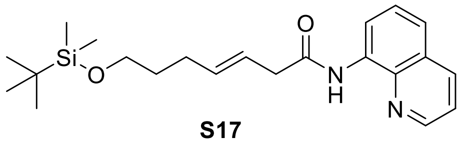

S17

(*E*)-6-((tert-butyldimethylsilyl)oxy)-N-(quinolin-8-yl)hex-3-enamide (S17) : Yellow liquid, 88% yield. **^{1}H NMR** (400 MHz, Chloroform-d) δ 9.9 (s, 1H), 9.0 – 8.6 (m, 2H), 8.1 (dd, *J* = 8.3, 1.7 Hz, 1H), 7.6 – 7.3 (m, 3H), 5.8 (td, *J* = 3.9, 1.9 Hz, 2H), 3.7 (t, *J* = 6.9 Hz, 2H), 3.2 – 3.0 (m, 2H), 2.5 – 2.1 (m, 2H), 0.8 (s, 9H), 0.0 (s, 6H).

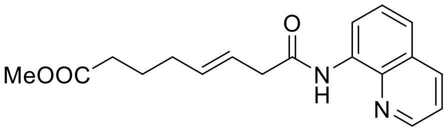

S18

Methyl (*E*)-8-oxo-8-(quinolin-8-ylamino)oct-5-enoate (S18) : Yellow liquid, 55% yield. **^{1}H NMR** (400 MHz, Chloroform-d) δ 10.0 (s, 1H), 8.8 (ddd, *J* = 9.0, 5.8, 1.7 Hz, 2H), 8.1 (dd, *J* = 8.3, 1.7 Hz, 1H), 7.6 – 7.3 (m, 3H), 5.9 – 5.5 (m, 2H), 3.7 (s, 3H), 3.3 (dd, *J* = 4.8, 1.7 Hz, 2H), 2.4 (t, *J* = 7.6 Hz, 2H), 2.3 – 2.1 (m, 2H), 1.9 – 1.7 (m, 2H). **^{13}C NMR** (101 MHz, Chloroform-d) δ 174.0, 169.8, 148.3, 138.5, 136.3, 135.7, 134.4, 127.9, 127.4, 123.5, 121.6, 121.5, 116.3, 51.5, 42.1, 33.5, 32.0, 24.5. **HRMS:** calculated for $C_{18}H_{20}N_2O_3Na^+$ [M+Na$^+$]: 335.1366; found: 335.1359.

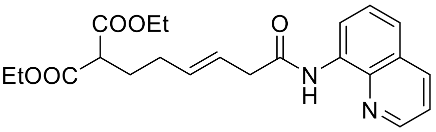

S19

Diethyl (*E*)-2-(6-oxo-6-(quinolin-8-ylamino)hex-3-en-1-yl)malonate (S19) : Yellow liquid, 67% yield. **^{1}H NMR** (400 MHz, Chloroform-d) δ 10.0 (s, 1H), 9.0 – 8.6 (m, 2H), 8.2 (dd, J = 8.3, 1.7 Hz, 1H), 7.7 – 7.3 (m, 3H), 6.0 – 5.3 (m, 2H), 4.4 – 3.8 (m, 4H), 3.4 (q, J = 7.8 Hz, 1H), 3.4 – 3.2 (m, 2H), 2.6 – 1.8 (m, 4H), 1.4 – 0.9 (m, 6H). **^{13}C NMR** (101 MHz, Chloroform-d) δ 169.7, 169.4, 148.4, 138.5, 136.3, 135.0, 134.4, 127.9, 127.4, 124.1, 123.2, 121.6, 116.3, 61.4, 51.4, 42.1, 36.8, 30.4, 28.3, 25.2, 14.1. **HRMS:** calculated for $C_{22}H_{26}N_2O_5Na^+$ [M+Na$^+$]: 421.1734; found: 421.1735.

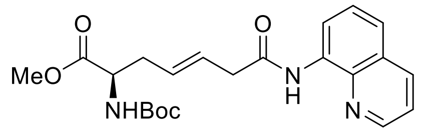

S20

Methyl (*R,E*)-2-((tert-butoxycarbonyl)amino)-7-oxo-7-(quinolin-8-ylamino)hept-4-enoate (S20) : Yellow liquid, 84% yield. **^{1}H NMR** (400 MHz, Chloroform-d) δ 9.9 (s, 1H), 9.1 – 8.5 (m, 2H), 8.2 (dt, J = 8.3, 1.8 Hz, 1H), 7.8 – 7.4 (m, 3H), 5.7 (dt, J = 15.0, 7.1 Hz, 2H), 4.7 – 4.3 (m, 1H), 3.8 (s, 3H), 3.4 – 3.1 (m, 2H), 2.8 (d, J = 0.9 Hz, 2H), 1.3 (s, 9H).

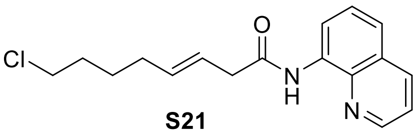

S21

(*E*)-8-chloro-N-(quinolin-8-yl)oct-3-enamide (S21) : Yellow liquid, 83% yield. **^{1}H NMR** (400 MHz, Chloroform-d) δ 10.0 (s, 1H), 9.1 – 8.5 (m, 2H), 8.1 (dd, J = 8.2, 1.7 Hz, 1H), 7.7 – 7.4 (m, 3H), 6.2 – 5.5 (m, 2H), 3.6 (t, J = 6.6 Hz, 2H), 3.3 – 3.1 (m, 2H), 2.2 (td, J = 7.8, 5.6 Hz, 2H), 1.9 (dt, J = 15.1, 6.7 Hz, 2H), 1.7 (tt, J = 10.0, 6.3 Hz, 2H). **^{13}C NMR** (126 MHz, Chloroform-d) δ 169.9, 148.2, 138.5, 136.3, 136.1, 134.4, 127.9, 127.4, 123.0, 121.6, 121.6, 116.4, 45.0, 42.1, 32.1, 31.9, 26.4. **HRMS:** calculated for $C_{17}H_{19}ClN_2ONa^+$ [M+Na$^+$]: 325.1078; found: 325.1076.

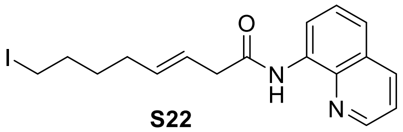

S22

(*E*)-8-iodo-N-(quinolin-8-yl)oct-3-enamide (S22) : Black solid, 87% yield. **^{1}H NMR** (500 MHz, Chloroform-d) δ 10.0 (s, 1H), 8.8 – 8.7 (m, 2H), 8.2 (dd, J = 8.3, 1.7 Hz, 1H), 7.5 – 7.4 (m, 3H), 5.9 – 5.7 (m, 2H), 3.3 (d, J = 5.8 Hz, 2H), 2.2 (td, J = 7.4, 5.4 Hz, 2H), 2.0 – 1.9 (m, 2H), 1.7 – 1.6 (m, 2H). **^{13}C NMR** (126 MHz, Chloroform-d) δ 170.0, 148.1, 138.3, 136.6, 136.0, 134.3, 128.0, 127.5, 123.1, 121.6, 121.6, 116.6, 42.1, 32.9, 31.6, 30.0, 7.0. **HRMS:** calculated for $C_{17}H_{19}IN_2ONa^+$

[M+Na$^+$]: 417.0434; found: 417.0426.

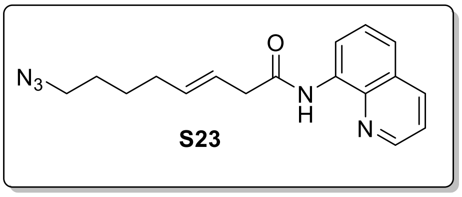

(*E*)-8-azido-N-(quinolin-8-yl)oct-3-enamide (S23) : Yellow solid, 54% yield. **^{1}H NMR** (500 MHz, Chloroform-d) δ 10.0 (s, 1H), 8.9 – 8.6 (m, 2H), 8.2 (dd, J = 8.3, 1.7 Hz, 1H), 7.6 – 7.4 (m, 3H), 5.9 – 5.7 (m, 2H), 3.4 – 3.2 (m, 4H), 2.2 (td, J = 7.3, 5.0 Hz, 2H), 1.8 – 1.5 (m, 4H).

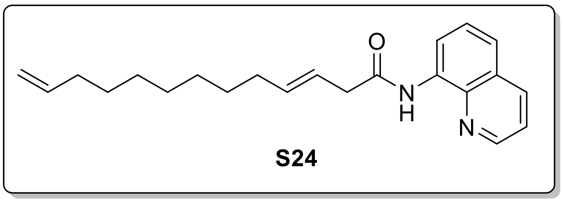

(*E*)-N-(quinolin-8-yl)trideca-3,12-dienamide (S24) : Yellow liquid, 83% yield. **^{1}H NMR** (400 MHz, Chloroform-d) δ 10.1 (s, 1H), 8.8 (dt, J = 5.2, 1.6 Hz, 2H), 8.1 (dd, J = 8.2, 1.7 Hz, 1H), 7.6 – 7.3 (m, 3H), 5.9 – 5.5 (m, 3H), 5.1 – 4.5 (m, 2H), 3.6 – 2.9 (m, 2H), 2.2 (q, J = 6.8 Hz, 2H), 2.1 – 1.9 (m, 2H), 1.6 – 1.4 (m, 2H), 1.4 – 1.2 (m, 8H). **^{13}C NMR** (126 MHz, Chloroform-d) δ 170.2, 148.1, 139.2, 138.6, 137.3, 136.3, 134.5, 128.0, 127.4, 122.2, 121.6, 121.5, 116.4, 114.2, 42.2, 33.8, 32.8, 29.4, 29.3, 29.2, 29.1, 28.9. **HRMS:** calculated for $C_{22}H_{28}N_2ONa^+$ [M+Na$^+$]: 359.2094; found: 359.2090.

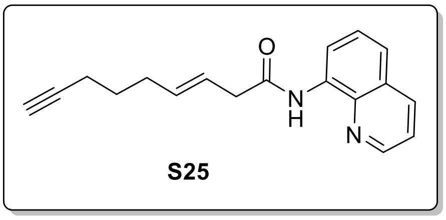

((*E*)-N-(quinolin-8-yl)non-3-en-8-ynamide (S25) : Yellow liquid, 67% yield. **^{1}H NMR** (400 MHz, Chloroform-d) δ 10.0 (s, 1H), 9.1 – 8.3 (m, 2H), 8.1 (dd, J = 8.3, 1.7 Hz, 1H), 7.7 – 7.3 (m, 3H), 6.0 – 5.4 (m, 2H), 3.6 – 3.0 (m, 2H), 2.4 – 2.1 (m, 4H), 2.1 – 1.9 (m, 1H), 1.8 – 1.6 (m, 2H). **^{13}C NMR** (126 MHz, Chloroform-d) δ 169.8, 148.2, 138.5, 136.3, 135.9, 134.4, 127.9, 127.4, 123.4, 121.6, 121.6, 116.3, 84.4, 68.6, 42.1, 31.6, 28.0, 17.9. **HRMS:** calculated for $C_{18}H_{18}N_2ONa^+$ [M+Na$^+$]: 301.1311; found: 301.1305.

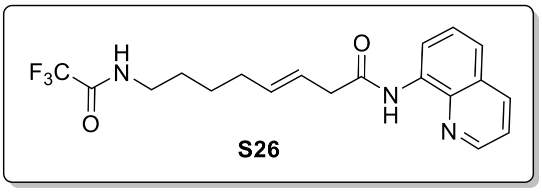

(*E*)-N-(quinolin-8-yl)-8-(2,2,2-trifluoroacetamido)oct-3-enamide (S26) : White solid, 84% yield. **^{1}H NMR** (400 MHz, Chloroform-d) δ 9.9 (d, J = 81.2 Hz, 1H), 8.8 (tdd, J = 11.8, 5.7, 1.8 Hz, 2H), 8.2 (dd, J = 8.3, 1.9 Hz, 1H), 7.7 – 7.4 (m, 3H), 6.7 (s, 1H), 5.9 – 5.6 (m, 2H), 3.4 (p, J = 6.9 Hz, 2H), 3.3 (d, J = 4.9 Hz, 2H), 2.2 (q, J = 6.6, 6.1 Hz, 2H), 1.7 (p, J = 7.2 Hz, 2H), 1.6 – 1.5 (m, 2H). **^{13}C NMR** (101 MHz, Chloroform-d) δ 170.0, 148.2, 148.2, 138.5, 136.4, 135.8, 134.3, 128.0, 127.4, 123.2, 121.7, 116.4, 41.9, 39.8, 32.0, 28.3, 26.0. **HRMS:** calculated for $C_{19}H_{20}F_3N_3O_2Na^+$ [M+Na$^+$]: 402.1400; found: 402.1392.

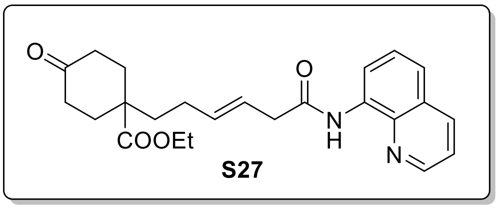

Ethyl (*E*)-4-oxo-1-(6-oxo-6-(quinolin-8-ylamino)hex-3-en-1-yl)cyclohexane-1-carboxylate (S27) : White solid, 84% yield. **^{1}H NMR** (400 MHz, Chloroform-d) δ 10.0 (s, 1H), 8.8 (ddp, J = 7.0, 4.6, 2.3 Hz, 2H), 8.2 – 8.1 (m, 1H), 7.6 – 7.4 (m, 3H), 5.8 (dd, J = 5.4, 3.2 Hz, 2H), 4.2 (dtt, J = 9.3, 4.5, 2.3 Hz, 2H), 3.4 – 3.2 (m, 2H), 2.6 – 2.3 (m, 6H), 2.2 – 2.1 (m, 2H), 1.9 – 1.7 (m, 2H), 1.4 – 1.1 (m, 5H). **^{13}C NMR** (101 MHz, Chloroform-d) δ 211.2, 175.1, 169.7, 148.1, 138.5, 136.4, 135.4, 134.3, 127.9, 127.4, 127.4, 123.2, 121.6, 116.4, 60.9, 60.4, 46.0, 41.9, 39.3, 38.4, 33.7, 27.8, 14.4, 14.2. **HRMS:** calculated for $C_{24}H_{28}N_2O_4Na^+$ [M+Na$^+$]: 431.1941; found: 431.1948.

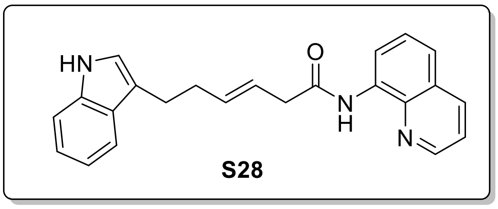

(*E*)-6-(1H-indol-3-yl)-N-(quinolin-8-yl)hex-3-enamide (S28) : White solid, 84% yield. **^{1}H NMR** (400 MHz, Chloroform-d) δ 10.1 (s, 1H), 8.8 – 8.5 (m, 2H), 8.3 – 7.9 (m, 2H), 7.7 (d, J = 7.9 Hz, 1H), 7.6 – 7.4 (m, 3H), 7.4 (d, J = 8.1 Hz, 1H), 7.2 – 7.0 (m, 3H), 6.1 (dt, J = 14.9, 6.4 Hz, 1H), 5.9 (dt, J = 14.9, 7.1 Hz, 1H), 3.6 (d, J = 6.4 Hz, 2H), 3.3 (d, J = 7.1 Hz, 2H), 2.3 – 2.0 (m, 2H). **^{13}C NMR** (101 MHz, Chloroform-d) δ 170.0, 148.2, 138.6, 136.4, 136.3, 135.3, 134.5, 128.0, 127.4, 123.0, 122.0, 121.9, 121.6, 119.3, 119.1, 116.5, 114.4, 111.1, 42.1, 32.6, 28.6. **HRMS:** calculated for $C_{23}H_{21}N_3ONa^+$ [M+Na$^+$]: 378.1577; found: 378.1573.

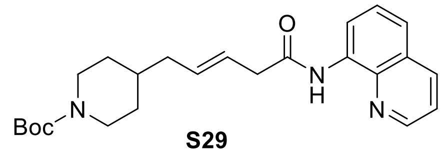

S29

Tert-butyl (*E*)-4-(5-oxo-5-(quinolin-8-ylamino)pent-2-en-1-yl)piperidine-1-carboxylate (S29) : Pale yellow solid, 81% yield. **^{1}H NMR** (400 MHz, Chloroform-d) δ 10.0 (s, 1H), 8.8 (dt, J = 5.7, 1.7 Hz, 2H), 8.2 (dd, J = 8.3, 1.7 Hz, 1H), 7.6 – 7.4 (m, 3H), 5.9 – 5.7 (m, 2H), 4.1 (s, 2H), 3.3 (d, J = 5.3 Hz, 2H), 2.7 (t, J = 12.6 Hz, 2H), 2.1 (t, J = 6.1 Hz, 2H), 1.8 (s, 2H), 1.6 (dtd, J = 11.3, 8.0, 4.2 Hz, 1H), 1.2 (qd, J = 12.3, 4.4 Hz, 2H). **^{13}C NMR** (101 MHz, Chloroform-d) δ 169.9, 154.9, 148.2, 138.5, 136.4, 134.5, 134.4, 128.0, 127.4, 124.0, 121.6, 121.6, 116.4, 42.2, 39.7, 36.1, 32.0, 28.5. **HRMS:** calculated for $C_{24}H_{31}N_3O_3Na^+$ [M+Na$^+$]: 432.2258; found: 432.2250.

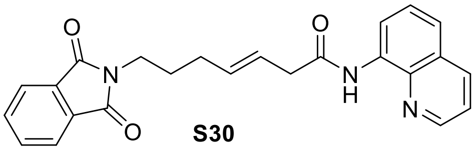

S30

(*E*)-7-(1,3-dioxoisoindolin-2-yl)-N-(quinolin-8-yl)hept-3-enamide (S30) : White solid, 78% yield. **^{1}H NMR** (400 MHz, Chloroform-d) δ 10.0 (s, 1H), 9.0 – 8.5 (m, 2H), 8.1 (dq, *J* = 8.4, 1.7 Hz, 1H), 7.8 – 7.7 (m, 2H), 7.7 (ddd, *J* = 8.7, 5.5, 3.4 Hz, 3H), 7.6 – 7.4 (m, 2H), 5.8 (q, *J* = 5.6 Hz, 2H), 3.8 – 3.6 (m, 2H), 3.3 (d, *J* = 5.5 Hz, 2H), 2.3 – 1.9 (m, 2H), 1.9 – 1.7 (m, 2H). **^{13}C NMR** (126 MHz, Chloroform-d) δ 176.5, 170.1, 168.5, 168.4, 148.2, 138.5, 136.4, 136.2, 134.5, 134.4, 133.8, 132.1, 132.1, 127.9, 127.4, 123.1, 122.9, 121.7, 121.6, 121.5, 116.4, 42.1, 37.8, 32.2, 28.1. **HRMS:** calculated for $C_{24}H_{21}N_3O_3Na^+$ [M+Na$^+$]: 422.1475; found: 422.1476.

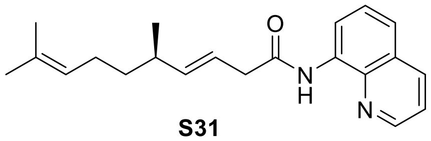

S31

(*E*)-5,9-dimethyl-N-(quinolin-8-yl)deca-3,8-dienamide (S31) : White solid, 67% yield. **^{1}H NMR** (400 MHz, Chloroform-d) δ 10.1 (s, 1H), 8.9 – 8.6 (m, 2H), 8.1 (dd, J = 8.3, 1.7 Hz, 1H), 7.6 – 7.3 (m, 3H), 5.7 (td, J = 3.9, 1.8 Hz, 2H), 5.1 (tq, J = 7.0, 1.4 Hz, 1H), 3.4 – 3.1 (m, 2H), 2.3 (p, J = 6.7 Hz, 1H), 2.1 (q, J = 7.6 Hz, 2H), 1.7 (d, J = 1.5 Hz, 3H), 1.6 (d, J = 1.4 Hz, 3H), 1.5 – 1.3 (m, 2H), 1.1 (d, J = 6.8 Hz, 3H).

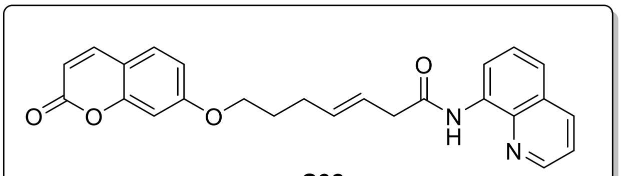

(*E*)-7-((2-oxo-2H-chromen-7-yl)oxy)-N-(quinolin-8-yl)hept-3-enamide (S32) : Pale yellow solid, 68% yield. **^{1}H NMR** (400 MHz, Chloroform-d) δ 10.0 (d, J = 36.0 Hz, 1H), 8.9 – 8.5 (m, 2H), 7.7 – 7.4 (m, 4H), 7.4 – 7.3 (m, 2H), 6.9 – 6.7 (m, 2H), 6.3 – 6.0 (m, 1H), 6.0 – 5.7 (m, 2H), 4.1 (dt, J = 23.2, 6.2 Hz, 2H), 3.3 (dd, J = 25.1, 6.0 Hz, 2H), 2.5 – 2.3 (m, 2H), 2.1 – 1.9 (m, 2H). **^{13}C NMR** (126 MHz, Chloroform-d) δ 169.8, 162.2, 161.3, 155.8, 148.2, 143.4, 138.5, 136.4, 135.3, 134.3, 128.7, 127.9, 127.4, 123.7, 121.6, 116.4, 113.0, 112.9, 112.4, 101.4, 67.8, 42.1, 29.0, 28.5. **HRMS:** calculated for $C_{25}H_{22}N_2O_4Na^+$ [M+Na$^+$]: 437.1472; found: 437.1469.

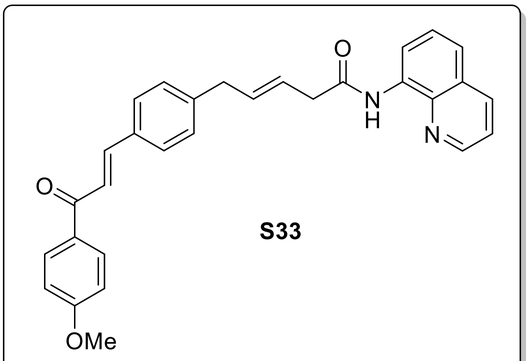

(*E*)-5-(4-((*E*)-3-(4-methoxyphenyl)-3-oxoprop-1-en-1-yl)phenyl)-N-(quinolin-8-yl)pent-3-en-amide (S33) : Yellow liquid, 74% yield. **^{1}H NMR** (400 MHz, Chloroform-d) δ 10.0 (s, 1H), 9.0 – 8.4 (m, 2H), 8.2 (dd, J = 8.3, 1.7 Hz, 1H), 8.1 – 7.9 (m, 2H), 7.8 (d, J = 15.6 Hz, 1H), 7.6 – 7.4 (m, 6H), 7.3 (d, J = 7.9 Hz, 2H), 7.1 – 6.7 (m, 2H), 6.1 – 5.4 (m, 2H), 3.9 (s, 3H), 3.5 (d, J = 6.4 Hz, 2H), 3.3 (dd, J = 6.9, 1.2 Hz, 2H).

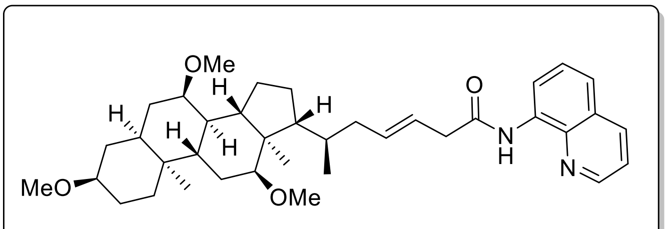

(*R*,*E*)-N-(quinolin-8-yl)-6-((3R,5S,7R,8R,9S,10S,12S,13R,14S,17R)-3,7,12-trimethoxy-10,13-dimethylhexadecahydro-1H-cyclopenta[a]phenanthren-17-yl)hept-3-enamide (S34) : Pale yellow solid, 54% yield. **^{1}H NMR** (400 MHz, Chloroform-d) δ 10.0 (s, 1H), 9.1 – 8.6 (m, 2H), 8.1 (dd, J = 8.2, 1.7 Hz, 1H), 7.7 – 7.3 (m, 3H), 6.0 – 5.6 (m, 2H), 3.4 (d, J = 2.7 Hz, 1H), 3.3 (s, 3H),

3.3 (d, J = 6.6 Hz, 2H), 3.2 (d, J = 10.5 Hz, 6H), 3.1 (d, J = 3.2 Hz, 1H), 3.1 – 2.9 (m, 1H), 2.2 – 1.4 (m, 17H), 1.4 – 1.2 (m, 3H), 1.1 – 1.0 (m, 5H), 0.9 (s, 3H), 0.7 (s, 3H).

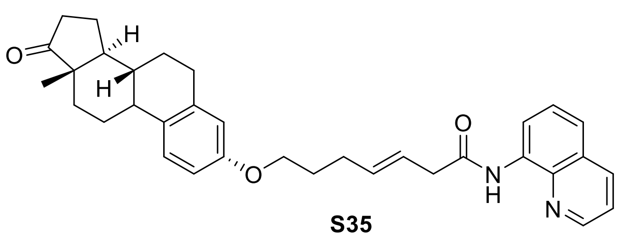

S35

(*E*)-7-(((8R,13S,14S)-13-methyl-17-oxo-7,8,9,11,12,13,14,15,16,17-decahydro-6H-cyclopenta[a]phenanthren-3-yl)oxy)-N-(quinolin-8-yl)hept-3-enamide (S35) : Yellow liquid, 66% yield. **^{1}H NMR** (400 MHz, Chloroform-d) δ 10.1 (s, 1H), 9.0 – 8.6 (m, 2H), 8.2 (dt, J = 8.4, 1.7 Hz, 1H), 7.7 – 7.4 (m, 3H), 7.2 (t, J = 8.8 Hz, 1H), 6.8 – 6.4 (m, 2H), 6.0 – 5.6 (m, 2H), 4.0 – 3.8 (m, 2H), 3.3 (d, J = 6.5 Hz, 2H), 3.0 – 2.8 (m, 2H), 2.0 – 1.4 (m, 16H), 1.3 (t, J = 7.1 Hz, 3H). **^{13}C NMR** (126 MHz, Chloroform-d) δ 221.1, 170.1, 157.1, 148.3, 138.6, 137.7, 136.7, 136.3, 134.4, 131.9, 127.9, 127.4, 126.3, 122.8, 121.6, 121.6, 116.4, 114.6, 112.1, 112.1, 67.7, 50.4, 48.0, 44.0, 42.1, 38.6, 38.4, 35.9, 32.4, 31.6, 29.7, 29.6, 28.9, 26.6, 25.9, 25.8, 21.6, 13.9. **HRMS:** calculated for $C_{34}H_{38}N_2O_3Na^+$ [M+Na$^+$]: 545.2775; found: 545.2770.

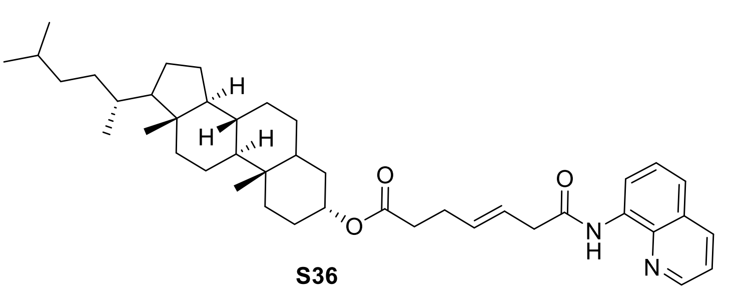

S36

(3R,8R,9S,10S,13R,14S)-10,13-dimethyl-17-((R)-5-methylhexan-2-yl)hexadecahydro-1H-cyclopenta[a]phenanthren-3-yl (*E*)-7-oxo-7-(quinolin-8-ylamino)hept-4-enoate (S36) : Yellow liquid, 83% yield. **^{1}H NMR** (400 MHz, Chloroform-d) δ 10.0 (s, 1H), 9.0 – 8.6 (m, 2H), 8.1 (dd, J = 8.2, 1.8 Hz, 1H), 7.9 – 7.3 (m, 3H), 6.1 – 5.7 (m, 2H), 4.7 (tt, J = 11.3, 4.8 Hz, 1H), 3.4 (d, J = 6.8 Hz, 1H), 2.6 – 2.4 (m, 4H), 1.9 (dt, J = 12.6, 3.6 Hz, 1H), 1.8 (tdd, J = 14.2, 5.6, 3.0 Hz, 2H), 1.7 – 1.6 (m, 2H), 1.6 – 1.5 (m, 3H), 1.4 – 0.8 (m, 34H), 0.6 (s, 3H). **^{13}C NMR** (101 MHz, Chloroform-d) δ 172.6, 169.6, 148.3, 138.5, 136.3, 134.8, 134.4, 127.9, 127.4, 123.7, 121.6, 121.5, 116.4, 56.4, 56.3, 54.2, 44.6, 42.6, 42.0, 40.0, 39.5, 36.7, 36.2, 35.8, 35.5, 35.4, 34.4, 34.1, 32.0, 28.6, 28.2, 28.1, 28.0, 27.5, 24.2, 23.8, 22.8, 22.6, 21.2, 18.7, 12.2, 12.1. **HRMS:** calculated for $C_{42}H_{60}N_2O_3Na^+$ [M+Na$^+$]: 663.4496; found: 663.4486.

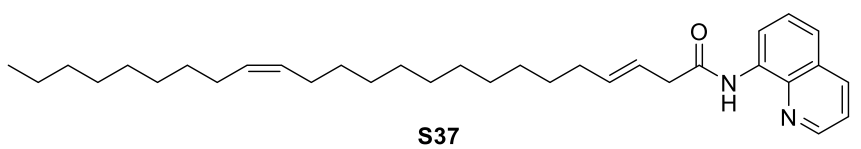

S37

(3*E*,15*Z*)-N-(quinolin-8-yl)tetracosa-3,15-dienamide (S37) : White solid, 77% yield. **^{1}H NMR** (500 MHz, Chloroform-d) δ 10.1 (s, 1H), 8.8 (dd, J = 5.1, 2.9 Hz, 2H), 8.2 (dd, J = 8.2, 1.9 Hz, 1H), 7.7 – 7.4 (m, 3H), 6.0 – 5.7 (m, 2H), 5.4 (t, J = 5.0 Hz, 2H), 3.3 (d, J = 7.0 Hz, 2H), 2.2 (q, J = 7.1 Hz, 2H), 2.0 (q, J = 6.5 Hz, 4H), 1.5 (p, J = 7.4 Hz, 2H), 1.4 – 1.2 (m, 26H), 0.9 (t, J = 6.9 Hz, 3H). **^{13}C NMR** (126 MHz, Chloroform-d) δ 170.1, 148.1, 138.6, 137.3, 136.3, 134.5, 129.9, 129.9, 127.9, 127.4, 122.2, 121.5, 121.5, 116.4, 42.2, 32.8, 31.9, 29.8, 29.7, 29.6, 29.6, 29.6, 29.5, 29.3, 29.3, 27.2, 22.7, 14.1. **HRMS:** calculated for $C_{33}H_{50}N_2ONa^+$ [M+Na$^+$]: 513.3815; found: 513.3814.

3. Supplementary Table 1. Investigation of various azo compounds with 1a.

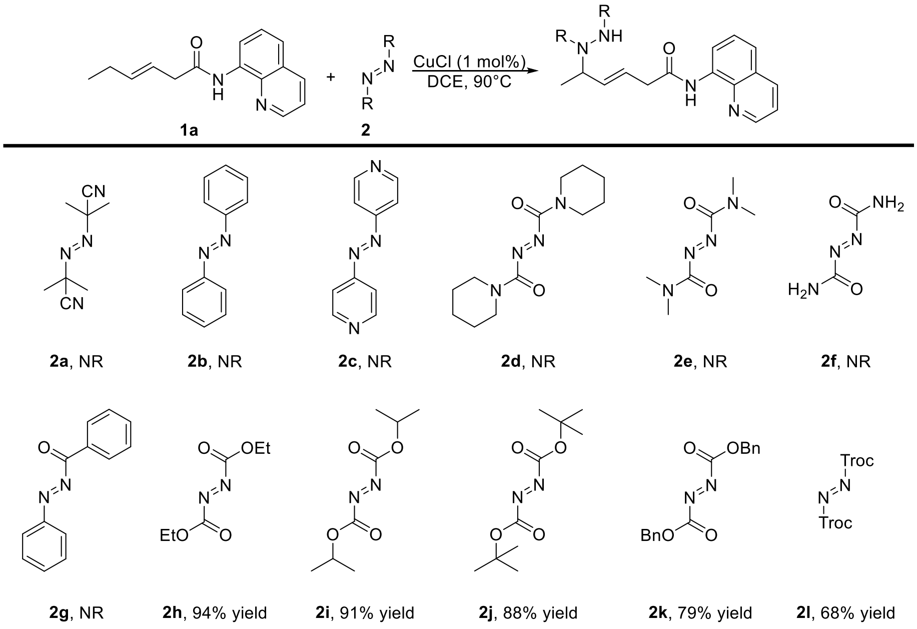

4. General procedure for Cu-catalyzed allylic C−H amination reactions

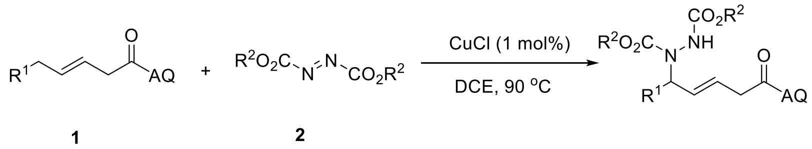

A mixture of amide (0.40 mmol, 1.0 equiv), CuCl (0.0040 mmol, 0.01 equiv), and azodicarboxylate (0.80 mmol, 2.0 equiv) in DCE (2 mL) in a 10 mL glass vial (sealed with PTFE cap) was heated at 90 °C for indicated time. The reaction progress was monitored by thin layer chromatography. Upon completion, the reaction mixture was concentrated *in vacuo* and purified by silica gel column

chromatography to afford the desired products.

Di-isopropyl (*E*)-1-(6-oxo-6-(quinolin-8-ylamino)hex-3-en-2-yl)hydrazine-1,2-dicarboxylate

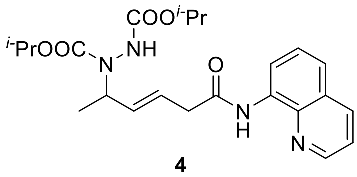

4

Compound **4** was isolated in 91 % yield (160.8 mg) with CuCl for 8 h. Flash silica gel chromatography (PE : EtOAc = 2:1). Colorless liquid. **^{1}H NMR** (400 MHz, Chloroform-d) δ 9.9 (s, 1H), 8.9 – 8.6 (m, 2H), 8.3 – 8.0 (m, 1H), 7.6 – 7.4 (m, 4H), 6.1 – 5.7 (m, 2H), 5.2 – 4.6 (m, 3H), 3.3 (t, J = 3.5 Hz, 2H), 1.5 – 1.3 (m, 3H), 1.3 – 1.0 (m, 12H). **^{13}C NMR** (101 MHz, Chloroform-*d*) δ 168.9, 156.4, 155.1, 148.5, 138.1, 136.5, 135.9, 133.9, 127.8, 127.1, 124.4, 121.7, 121.3, 116.5, 69.9, 69.1, 53.9, 41.6, 21.9, 21.7, 21.6, 16.5. **HRMS**: calculated for $C_{23}H_{30}N_4O_5$ [M+H$^+$] 443.2289, found 443.2290.

Di-tert-butyl (*E*)-1-(6-oxo-6-(quinolin-8-ylamino)hex-3-en-2-yl)hydrazine-1,2-dicarboxylate

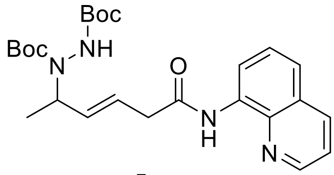

5

Compound **5** was isolated in 88 % yield (165.5 mg) with CuCl for 8 h. Flash silica gel chromatography (PE : EtOAc = 4:1). Yellow solid, m.p. 96 – 98 °C. **^{1}H NMR** (400 MHz, Chloroform-*d*) δ 9.9 (s, 1H), 9.0 – 8.6 (m, 2H), 8.1 (s, 1H), 7.6 – 7.1 (m, 4H), 5.9 (d, *J* = 7.1 Hz, 2H), 5.0 (s, 1H), 3.3 (d, *J* = 6.6 Hz, 2H), 1.8 – 0.9 (m, 21H). **^{13}C NMR** (101 MHz, Chloroform-*d*) δ 168.8, 155.5, 154.4, 148.3, 138.0, 136.3, 136.1, 133.8, 127.7, 126.9, 121.5, 121.1, 116.3, 80.8, 80.1, 52.5, 41.5, 28.0, 27.8, 16.4. **HRMS**: calculated for $C_{25}H_{34}N_4O_5$ [M+H$^+$] 471.2602, found 471.2600.

Dibenzyl (*E*)-1-(6-oxo-6-(quinolin-8-ylamino)hex-3-en-2-yl)hydrazine-1,2-dicarboxylate

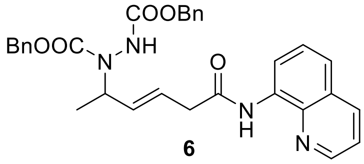

6

Compound **6** was isolated in 79 % yield (170.0 mg) with CuCl for 8 h. Flash silica gel chromatography (PE : EtOAc = 3:1). Yellow liquid. **^{1}H NMR** (400 MHz, Chloroform-*d*) δ 9.8 (s, 1H), 9.0 – 8.4 (m, 2H), 8.2 – 7.9 (m, 2H), 7.7 – 7.4 (m, 2H), 7.4 – 6.9 (m, 11H), 5.9 (s, 2H), 5.4 – 4.7 (m, 5H), 3.6 – 2.8 (m, 2H), 1.6 – 0.9 (m, 3H). **^{13}C NMR** (101 MHz, Chloroform-*d*) δ 168.8, 156.7, 155.4, 148.8, 138.1, 136.6, 135.8, 135.6, 133.9, 128.5, 128.4, 128.2, 128.1, 128.0, 127.9, 127.3, 121.9, 121.4, 116.8, 68.2, 67.4, 41.7, 16.5. **HRMS**: calculated for $C_{31}H_{30}N_4O_5$ [M+H$^+$] 539.2289, found 539.2292.

Bis(2,2,2-trichloroethyl) (*E*)-1-(6-oxo-6-(quinolin-8-ylamino)hex-3-en-2-yl)hydrazine-1,2-dicarboxylate

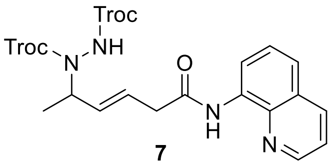

7

Compound **7** was isolated in 68 % yield (167.8 mg) with CuCl for 8 h. Flash silica gel chromatography (PE : EtOAc = 2:1). Yellow liquid. **^{1}H NMR** (400 MHz, Chloroform-*d*) δ 9.8 (s, 1H), 9.0 – 8.7 (m, 2H), 8.2 (dd, *J* = 8.3, 1.7 Hz, 1H), 7.7 – 7.4 (m, 3H), 6.2 – 5.9 (m, 2H), 5.2 – 4.5 (m, 5H), 3.4 (d, *J* = 6.7 Hz, 2H), 1.7 – 1.4 (m, 3H). **^{13}C NMR** (101 MHz, Chloroform-*d*) δ 168.6, 154.8, 154.0, 149.2, 138.1, 137.1, 134.9, 133.8, 128.2, 127.5, 126.9, 122.2, 121.8, 117.2, 94.9, 94.7, 75.8, 41.8, 16.1. **HRMS**: calculated for $C_{21}H_{20}Cl_6N_4O_5$ [M+H$^+$] 618.9638, found 618.9628.

Diethyl (*E*)-1-(6-oxo-6-(quinolin-8-ylamino)hex-3-en-2-yl)hydrazine-1,2-dicarboxylate

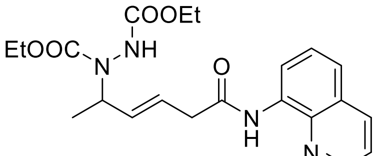

10

Compound **10** was isolated in 94 % yield (155.7 mg) with CuCl for 8 h. Flash silica gel chromatography (PE : EtOAc = 2:1). Colorless liquid. **^{1}H NMR** (400 MHz, Chloroform-d) δ 9.85 (s, 1H), 9.05 – 8.57 (m, 2H), 8.18 (dd, J = 8.3, 1.7 Hz, 1H), 7.76 – 7.33 (m, 4H), 6.17 – 5.68 (m, 2H), 4.98 (s, 1H), 4.42 – 3.83 (m, 4H), 3.32 (d, J = 5.6 Hz, 2H), 1.40 (d, J = 6.9 Hz, 3H), 1.38 – 0.98 (m, 6H). **^{13}C NMR** (101 MHz, Chloroform-d) δ 168.9, 156.9, 155.7, 148.8, 138.3, 136.7, 135.8, 134.0, 128.1, 127.3, 125.0, 121.9, 121.5, 116.8, 62.5, 61.8, 54.6, 41.8, 16.6, 14.5, 14.4. **HRMS**: calculated for $C_{21}H_{26}N_4O_5$ [M+H$^+$] 415.1976, found 415.1976.

Diethyl (*E*)-1-(5-oxo-5-(quinolin-8-ylamino)pent-2-en-1-yl)hydrazine-1,2-dicarboxylate

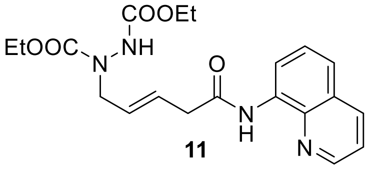

11

Compound **11** was isolated in 69 % yield (110.5 mg) with CuCl for 8 h. Flash silica gel chromatography (PE : EtOAc = 3:1). Colorless liquid. **^{1}H NMR** (400 MHz, Chloroform-*d*) δ 9.8 (s, 1H), 9.0 – 8.6 (m, 2H), 8.2 (d, *J* = 8.2 Hz, 1H), 8.1 – 7.6 (m, 1H), 7.6 – 7.4 (m, 3H), 6.1 – 5.8 (m, 2H), 4.4 – 3.8 (m, 6H), 3.3 (d, *J* = 6.5 Hz, 2H), 1.3 – 1.0 (m, 6H). **^{13}C NMR** (101 MHz, Chloroform-*d*) δ 168.8, 156.1, 148.8, 138.3, 136.9, 134.0, 130.6, 128.1, 127.4, 122.0, 121.6, 116.9, 62.6, 61.7, 51.7, 41.7, 14.6, 14.3. **HRMS**: calculated for $C_{20}H_{24}N_4O_5$ [M+H$^+$] 401.1819, found 401.1820.

Diethyl (*E*)-1-(2-methyl-6-oxo-6-(quinolin-8-ylamino)hex-3-en-2-yl)hydrazine-1,2-dicarboxylate

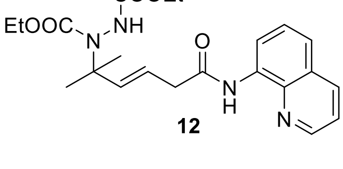

12

Compound **12** was isolated in 82 % yield (140.5 mg) with CuCl for 8 h. Flash silica gel chromatography (PE : EtOAc = 3:1). Colorless liquid. **^{1}H NMR** (400 MHz, Chloroform-*d*) δ 10.0 (s, 1H), 9.1 – 8.4 (m, 2H), 8.1 (dd, *J* = 8.2, 1.8 Hz, 1H), 7.6 – 7.4 (m, 3H), 7.3 – 6.9 (m, 1H),

6.3 – 6.0 (m, 1H), 6.0 – 5.7 (m, 1H), 4.4 – 4.0 (m, 4H), 3.3 (d, *J* = 7.3 Hz, 2H), 1.7 (s, 3H), 1.6 (s, 3H), 1.4 – 0.8 (m, 6H). **^{13}C NMR** (101 MHz, Chloroform-*d*) δ 169.5, 157.0, 155.6, 148.1, 142.3, 138.3, 136.4, 134.2, 127.9, 127.2, 121.6, 121.5, 119.7, 116.4, 62.3, 61.9, 61.7, 41.7, 26.4, 26.1, 14.4, 14.3. **HRMS**: calculated for $C_{22}H_{28}N_4O_5$ [M+H$^+$] 429.2132, found 429.2131.

Diethyl-1-(3-(2-oxo-2-(quinolin-8-ylamino)ethyl)cyclohex-2-en-1-yl)hydrazine-1,2-dicarboxylate

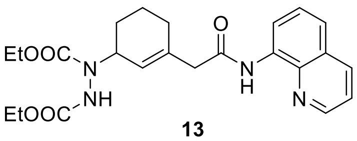

13

Compound **13** was isolated in 83 % yield (146.2 mg) with CuCl for 8 h. Flash silica gel chromatography (PE : EtOAc = 4:1). White solid, m.p. 116 – 119 °C. **^{1}H NMR** (400 MHz, Chloroform-*d*) δ 10.0 (s, 1H), 9.0 – 8.6 (m, 2H), 8.2 (d, *J* = 8.2 Hz, 1H), 7.6 – 7.4 (m, 3H), 5.7 (d, *J* = 39.5 Hz, 1H), 5.0 (s, 1H), 4.4 – 3.8 (m, 4H), 3.4 – 3.1 (m, 2H), 2.2 – 1.7 (m, 6H), 1.4 – 0.9 (m, 6H). **^{13}C NMR** (101 MHz, Chloroform-*d*) δ 168.9, 156.8, 155.9, 148.7, 138.4, 136.6, 134.1, 128.0, 127.3, 126.8, 121.8, 121.5, 116.7, 62.4, 61.5, 54.3, 47.5, 28.1, 25.8, 20.7, 14.5, 14.1. **HRMS**: calculated for $C_{23}H_{28}N_4O_5$ [M+H$^+$] 441.2132, found 441.2131.

Diethyl (*E*)-1-(5,5-dimethyl-6-oxo-6-(quinolin-8-ylamino)hex-3-en-2-yl)hydrazine-1,2-dicarboxylate

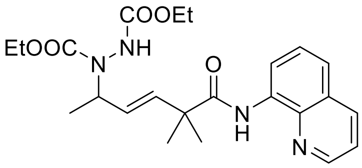

14

Compound **14** was isolated in 58 % yield (102.6 mg) with CuCl for 8 h. Flash silica gel chromatography (PE : EtOAc = 2:1). Yellow liquid. **^{1}H NMR** (400 MHz, Chloroform-*d*) δ 9.9 (s, 1H), 9.2 – 8.7 (m, 2H), 8.2 (d, *J* = 8.2 Hz, 1H), 7.8 – 7.4 (m, 4H), 6.1 – 5.9 (m, 2H), 5.0 (s, 1H), 4.3 – 3.9 (m, 4H), 1.5 – 1.4 (m, 9H), 1.3 – 1.0 (m, 6H). **^{13}C NMR** (101 MHz, Chloroform-*d*) δ 174.7, 156.9, 155.7, 148.9, 138.5, 136.8, 134.3, 130.1, 128.1, 127.4, 121.7, 121.4, 116.7, 62.4, 61.6, 46.2, 25.3, 25.2, 16.6, 14.5, 14.3. **HRMS**: calculated for $C_{23}H_{30}N_4O_5$ [M+H$^+$] 443.2289, found 443.2290.

Diethyl (*E*)-1-(5-methyl-6-oxo-6-(quinolin-8-ylamino)hex-3-en-2-yl)hydrazine-1,2-dicarboxylate

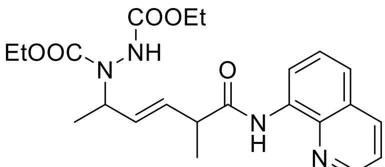

15

Compound **15** was isolated in 79 % yield (135.3 mg) with CuCl for 8 h. Flash silica gel chromatography (PE : EtOAc = 3:1). Yellow liquid. **^{1}H NMR** (400 MHz, Chloroform-*d*) δ 9.9 (s, 1H), 9.1 – 8.7 (m, 2H), 8.2 (d, *J* = 8.3 Hz, 1H), 7.7 – 7.3 (m, 4H), 6.0 – 5.8 (m, 2H), 5.0 (s, 1H), 4.4 – 3.9 (m, 4H), 3.4 – 3.3 (m, 1H), 1.5 – 1.0 (m, 12H). **^{13}C NMR** (126 MHz,

Chloroform-*d*) δ 172.3, 156.9, 155.7, 148.8, 138.4, 138.4, 136.8, 134.2, 133.1, 132.8, 128.1, 127.3, 121.8, 121.5, 116.7, 62.4, 61.8, 45.6, 16.9, 14.5, 14.2. **HRMS**: calculated for $C_{22}H_{28}N_4O_5$ [M+H$^+$] 429.2132, found 429.2117.

Diethyl (*E*)-1-(8-oxo-8-(quinolin-8-ylamino)oct-5-en-4-yl)hydrazine-1,2-dicarboxylate

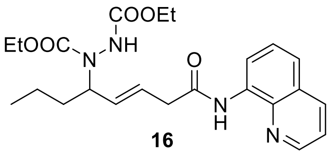

16

Compound **16** was isolated in 91 % yield (161.0 mg) with CuCl for 8 h. Flash silica gel chromatography (PE : EtOAc = 3:1). Colorless liquid. **^{1}H NMR** (400 MHz, Chloroform-*d*) δ 9.8 (s, 1H), 9.2 – 8.5 (m, 2H), 8.2 (dd, *J* = 8.3, 1.7 Hz, 1H), 7.7 – 7.4 (m, 4H), 6.4 – 5.3 (m, 2H), 4.7 (s, 1H), 4.5 – 3.8 (m, 4H), 3.8 – 2.9 (m, 2H), 1.9 – 1.4 (m, 4H), 1.4 – 1.1 (m, 6H), 1.0 (t, *J* = 7.3 Hz, 3H). **^{13}C NMR** (101 MHz, Chloroform-*d*) δ 169.0, 156.9, 156.0, 148.8, 138.3, 136.7, 135.0, 134.0, 128.1, 127.3, 126.0, 121.9, 121.5, 116.7, 62.4, 61.7, 59.1, 41.9, 33.6, 19.4, 14.5, 14.4, 13.9. **HRMS**: calculated for $C_{23}H_{30}N_4O_5$ [M+H$^+$] 443.2289, found 443.2285.

Diethyl (*E*)-1-(1-oxo-1-(quinolin-8-ylamino)tetradec-3-en-5-yl)hydrazine-1,2-dicarboxylate

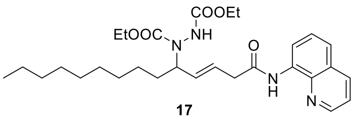

17

Compound **17** was isolated in 90 % yield (189.5 mg) with CuCl for 8 h. Flash silica gel chromatography (PE : EtOAc = 2:1). Colorless liquid. **^{1}H NMR** (400 MHz, Chloroform-*d*) δ 9.8 (s, 1H), 9.1 – 8.7 (m, 2H), 8.2 (dd, *J* = 8.3, 1.7 Hz, 1H), 7.7 – 7.3 (m, 4H), 6.1 – 5.8 (m, 2H), 4.7 (s, 1H), 4.3 – 4.0 (m, 4H), 3.3 (dd, *J* = 6.9, 2.6 Hz, 2H), 2.0 – 1.6 (m, 2H), 1.4 – 1.1 (m, 20H), 0.9 (t, *J* = 6.8 Hz, 3H). **^{13}C NMR** (101 MHz, Chloroform-*d*) δ 168.9, 156.8, 155.9, 148.7, 138.2, 136.6, 135.0, 134.0, 128.0, 127.2, 125.9, 121.8, 121.5, 116.7, 62.3, 61.6, 59.3, 41.9, 31.8, 31.4, 29.5, 29.5, 29.4, 29.2, 26.1, 22.6, 14.4, 14.3, 14.3, 14.0. **HRMS**: calculated for $C_{29}H_{42}N_4O_5$ [M+H$^+$] 527.3228, found 527.3231.

Diethyl (*E*)-1-(5-oxo-1-phenyl-5-(quinolin-8-ylamino)pent-2-en-1-yl)hydrazine-1,2-dicarboxylate

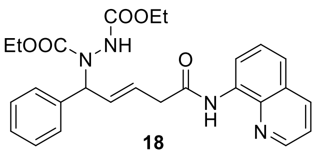

18

Compound **18** was isolated in 77 % yield (146.7 mg) with CuCl for 8 h. Flash silica gel chromatography (PE : EtOAc = 4:1). Yellow liquid. **^{1}H NMR** (400 MHz, Chloroform-*d*) δ 10.0 (s, 1H), 9.0 – 8.6 (m, 2H), 8.2 (dd, *J* = 8.3, 1.7 Hz, 1H), 7.6 – 7.4 (m, 5H), 7.4 – 7.1 (m, 3H), 6.5 – 6.1 (m, 1H), 6.1 – 5.6 (m, 2H), 4.4 – 3.8 (m, 4H), 3.4 (dd, *J* = 7.3, 2.1 Hz, 2H), 1.4 – 0.7 (m, 6H). **^{13}C NMR** (101 MHz, Chloroform-*d*) δ 168.9, 156.3, 155.8, 148.4, 138.5, 138.3, 136.5, 134.1, 133.1, 128.4, 128.1, 128.0, 127.8, 127.3, 126.9, 121.8, 121.5, 116.6, 63.8, 62.7, 61.8, 41.8, 14.4, 14.2. **HRMS**: calculated for $C_{26}H_{28}N_4O_5$ [M+H$^+$] 477.2132, found 477.2138.

Diethyl (*E*)-1-(1-(4-methoxyphenyl)-5-oxo-5-(quinolin-8-ylamino)pent-2-en-1-yl)hydrazine-1,2-dicarboxylate

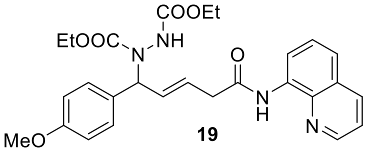

19

Compound **19** was isolated in 78 % yield (157.9 mg) with CuCl for 8 h. Flash silica gel chromatography (PE : EtOAc = 4:1). Yellow liquid. **^{1}H NMR** (400 MHz, Chloroform-*d*) δ 10.0 (s, 1H), 8.9 – 8.5 (m, 2H), 8.2 (dd, *J* = 8.3, 1.7 Hz, 1H), 7.8 – 7.3 (m, 5H), 6.9 (d, *J* = 8.4 Hz, 2H), 6.4 – 6.1 (m, 1H), 6.1 – 5.6 (m, 2H), 4.4 – 3.9 (m, 4H), 3.8 (s, 3H), 3.4 (d, *J* = 7.2 Hz, 2H), 1.3 – 0.9 (m, 6H). **^{13}C NMR** (101 MHz, Chloroform-*d*) δ 169.0, 159.1, 155.8, 148.4, 138.3, 136.5, 134.1, 133.6, 130.5, 129.5, 128.0, 127.3, 126.4, 121.8, 121.5, 116.6, 113.8, 62.6, 61.7, 55.2, 41.8, 14.4, 14.2. **HRMS**: calculated for $C_{27}H_{30}N_4O_6$ [M+H$^+$] 507.2238, found 507.2241.

Diethyl (*E*)-1-(1-([1,1'-biphenyl]-4-yl)-5-oxo-5-(quinolin-8-ylamino)pent-2-en-1-yl)hydrazine-1,2-dicarboxylate

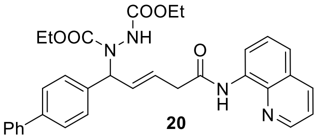

20

Compound **20** was isolated in 80 % yield (176.7 mg) with CuCl for 8 h. Flash silica gel chromatography (PE : EtOAc = 4:1).Yellow solid. **^{1}H NMR** (400 MHz, Chloroform-*d*) δ 10.0 (s, 1H), 9.0 – 8.7 (m, 2H), 8.2 (dd, *J* = 8.3, 1.7 Hz, 1H), 7.6 – 7.3 (m, 13H), 6.3 – 6.2 (m, 1H), 6.2 – 5.9 (m, 2H), 4.4 – 3.9 (m, 4H), 3.5 – 3.3 (m, 2H), 1.3 – 1.0 (m, 6H). **^{13}C NMR** (101 MHz, Chloroform-*d*) δ 168.9, 156.3, 155.8, 148.4, 140.6, 138.3, 137.6, 136.5, 134.1, 133.1, 128.7, 128.5, 127.9, 127.3, 127.1, 127.0, 121.8, 121.5, 116.6, 63.7, 62.7, 61.8, 41.8, 14.4, 14.2. **HRMS**: calculated for $C_{32}H_{32}N_4O_5$ [M+H$^+$] 553.2445, found 553.2443.

Diethyl (*E*)-1-(1-(4-acetylphenyl)-5-oxo-5-(quinolin-8-ylamino)pent-2-en-1-yl)hydrazine-1,2-dicarboxylate

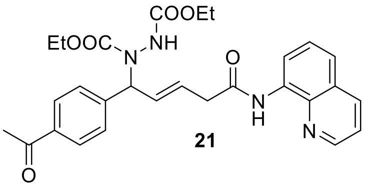

21

Compound **21** was isolated in 81 % yield (167.9 mg) with CuCl for 8 h. Flash silica gel chromatography (PE : EtOAc = 3:1). Yellow liquid. **^{1}H NMR** (400 MHz, Chloroform-*d*) δ 9.9 (s, 1H), 8.9 – 8.7 (m, 2H), 8.2 (dd, *J* = 8.3, 1.7 Hz, 1H), 7.9 (d, *J* = 8.2 Hz, 2H), 7.7 – 7.4 (m, 5H), 6.3 – 5.8 (m, 3H), 4.3 – 3.9 (m, 4H), 3.5 – 3.3 (m, 2H), 2.6 (s, 3H), 1.3 – 1.0 (m, 6H). **^{13}C NMR** (101 MHz, Chloroform-*d*) δ 197.7, 168.6, 156.4, 155.7, 148.5, 144.1, 138.3, 136.7, 136.4, 134.0, 132.3, 128.5, 128.1, 128.0, 127.9, 127.4, 121.9, 121.6, 116.8, 64.0, 62.8, 61.9, 41.7, 26.6, 14.4, 14.3. **HRMS**: calculated for $C_{28}H_{30}N_4O_6$ [M+H$^+$] 519.2238, found 519.2231.

Diethyl (*E*)-1-(1-(4-(methoxycarbonyl)phenyl)-5-oxo-5-(quinolin-8-ylamino)pent-2-en-1-yl)hydrazine-1,2-dicarboxylate

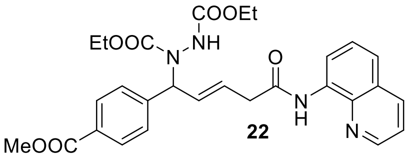

Compound **22** was isolated in 89 % yield (190.2 mg) with CuCl for 8 h. Flash silica gel chromatography (PE : EtOAc = 2:1).White solid, m.p. 88 – 90 °C. **^{1}H NMR** (400 MHz, Chloroform-*d*) δ 9.9 (s, 1H), 9.0 – 8.6 (m, 2H), 8.2 (dd, *J* = 8.3, 1.7 Hz, 1H), 8.0 (d, *J* = 8.1 Hz, 2H), 7.7 – 7.4 (m, 5H), 6.3 – 5.8 (m, 3H), 4.3 – 4.0 (m, 4H), 3.9 (s, 3H), 3.4 (dd, *J* = 7.2, 3.4 Hz, 2H), 1.3 – 1.0 (m, 6H). **^{13}C NMR** (101 MHz, Chloroform-*d*) δ 168.7, 166.8, 156.4, 155.7, 148.5, 143.9, 138.3, 136.7, 134.0, 132.4, 129.8, 129.6, 128.1, 128.0, 127.8, 127.4, 121.9, 121.6, 116.8, 62.9, 62.0, 52.1, 41.8, 14.4, 14.3. **HRMS**: calculated for $C_{28}H_{30}N_4O_7$ [M+H$^+$] 535.2187, found 535.2183.

Diethyl (*E*)-1-(1-(benzo[d][1,3]dioxol-5-yl)-5-oxo-5-(quinolin-8-ylamino)pent-2-en-1-yl)hydrazine-1,2-dicarboxylate

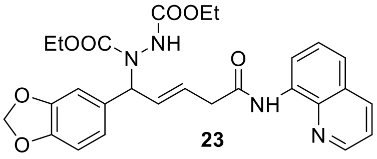

Compound **23** was isolated in 75 % yield (156.1 mg) with CuCl for 8 h. Flash silica gel chromatography (PE : EtOAc = 2:1).Yellow solid, m.p. 115 – 117 °C. **^{1}H NMR** (400 MHz, Chloroform-*d*) δ 10.0 (s, 1H), 9.0 – 8.7 (m, 2H), 8.2 (dd, *J* = 8.2, 1.7 Hz, 1H), 7.6 – 7.4 (m, 3H), 7.2 – 6.7 (m, 4H), 6.2 (dd, *J* = 15.6, 6.4 Hz, 1H), 6.1 – 6.0 (m, 1H), 5.9 (q, *J* = 1.4 Hz, 2H), 5.9 – 5.8 (m, 1H), 4.3 – 3.9 (m, 4H), 3.4 (d, *J* = 7.1 Hz, 2H), 1.3 – 1.0 (m, 6H). **^{13}C NMR** (101 MHz, Chloroform-*d*) δ 168.9, 156.3, 155.8, 148.4, 147.7, 147.1, 138.3, 136.6, 134.1, 133.3, 132.4, 128.0, 127.3, 126.7, 121.8, 121.6, 116.6, 108.7, 108.1, 101.0, 63.5, 62.7, 61.8, 41.8, 14.4, 14.2. **HRMS**: calculated for $C_{27}H_{28}N_4O_7$ [M+H$^+$] 521.2031, found 521.2031.

Diethyl (*E*)-1-(6-oxo-1-phenyl-6-(quinolin-8-ylamino)hex-3-en-2-yl)hydrazine-1,2-dicarboxylate

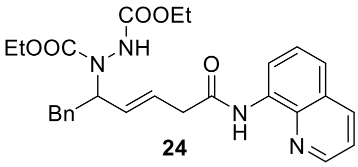

Compound **24** was isolated in 78 % yield (152.9 mg) with CuCl for 8 h. Flash silica gel chromatography (PE : EtOAc = 3:1). Yellow liquid. **^{1}H NMR** (400 MHz, Chloroform-*d*) δ 9.8 (s, 1H), 9.1 – 8.6 (m, 2H), 8.1 (dd, *J* = 8.3, 1.7 Hz, 1H), 7.6 – 7.4 (m, 4H), 7.3 – 7.1 (m, 5H), 6.1 – 5.6 (m, 2H), 5.0 (s, 1H), 4.4 – 3.8 (m, 4H), 3.5 – 2.6 (m, 4H), 1.5 – 0.6 (m, 6H). **^{13}C NMR** (101 MHz, Chloroform-*d*) δ 169.0, 157.1, 155.7, 148.7, 138.3, 138.0, 136.7, 134.1, 129.2, 128.5, 128.1, 127.3, 126.4, 121.9, 121.6, 116.7, 62.5, 61.9, 60.4, 41.8, 14.4. **HRMS**: calculated for $C_{27}H_{30}N_4O_5$ [M+H$^+$] 491.2289, found 491.2291.

Diethyl (*E*)-1-(1-(benzyloxy)-7-oxo-7-(quinolin-8-ylamino)hept-4-en-3-yl)hydrazine-1,2-dicarboxylate

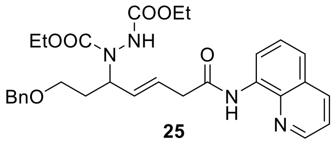

25

Compound **25** was isolated in 89 % yield (190.2 mg) with CuCl for 8 h. Flash silica gel chromatography (PE : EtOAc = 2:1). Yellow liquid. **^{1}H NMR** (400 MHz, Chloroform-d) δ 9.9 (s, 1H), 9.1 – 8.6 (m, 2H), 8.2 (dd, J = 8.3, 1.7 Hz, 1H), 7.6 – 7.5 (m, 2H), 7.5 – 7.4 (m, 1H), 7.4 – 7.2 (m, 5H), 6.3 – 5.5 (m, 2H), 5.0 (s, 1H), 4.7 – 4.4 (m, 2H), 4.4 – 3.9 (m, 4H), 3.7 (d, J = 29.5 Hz, 2H), 3.3 (d, J = 6.3 Hz, 2H), 2.4 – 2.1 (m, 1H), 2.0 – 1.8 (m, 1H), 1.8 – 0.7 (m, 6H). **^{13}C NMR** (101 MHz, Chloroform-*d*) δ 168.9, 156.8, 156.0, 148.6, 138.3, 138.2, 136.5, 134.5, 134.0, 128.2, 127.9, 127.6, 127.5, 127.2, 121.8, 121.5, 116.6, 73.0, 62.4, 61.6, 41.8, 31.5, 14.4, 14.3. **HRMS**: calculated for $C_{29}H_{34}N_4O_6$ [M+H$^+$] 535.2551, found 535.2552.

Diethyl (*E*)-1-(1-((tert-butyldimethylsilyl)oxy)-6-oxo-6-(quinolin-8-ylamino)hex-3-en-2-yl)hydrazine-1,2-dicarboxylate

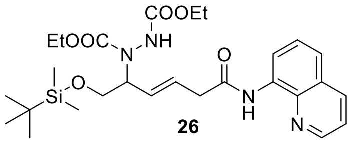

26

Compound **26** was isolated in 92 % yield (200.3 mg) with CuCl for 8 h. Flash silica gel chromatography (PE : EtOAc = 2:1). White solid, m.p. 75 – 77 °C. **^{1}H NMR** (400 MHz, Chloroform-*d*) δ 9.8 (s, 1H), 8.8 – 8.5 (m, 2H), 8.1 (dd, *J* = 8.3, 1.7 Hz, 1H), 7.5 – 7.3 (m, 3H), 6.2 – 5.6 (m, 2H), 4.8 (s, 1H), 4.2 – 4.0 (m, 4H), 3.9 – 3.6 (m, 2H), 3.4 – 3.0 (m, 2H), 1.3 – 1.1 (m, 6H), 0.8 (d, *J* = 1.3 Hz, 9H), 0.0 (d, *J* = 4.6 Hz, 6H). **^{13}C NMR** (101 MHz, Chloroform-*d*) δ 168.9, 156.1, 148.3, 138.3, 136.4, 134.0, 131.6, 127.9, 127.2, 126.7, 121.7, 121.5, 116.5, 63.9, 62.5, 62.2, 61.7, 60.7, 41.8, 25.7, 18.0, 14.4, 14.3, 14.3, 14.0, -5.5. **HRMS**: calculated for $C_{27}H_{40}N_4O_6Si$ [M+H$^+$] 545.2790, found 545.2800.

Diethyl (*E*)-1-(1-methoxy-1,8-dioxo-8-(quinolin-8-ylamino)oct-5-en-4-yl)hydrazine-1,2-dicarboxylate

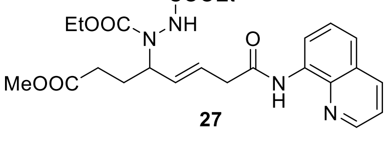

27

Compound **27** was isolated in 77 % yield (149.7 mg) with CuCl for 8 h. Flash silica gel chromatography (PE : EtOAc = 2:1). Yellow liquid. **^{1}H NMR** (400 MHz, Chloroform-*d*) δ 9.8 (s, 1H), 9.0 – 8.4 (m, 2H), 8.1 (d, *J* = 8.3 Hz, 1H), 7.9 – 7.4 (m, 4H), 6.2 – 5.5 (m, 2H), 4.8 (s, 1H), 4.4 – 4.0 (m, 4H), 3.7 (s, 3H), 3.3 (d, *J* = 6.8 Hz, 2H), 3.0 – 2.4 (m, 2H), 2.3 – 1.8 (m, 2H), 1.6 – 0.5 (m, 6H). **^{13}C NMR** (101 MHz, Chloroform-*d*) δ 173.8, 168.6, 156.7, 155.7, 148.6, 138.1, 136.4, 134.0, 133.8, 127.8, 127.0, 126.2, 121.7, 121.4, 116.4, 62.4, 61.6, 58.5, 51.4,

41.6, 30.5, 26.2, 14.3, 14.1. **HRMS**: calculated for $C_{24}H_{30}N_4O_7$ [M+H$^+$] 487.2187, found 487.2184.

Diethyl (*E*)-1-(1-ethoxy-2-(ethoxycarbonyl)-1,8-dioxo-8-(quinolin-8-ylamino)oct-5-en-4-yl)hydrazine-1,2-dicarboxylate

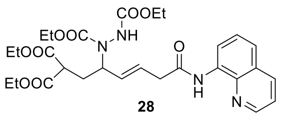

Compound **28** was isolated in 83 % yield (190.0 mg) with CuCl for 8 h. Flash silica gel chromatography (PE : EtOAc = 2:1). Yellow liquid. **^{1}H NMR** (400 MHz, Chloroform-*d*) δ 9.9 (s, 1H), 9.1 – 8.7 (m, 2H), 8.2 (dd, *J* = 8.3, 1.7 Hz, 1H), 7.6 – 7.4 (m, 3H), 6.1 – 5.8 (m, 2H), 4.9 (s, 1H), 4.3 – 4.0 (m, 8H), 3.8 (d, *J* = 57.9 Hz, 1H), 3.3 (d, *J* = 7.0 Hz, 2H), 2.6 – 2.1 (m, 2H), 1.3 – 1.1 (m, 12H). **^{13}C NMR** (101 MHz, Chloroform-*d*) δ 168.9, 156.8, 155.9, 148.7, 138.2, 136.6, 135.0, 134.0, 128.0, 127.2, 125.9, 121.8, 121.5, 116.7, 62.3, 61.6, 59.3, 41.9, 31.8, 31.4, 29.5, 29.5, 29.4, 29.2, 26.1, 22.6, 14.4, 14.3, 14.3, 14.0. **HRMS**: calculated for $C_{28}H_{36}N_4O_9$ [M+H$^+$] 573.2555, found 573.2554.

Diethyl-1-((2*R*,*E*)-2-((tert-butoxycarbonyl)amino)-1-methoxy-1,7-dioxo-7-(quinolin-8-ylamino)hept-4-en-3-yl)hydrazine-1,2-dicarboxylate

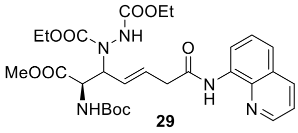

Compound **29** was isolated in 65 % yield (152.7 mg) with CuCl for 12 h. Flash silica gel chromatography (PE : EtOAc = 2:1). Yellow liquid. **^{1}H NMR** (400 MHz, Chloroform-*d*) δ 9.8 (s, 1H), 9.0 – 8.7 (m, 2H), 8.2 (dd, *J* = 8.2, 1.7 Hz, 1H), 7.7 – 7.4 (m, 4H), 6.3 – 5.3 (m, 3H), 4.9 (s, 1H), 4.5 (t, *J* = 9.0 Hz, 1H), 4.3 – 4.0 (m, 4H), 3.7 (s, 3H), 3.5 – 3.2 (m, 2H), 1.4 (s, 9H), 1.3 – 1.2 (m, 6H). **^{13}C NMR** (101 MHz, Chloroform-*d*) δ 170.5, 168.5, 156.5, 155.8, 148.5, 138.3, 136.5, 134.2, 128.6, 128.0, 127.3, 121.8, 121.6, 116.6, 80.5, 62.9, 62.0, 55.5, 52.4, 41.8, 28.2, 14.4, 14.4. **HRMS**: calculated for $C_{28}H_{37}N_5O_9$ [M+H$^+$] 588.2664, found 588.2664.

Diethyl (*E*)-1-(1-chloro-8-oxo-8-(quinolin-8-ylamino)oct-5-en-4-yl)hydrazine-1,2-dicarboxylate

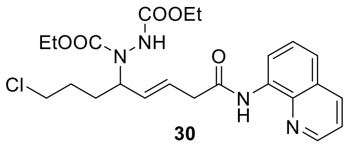

Compound **30** was isolated in 90 % yield (171.4 mg) with CuCl for 8 h. Flash silica gel chromatography (PE : EtOAc = 3:1). Colorless liquid. **^{1}H NMR** (500 MHz, Chloroform-*d*) δ 9.8 (s, 1H), 9.0 – 8.6 (m, 2H), 8.2 (dd, *J* = 8.4, 1.6 Hz, 1H), 7.6 – 7.4 (m, 4H), 6.3 – 5.5 (m, 2H), 4.8 (s, 1H), 4.5 – 3.9 (m, 4H), 3.7 – 3.5 (m, 2H), 3.3 (d, *J* = 7.1 Hz, 2H), 2.4 – 1.7 (m, 4H), 1.4 – 1.0 (m, 6H). **^{13}C NMR** (101 MHz, Chloroform-*d*) δ 168.7, 156.9, 155.9, 148.8, 138.2, 136.7, 134.4, 133.9, 128.0, 127.2, 126.5, 121.9, 121.5, 116.7, 62.6, 61.8, 58.5, 45.0, 41.8, 29.1, 28.8, 14.4, 14.3. **HRMS**: calculated for $C_{23}H_{29}ClN_4O_5$ [M+H$^+$] 477.1889, found 477.1902.

Diethyl (*E*)-1-(1-iodo-8-oxo-8-(quinolin-8-ylamino)oct-5-en-4-yl)hydrazine-1,2-dicarboxylate

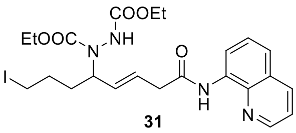

31

Compound **31** was isolated in 86 % yield (195.4 mg) with CuCl for 8 h. Flash silica gel chromatography (PE : EtOAc = 3:1). Colorless liquid. **^{1}H NMR** (400 MHz, Chloroform-*d*) δ 9.8 (s, 1H), 9.2 – 8.6 (m, 2H), 8.2 (dd, *J* = 8.2, 1.8 Hz, 1H), 7.7 – 7.4 (m, 4H), 5.9 (ddd, *J* = 46.3, 15.7, 7.6 Hz, 2H), 4.8 (s, 1H), 4.4 – 3.8 (m, 4H), 3.3 (t, *J* = 7.7 Hz, 4H), 2.2 – 1.6 (m, 4H), 1.5 – 0.8 (m, 6H). **^{13}C NMR** (101 MHz, Chloroform-*d*) δ 168.7, 157.0, 155.9, 148.8, 138.3, 136.8, 134.4, 134.0, 128.1, 127.3, 126.7, 122.0, 121.6, 116.8, 62.7, 61.9, 58.8, 51.3, 41.9, 28.7, 25.7, 14.5, 14.4. **HRMS**: calculated for $C_{23}H_{29}IN_4O_5$ [M+H$^+$] 569.1255, found 569.1253.

Diethyl (*E*)-1-(1-azido-8-oxo-8-(quinolin-8-ylamino)oct-5-en-4-yl)hydrazine-1,2-dicarboxylate

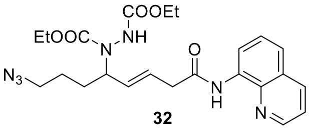

32

Compound **32** was isolated in 75 % yield (145.0 mg) with CuCl for 8 h. Flash silica gel chromatography (PE : EtOAc = 3:1). Yellow liquid. **^{1}H NMR** (400 MHz, Chloroform-*d*) δ 9.8 (s, 1H), 9.1 – 8.6 (m, 2H), 8.2 (dd, *J* = 8.2, 1.7 Hz, 1H), 7.7 – 7.4 (m, 4H), 6.4 – 5.3 (m, 2H), 4.8 (s, 1H), 4.3 – 3.9 (m, 4H), 3.5 – 3.0 (m, 4H), 2.2 – 1.6 (m, 4H), 1.5 – 1.0 (m, 6H). **^{13}C NMR** (101 MHz, Chloroform-*d*) δ 168.7, 156.9, 155.9, 148.8, 138.3, 136.8, 134.4, 133.9, 128.1, 127.3, 126.7, 122.0, 121.5, 116.8, 62.6, 61.8, 58.6, 41.9, 32.4, 29.9, 14.5, 14.4. **HRMS**: calculated for $C_{23}H_{29}N_7O_5$ [M+H$^+$] 484.2303, found 484.2297.

Diethyl (*E*)-1-(1-oxo-1-(quinolin-8-ylamino)trideca-3,12-dien-5-yl)hydrazine-1,2-dicarboxylate

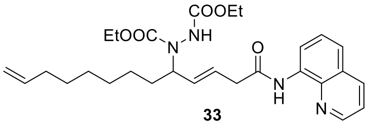

33

Compound **33** was isolated in 81 % yield (165.3 mg) with CuCl for 8 h. Flash silica gel chromatography (PE : EtOAc = 2:1). Yellow liquid. **^{1}H NMR** (400 MHz, Chloroform-*d*) δ 9.8 (s, 1H), 9.0 – 8.7 (m, 2H), 8.2 (dd, *J* = 8.3, 1.7 Hz, 1H), 7.8 – 7.3 (m, 4H), 6.2 – 5.6 (m, 3H), 5.2 – 4.8 (m, 2H), 4.7 (s, 1H), 4.3 – 4.0 (m, 4H), 3.3 (dd, *J* = 7.0, 2.9 Hz, 2H), 2.0 (d, J = 7.0 Hz, 2H), 1.9 – 1.6 (m, 2H), 1.5 – 0.9 (m, 14H). **^{13}C NMR** (101 MHz, Chloroform-*d*) δ 168.9, 156.8, 155.9, 148.8, 139.1, 138.3, 136.7, 135.1, 134.0, 128.1, 127.3, 121.9, 121.5, 116.8, 114.2, 62.5, 61.7, 59.5, 42.0, 33.7, 31.5, 29.3, 29.0, 28.9, 26.1, 14.5, 14.4. **HRMS**: calculated for $C_{28}H_{38}N_4O_5$ [M+H$^+$] 511.2915, found 511.2912.

Diethyl (*E*)-1-(9-oxo-9-(quinolin-8-ylamino)non-6-en-1-yn-5-yl)hydrazine-1,2-dicarboxylate

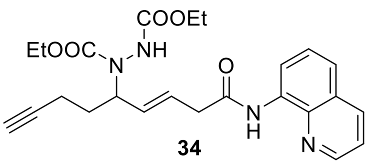

34

Compound **34** was isolated in 84 % yield (151.9 mg) with CuCl for 8 h. Flash silica gel chromatography (PE : EtOAc = 2:1). Colorless liquid. **^{1}H NMR** (400 MHz, Chloroform-*d*) δ 9.8 (s, 1H), 9.2 – 8.6 (m, 2H), 8.2 (dd, *J* = 8.3, 1.7 Hz, 1H), 7.7 – 7.3 (m, 4H), 6.2 – 5.8 (m, 2H), 4.9 (s, 1H), 4.4 – 4.0 (m, 4H), 3.3 (d, *J* = 7.0 Hz, 2H), 2.5 – 2.3 (m, 2H), 2.1 – 2.0 (m, 1H), 2.0 – 1.8 (m, 2H), 1.3 – 1.1 (m, 6H). **^{13}C NMR** (101 MHz, Chloroform-*d*) δ 168.8, 157.1, 155.9, 148.8, 138.3, 136.8, 134.2, 133.9, 128.1, 127.3, 126.5, 121.9, 121.6, 116.8, 83.9, 68.7, 62.6, 61.9, 58.2, 41.9, 30.3, 15.5, 14.5, 14.3. **HRMS**: calculated for $C_{24}H_{28}N_4O_5$ [M+H$^+$] 453.2132, found 453.2131.

Diethyl (*E*)-1-(8-oxo-8-(quinolin-8-ylamino)-1-(2,2,2-trifluoroacetamido)oct-5-en-4-yl)hydrazine-1,2-dicarboxylate

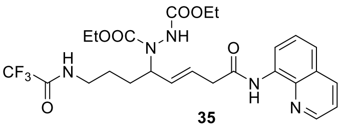

35

Compound **35** was isolated in 82 % yield (181.4 mg) with CuCl for 8 h. Flash silica gel chromatography (PE : EtOAc = 1:1). Yellow liquid. **^{1}H NMR** (400 MHz, Chloroform-*d*) δ 9.8 (s, 1H), 9.1 – 8.7 (m, 2H), 8.3 – 8.1 (m, 1H), 7.8 (s, 1H), 7.6 – 7.4 (m, 4H), 6.1 – 5.8 (m, 2H), 4.8 (d, *J* = 53.8 Hz, 1H), 4.4 – 3.9 (m, 4H), 3.4 (dd, *J* = 25.2, 5.7 Hz, 4H), 2.1 – 1.5 (m, 4H), 1.3 – 1.1 (m, 6H).**^{13}C NMR** (101 MHz, Chloroform-*d*) δ 169.0, 156.8, 156.0, 148.8, 138.4, 136.8, 135.2, 134.1, 129.9, 128.1, 127.4, 121.9, 121.6, 116.8, 62.5, 61.8, 59.3, 42.0, 31.9, 31.5, 29.8, 29.8, 29.7, 29.7, 29.6, 29.6, 29.6, 29.5, 29.5, 29.3, 29.3, 27.2, 26.2, 22.7, 14.5, 14.4, 14.1. **^{19}F NMR** (376 MHz, Chloroform-d) δ -75.6. **HRMS**: calculated for $C_{25}H_{30}F_3N_5O_6$ [M+H$^+$] 554.2221, found 554.2215.

Diethyl (*E*)-1-(1-(1-(ethoxycarbonyl)-4-oxocyclohexyl)-6-oxo-6-(quinolin-8-ylamino)hex-3-en-2-yl)hydrazine-1,2-dicarboxylate

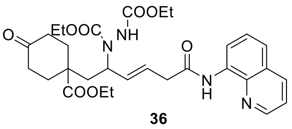

36

Compound **36** was isolated in 81 % yield (188.6 mg) with CuCl for 8 h. Flash silica gel chromatography (PE : EtOAc = 1:1). Colorless liquid. **^{1}H NMR** (400 MHz, Chloroform-*d*) δ 9.8 (s, 1H), 9.0 – 8.7 (m, 2H), 8.2 (dd, *J* = 8.2, 1.7 Hz, 1H), 7.6 – 7.4 (m, 3H), 6.1 – 5.8 (m, 2H), 5.0 (s, 1H), 4.3 – 4.0 (m, 6H), 3.4 – 3.2 (m, 2H), 2.6 – 2.2 (m, 7H), 2.1 – 1.9 (m, 1H), 1.9 – 1.7 (m, 2H), 1.3 – 1.1 (m, 9H). **^{13}C NMR** (101 MHz, Chloroform-*d*) δ 210.6, 176.0, 168.8, 156.6, 155.2, 148.5, 138.3, 136.6, 134.1, 128.0, 127.4, 121.9, 121.6, 116.7, 62.6, 61.9, 61.5, 55.8, 44.2, 41.8, 38.0, 14.5, 14.3, 14.1. **HRMS**: calculated for $C_{30}H_{38}N_4O_8$ [M+H$^+$] 583.2762, found 583.2765.

Diethyl (*E*)-1-(1-(1H-indol-3-yl)-6-oxo-6-(quinolin-8-ylamino)hex-3-en-2-yl)hydrazine-1,2-dicarboxylate

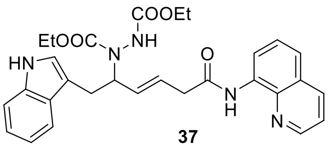

37

Compound **37** was isolated in 72 % yield (158.7 mg) with CuCl for 8 h. Flash silica gel chromatography (PE : EtOAc = 3:1). Colorless liquid. **^{1}H NMR** (500 MHz, Chloroform-*d*) δ 9.9 (s, 1H), 9.1 – 8.6 (m, 2H), 8.3 – 7.9 (m, 2H), 7.7 (d, *J* = 7.8 Hz, 1H), 7.6 – 7.4 (m, 3H), 7.3 (d, *J* = 8.0 Hz, 1H), 7.2 – 7.0 (m, 3H), 6.1 – 5.8 (m, 2H), 5.2 (s, 1H), 4.3 – 3.9 (m, 4H), 3.5 – 2.9 (m, 4H), 1.3 – 1.0 (m, 6H). **^{13}C NMR** (126 MHz, Chloroform-*d*) δ 169.1, 148.6, 138.4, 136.8, 136.3, 134.1, 128.1, 127.6, 127.4, 122.0, 121.9, 121.6, 119.4, 118.7, 116.8, 112.1, 111.2, 62.5, 62.0, 59.9, 41.9, 14.4. **HRMS**: calculated for $C_{29}H_{31}N_5O_5$ [M+Na^{+}] 552.2217, found 552.2221.

Diethyl (*E*)-1-(1-(1-(tert-butoxycarbonyl)piperidin-4-yl)-5-oxo-5-(quinolin-8-ylamino)pent-2-en-1-yl)hydrazine-1,2-dicarboxylate

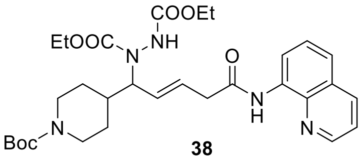

38

Compound **38** was isolated in 74 % yield (172.6 mg) with CuCl for 8 h. Flash silica gel chromatography (PE : EtOAc = 2:1). Yellow solid, m.p. 136 – 138 °C. **^{1}H NMR** (400 MHz, Chloroform-*d*) δ 9.8 (s, 1H), 9.2 – 8.8 (m, 2H), 8.2 (dd, *J* = 8.3, 1.7 Hz, 1H), 7.7 – 7.3 (m, 4H), 6.1 – 5.8 (m, 2H), 4.4 – 4.0 (m, 7H), 3.5 – 3.2 (m, 2H), 2.9 – 2.5 (m, 2H), 2.4 – 2.1 (m, 1H), 1.9 – 1.6 (m, 2H), 1.5 (s, 9H), 1.3 – 1.2 (m, 8H). **^{13}C NMR** (101 MHz, Chloroform-*d*) δ 171.2, 168.7, 156.0, 154.8, 148.9, 138.3, 136.8, 133.9, 133.3, 128.1, 127.3, 122.0, 121.6, 116.9, 79.3, 62.6, 61.9, 60.4, 43.9, 42.0, 37.9, 29.4, 28.5, 21.0, 14.5, 14.4, 14.2. **HRMS**: calculated for $C_{30}H_{41}N_5O_7$ [M+H^{+}] 584.3079, found 584.3082.

Diethyl (*E*)-1-(1-(1,3-dioxoisoindolin-2-yl)-8-oxo-8-(quinolin-8-ylamino)oct-5-en-4-yl)hydrazine-1,2-dicarboxylate

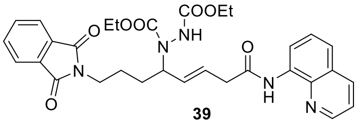

39

Compound **39** was isolated in 77 % yield (180.8 mg) with CuCl for 8 h. Flash silica gel chromatography (PE : EtOAc = 2:1).Yellow liquid. **^{1}H NMR** (400 MHz, Chloroform-*d*) δ 9.8 (s, 1H), 9.0 – 8.5 (m, 2H), 8.1 (dd, *J* = 8.3, 1.7 Hz, 1H), 7.7 (ddd, *J* = 49.7, 5.4, 3.0 Hz, 4H), 7.6 – 7.3 (m, 4H), 6.2 – 5.7 (m, 2H), 4.8 (s, 1H), 4.4 – 3.9 (m, 4H), 3.8 – 3.6 (m, 2H), 3.3 (dd, *J* = 6.9, 2.3 Hz, 2H), 2.2 – 1.6 (m, 4H), 1.5 – 0.7 (m, 6H). **^{13}C NMR** (101 MHz, Chloroform-*d*) δ 168.7, 168.4, 156.7, 155.9, 138.2, 136.6, 134.6, 133.9, 133.7, 128.0, 127.3, 126.4, 123.0, 121.8, 121.5, 116.7, 62.5, 61.6, 58.7, 41.9, 37.5, 28.8, 25.3, 14.5, 14.3. **HRMS**: calculated for $C_{31}H_{33}N_5O_7$ [M+H^{+}] 588.2453, found 588.2456.

Diethyl (*S*,*E*)-1-(5,9-dimethyl-1-oxo-1-(quinolin-8-ylamino)deca-3,8-dien-5-yl)hydrazine-1,2-dicarboxylate

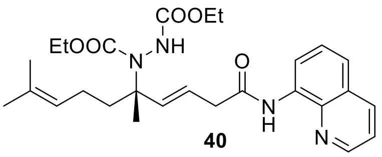

Compound **40** was isolated in 72 % yield (142.9 mg) with CuCl for 8 h. Flash silica gel chromatography (PE : EtOAc = 2:1). Colorless liquid. **^{1}H NMR** (400 MHz, Chloroform-*d*) δ 9.9 (d, *J* = 18.5 Hz, 1H), 8.9 – 8.7 (m, 2H), 8.3 – 8.1 (m, 1H), 7.6 – 7.4 (m, 3H), 7.0 – 6.5 (m, 1H), 6.3 – 6.1 (m, 1H), 5.9 (ddt, *J* = 23.2, 15.6, 7.2 Hz, 1H), 5.2 – 5.0 (m, 1H), 4.3 – 4.0 (m, 4H), 3.4 – 3.2 (m, 2H), 2.2 – 1.7 (m, 4H), 1.7 – 1.5 (m, 9H), 1.3 – 1.1 (m, 6H). **^{13}C NMR** (101 MHz, Chloroform-*d*) δ 169.7, 157.0, 148.2, 142.3, 138.5, 136.5, 134.4, 131.6, 128.0, 127.4, 124.2, 121.5, 120.4, 116.7, 64.8, 62.0, 42.0, 38.8, 38.3, 25.7, 23.6, 23.1, 17.6, 14.4. **HRMS**: calculated for $C_{27}H_{36}N_4O_5$ [M+H$^+$] 497.2758, found 497.2757.

Diethyl (*E*)-1-(7-oxo-1-((2-oxo-2H-chromen-6-yl)oxy)-7-(quinolin-8-ylamino)hept-4-en-3-yl)hydrazine-1,2-dicarboxylate

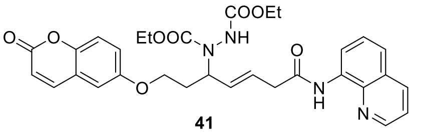

Compound **41** was isolated in 76 % yield (178.8 mg) with CuCl for 8 h. Flash silica gel chromatography (PE : EtOAc = 3:1). White solid, m.p. 104 – 106 °C. **^{1}H NMR** (400 MHz, Chloroform-*d*) δ 9.8 (s, 1H), 9.1 – 8.7 (m, 2H), 8.2 (dd, *J* = 8.3, 1.7 Hz, 1H), 7.6 – 7.4 (m, 4H), 7.4 – 7.3 (m, 1H), 6.9 – 6.7 (m, 2H), 6.2 (d, *J* = 9.5 Hz, 1H), 6.1 – 6.0 (m, 1H), 5.1 (s, 1H), 4.5 – 3.9 (m, 6H), 3.4 (d, *J* = 6.8 Hz, 2H), 2.4 – 2.1 (m, 2H), 1.3 – 1.1 (m, 6H). **^{13}C NMR** (101 MHz, Chloroform-*d*) δ 168.7, 162.2, 161.3, 155.8, 143.4, 138.2, 136.9, 133.8, 128.7, 128.1, 127.4, 122.1, 121.6, 117.0, 113.0, 112.5, 101.8, 65.4, 62.7, 62.0, 41.8, 14.4. **HRMS**: calculated for $C_{31}H_{32}N_4O_8$ [M+H$^+$] 589.2293, found 589.2287.

Diethyl 1-((*E*)-1-(4-((*E*)-3-(4-methoxyphenyl)-3-oxoprop-1-en-1-yl)phenyl)-5-oxo-5-(quinolin-8-ylamino)pent-2-en-1-yl)hydrazine-1,2-dicarboxylate

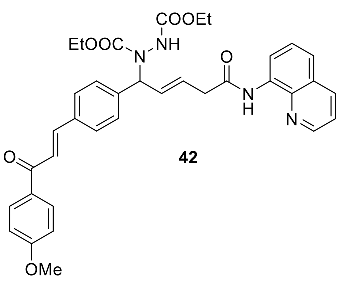

Compound **42** was isolated in 63 % yield (160.3 mg) with CuCl for 8 h. Flash silica gel chromatography (PE : EtOAc = 2:1).Yellow liquid. **^{1}H NMR** (400 MHz, Chloroform-*d*) δ 9.9 (s, 1H), 8.9 – 8.7 (m, 2H), 8.2 – 8.2 (m, 1H), 8.1 – 8.0 (m, 2H), 7.8 (d, *J* = 15.6 Hz, 1H), 7.7 – 7.3 (m, 9H), 7.0 (d, *J* = 8.8 Hz, 2H), 6.3 – 6.1 (m, 1H), 6.1 – 5.8 (m, 2H), 4.3 – 4.0 (m, 4H), 3.9 (s, 3H), 3.5 – 3.3 (m, 2H), 1.3 – 1.0 (m, 6H). **^{13}C NMR** (101 MHz, Chloroform-*d*) δ 188.7, 168.8, 163.5, 155.8, 148.5, 143.4, 138.3, 136.8, 134.6,

134.0, 132.6, 131.0, 130.8, 128.6, 128.5, 128.1, 127.4, 122.0, 122.0, 121.6, 116.9, 113.9, 62.8, 62.0, 55.5, 41.8, 14.5, 14.3. **HRMS**: calculated for $C_{36}H_{36}N_4O_7$ [M+H$^+$] 637.2657, found 637.2663.

Diethyl 1-((2*S*,*E*)-7-oxo-7-(quinolin-8-ylamino)-2-((3R,5S,7R,8R,9S,10S,12S,13R,14S,17R)-3,7,12-trimethoxy-10,13-dimethylhexadecahydro-1H-cyclopenta[a]phenanthren-17-yl)hept-4-en-3-yl)hydrazine-1,2-dicarboxylate

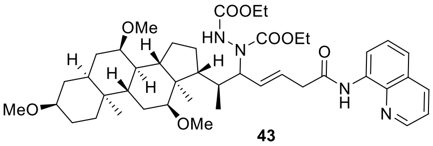

43

Compound **43** was isolated in 78 % yield (242.2 mg) with CuCl for 8 h. Flash silica gel chromatography (PE : EtOAc = 2:1). Colorless liquid. **^{1}H NMR** (400 MHz, Chloroform-*d*) δ 9.8 (s, 1H), 9.1 – 8.8 (m, 2H), 8.2 (d, *J* = 8.4 Hz, 1H), 7.6 – 7.4 (m, 4H), 6.2 – 5.8 (m, 2H), 4.7 (s, 1H), 4.3 – 4.0 (m, 4H), 3.3 (s, 6H), 3.2 – 3.1 (m, 7H), 3.0 – 3.0 (m, 1H), 2.1 – 1.4 (m, 17H), 1.3 – 1.2 (m, 8H), 1.1 (p, *J* = 5.3 Hz, 6H), 0.9 (s, 3H), 0.7 (s, 3H). **^{13}C NMR** (101 MHz, Chloroform-*d*) δ 169.0, 156.0, 149.1, 138.2, 136.9, 133.9, 128.4, 128.1, 127.3, 122.0, 121.5, 116.9, 81.9, 80.7, 63.9, 62.4, 61.5, 55.8, 55.5, 55.3, 46.3, 44.2, 42.6, 42.1, 41.9, 39.7, 39.6, 35.2, 34.8, 34.4, 27.9, 27.7, 26.7, 26.3, 23.2, 22.8, 21.9, 14.5, 14.4, 14.4, 12.8, 12.6. **HRMS**: calculated for $C_{44}H_{64}N_4O_8$ [M+H$^+$] 777.4797, found 777.4793.

Diethyl-1-((*E*)-1-(((8R,9S,13S,14S)-13-methyl-17-oxo-7,8,9,11,12,13,14,15,16,17-decahydro-6H-cyclopenta[a]phenanthren-3-yl)oxy)-8-oxo-8-(quinolin-8-ylamino)oct-5-en-4-yl)hydrazine-1,2-dicarboxylate

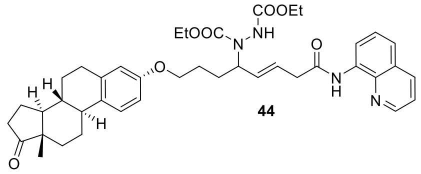

44

Compound **44** was isolated in 81 % yield (230.1 mg) with CuCl for 8 h. Flash silica gel chromatography (PE : EtOAc = 2:1). Colorless liquid. **^{1}H NMR** (400 MHz, Chloroform-*d*) δ 9.8 (s, 1H), 9.1 – 8.6 (m, 2H), 8.3 – 8.0 (m, 1H), 7.6 – 7.3 (m, 4H), 7.2 – 7.0 (m, 1H), 6.8 – 6.5 (m, 2H), 6.1 – 5.8 (m, 2H), 4.8 (s, 1H), 4.3 – 3.9 (m, 6H), 3.3 (d, *J* = 6.9 Hz, 2H), 2.9 – 2.7 (m, 2H), 2.5 – 2.3 (m, 2H), 2.3 – 2.1 (m, 2H), 2.0 – 1.8 (m, 6H), 1.7 – 1.4 (m, 6H), 1.3 – 1.1 (m, 7H), 0.9 (s, 3H). **^{13}C NMR** (101 MHz, Chloroform-d) δ 221.0, 168.9, 157.0, 156.0, 148.9, 138.3, 137.7, 136.8, 134.7, 134.0, 131.9, 128.1, 127.4, 126.3, 122.0, 121.6, 116.8, 114.5, 112.1, 67.5, 62.6, 61.8, 59.1, 50.4, 48.0, 44.0, 41.9, 38.4, 35.9, 31.6, 29.6, 28.1, 26.6, 26.1, 25.9, 21.6, 14.5, 14.4, 13.9. **HRMS**: calculated for $C_{41}H_{50}N_4O_7$ [M+H$^+$] 711.3752, found 711.3752.

Diethyl-1-((*E*)-1-(((3S,5S,8R,9S,10S,13R,14S,17R)-10,13-dimethyl-17-((R)-5-methylhexan-2-yl)hexadecahydro-1H-cyclopenta[a]phenanthren-3-yl)oxy)-1,7-dioxo-7-(quinolin-8-ylamino)hept-4-en-3-yl)hydrazine-1,2-dicarboxylate

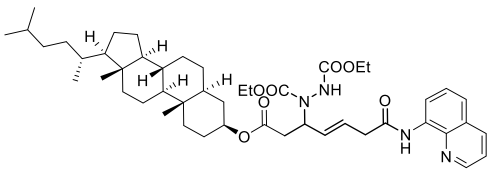

45

Compound **45** was isolated in 82 % yield (267.1 mg) with CuCl for 8 h. Flash silica gel chromatography (PE : EtOAc = 2:1). Yellow liquid. **^{1}H NMR** (400 MHz, Chloroform-*d*) δ 9.8 (s, 1H), 9.0 – 8.7 (m, 2H), 8.2 (dd, *J* = 8.2, 1.6 Hz, 1H), 7.6 – 7.4 (m, 4H), 6.1 – 5.8 (m, 2H), 5.2 (s, 1H), 4.8 – 4.6 (m, 1H), 4.3 – 4.0 (m, 4H), 3.3 (d, *J* = 6.7 Hz, 2H), 3.0 – 2.6 (m, 2H), 2.1 – 1.4 (m, 12H), 1.4 – 0.9 (m, 28H), 0.9 – 0.8 (m, 10H), 0.8 (s, 3H), 0.6 (s, 3H). **^{13}C NMR** (101 MHz, Chloroform-*d*) δ 170.3, 168.7, 156.9, 155.5, 148.7, 138.3, 136.7, 134.1, 133.3, 128.1, 127.4, 126.1, 121.9, 121.6, 116.8, 74.2, 62.7, 61.9, 56.4, 56.3, 54.2, 44.6, 44.6, 42.6, 41.8, 40.0, 39.5, 37.0, 36.7, 36.2, 35.8, 35.5, 35.4, 33.9, 32.0, 28.6, 28.2, 28.0, 27.4, 24.2, 23.8, 22.8, 22.6, 21.2, 18.7, 14.5, 14.4, 14.4, 12.2, 12.1. **HRMS**: calculated for $C_{48}H_{70}N_4O_7$ [M+H$^+$] 815.5317, found 815.5314.

Diethyl 1-((3*E*,15*Z*)-1-oxo-1-(quinolin-8-ylamino)tetracosa-3,15-dien-5-yl)hydrazine-1,2-dicarboxylate

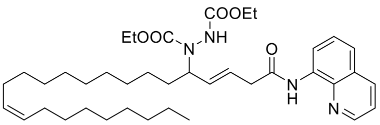

46

Compound **46** was isolated in 76 % yield (202.0 mg) with CuCl for 8 h. Flash silica gel chromatography (PE : EtOAc = 2:1). Colorless liquid. **^{1}H NMR** (400 MHz, Chloroform-*d*) δ 9.8 (s, 1H), 9.1 – 8.6 (m, 2H), 8.2 (dd, *J* = 8.3, 1.7 Hz, 1H), 7.7 – 7.3 (m, 4H), 6.2 – 5.6 (m, 2H), 5.5 – 5.1 (m, 2H), 4.7 (s, 1H), 4.3 – 3.8 (m, 4H), 3.5 – 3.1 (m, 2H), 2.2 – 1.9 (m, 4H), 1.9 – 1.6 (m, 2H), 1.5 – 1.0 (m, 34H), 1.0 – 0.6 (m, 3H). **^{13}C NMR** (101 MHz, Chloroform-*d*) δ 169.0, 156.8, 156.0, 148.8, 138.4, 136.8, 135.2, 134.1, 129.9, 128.1, 127.4, 121.9, 121.6, 116.8, 62.5, 61.8, 59.3, 42.0, 31.9, 31.5, 29.8, 29.8, 29.7, 29.7, 29.6, 29.6, 29.6, 29.5, 29.5, 29.3, 29.3, 27.2, 26.2, 22.7, 14.5, 14.4, 14.1. **HRMS**: calculated for $C_{39}H_{60}N_4O_5$ [M+H$^+$] 665.4636, found 665.4637.

5. Synthetic utilities

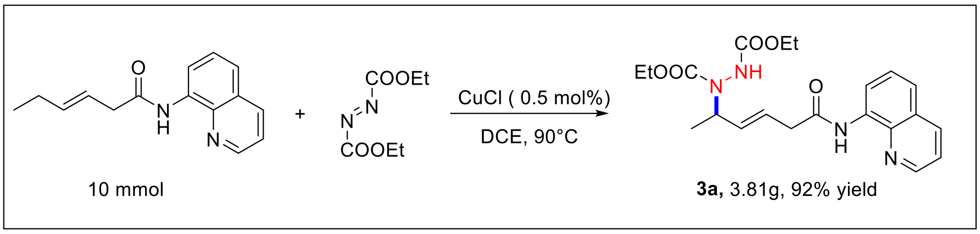

A mixture of amide (10 mmol, 1.0 equiv), CuCl (0.05 mmol, 0.005 equiv), and azodicarboxylate (20 mmol, 2.0 equiv) in DCE (20 mL) in a 50 mL glass vial (sealed with PTFE cap) was heated at 90 °C for indicated time. The reaction progress was monitored by thin layer chromatography. Upon completion, the crude mixture was concentrated under reduced pressure and

the residue was purified by silica gel column chromatography (PE : EtOAc = 2:1) to afford **3a** (3.81 g, 92% yield).

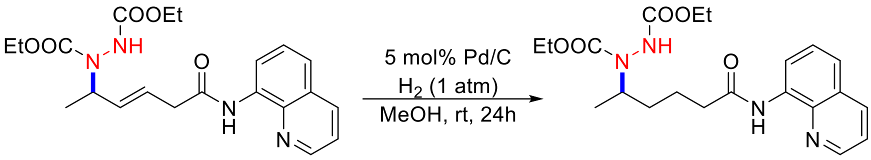

A 10 mL tube equipped with a magnetic stirring bar was charged with Pd/C (2.6 mg, 5 wt%, 10.0 mol%) and **3aa** (82.8 mg, 0.2 mmol, 1.0 eq) in MeOH (5.0 mL). The reaction mixture was flushed with H_2 (3x) and stirred at rt with a H_2 balloon for 24 hours. After cooling down, the reaction mixture was filtered through a short pad of silica gel eluting with dichloromethane and concentrated. The solvent was removed under reduced pressure and the residue was purified by silica gel column chromatography (PE : EtOAc = 2:1) to obtain **47** (74.5 mg, 90% yield).

Diethyl-1-(6-oxo-6-(quinolin-8-ylamino)hexan-2-yl)hydrazine-1,2-dicarboxylate

Colorless liquid. **^{1}H NMR** (400 MHz, Chloroform-d) δ 9.8 (s, 1H), 9.0 – 8.6 (m, 2H), 8.2 (dd, J = 8.3, 1.7 Hz, 1H), 7.6 – 7.4 (m, 3H), 7.1 (d, J = 82.6 Hz, 1H), 4.5 – 4.0 (m, 5H), 2.8 – 2.5 (m, 2H), 2.0 – 1.4 (m, 4H), 1.3 – 1.1 (m, 9H). **^{13}C NMR** (101 MHz, Chloroform-d) δ 171.5, 157.2, 156.2, 148.2, 138.2, 136.4, 134.3, 127.9, 127.3, 121.6, 121.5, 116.6, 62.2, 61.7, 53.1, 41.7, 37.3, 33.2, 22.0, 18.2, 14.5, 14.4. **HRMS**: calculated for $C_{21}H_{28}N_4O_5$ [M+H$^+$] 417.2132, found 417.2135.

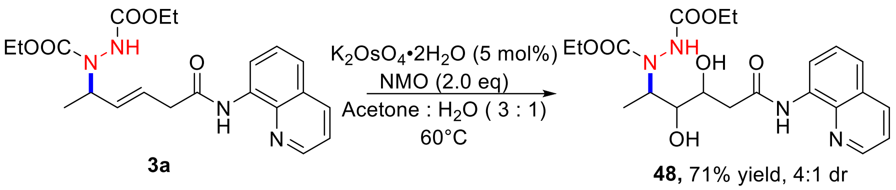

To a solution of **3a** (82.8 mg, 0.2 mmol, 1.0 eq) in Acetone/H_2O (2.0 mL, 3:1) was added K_2OsO_4·$2H_2O$ (3.68 mg, 0.01 mmol, 0.05 eq) and NMO (46.8 mg, 0.4 mmol, 2.0 eq) at 60 °C. The reaction progress was monitored by thin layer chromatography. Upon completion, the reaction mixture was washed by NaCl saturated solution (10 mL x 3). The organic phase was dried by anhydrous Na_2SO_4. The solvent was removed under reduced pressure and the residue was purified by flash column chromatography (PE : EtOAc = 1:2) to obtain **48** (63.6 mg, 71%).

Diethyl 1-(3,4-dihydroxy-6-oxo-6-(quinolin-8-ylamino)hexan-2-yl)hydrazine-1,2-dicarboxylate

Yellow liquid. **^{1}H NMR** (400 MHz, Chloroform-*d*) δ 10.4 – 9.9 (m, 1H), 8.9 – 8.5 (m, 2H), 8.2 – 8.0 (m, 1H), 7.5 – 7.4 (m, 3H), 7.1 (d, *J* = 25.0 Hz, 1H), 5.3 – 3.6 (m, 8H), 3.5 – 3.2 (m, 1H), 3.2 – 2.7 (m, 2H), 1.3 – 1.0 (m, 9H). **^{13}C NMR** (101 MHz, Chloroform-*d*) δ 171.2, 156.9, 155.8, 148.3, 138.4, 136.2, 134.3, 127.9, 127.2, 121.6, 116.9, 116.7, 82.1, 68.1, 62.8, 60.4, 54.2, 40.1, 21.0, 14.4, 14.2. **HRMS**: calculated for $C_{21}H_{28}N_4O_7$ [M+H$^+$] 449.2031, found 449.2038.

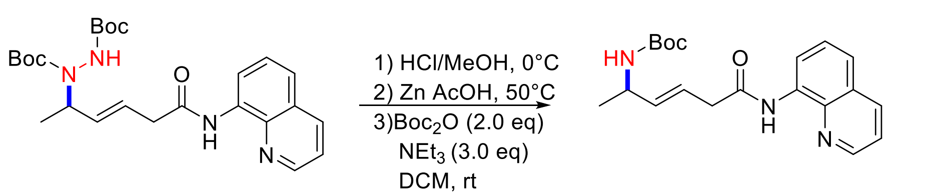

5 **49**, 67% yield

The **5** (188 mg, 0.4 mmol, 1.0 equiv.) was dissolved in CH_3OH (2 mL) in a round bottom flask, to which HCl (2 M in CH_3OH, 0.5 mL) was slowly added at 0 ˚C. The reaction was then sealed with a PTFE septum and stirred at room temperature until the consumption of compound **5**. After the solvent was removed under reduced pressure, the residue was washed with anhydrous ether (1 mL × 2) and then dried in vacuo to afford the corresponding hydrazine hydrochloride. Then the hydrazine hydrochloride was dissolved in 2 mL of acetone, acetic acid (3 mL) and freshly activated Zn dust (130 mg, 2 mmol) were added. After 2 h, the reaction was filtered through Celite and washed with MeOH (4 mL). The mixture was acidified with 37% HCl (pH = 1), and the solvents were removed under reduced pressure. The residue was then dissolved in HCl (1 M, 20 mL) and washed with Et_2O (3 × 15 mL). The pH of the aqueous phase was adjusted to 12 with NaOH pellets and the resulting solution was saturated with NaCl and extracted with Et_2O (3 × 20 mL). The combined organic layers were dried over Na_2SO_4, filtered and concentrated in vacuo to afford the crude amine. The crude product was then dissolved in CH_2Cl_2, NEt_3 (55 µL, 0.4 mmol) and $(Boc)_2O$ (87.3 mg, 0.4 mmol) was added to the solution and stirred at room temperature for 5 h. Compound **49** was then isolated through a silica gel flash column (PE : EtOAc = 3:1) as colorless oil (95.1 mg, 67% yield, over 3 steps).

Tert-butyl (*E*)-(6-oxo-6-(quinolin-8-ylamino)hex-3-en-2-yl)carbamate

Yellow liquid. **^{1}H NMR** (500 MHz, Chloroform-*d*) δ 10.0 (s, 1H), 8.9 – 8.7 (m, 2H), 8.2 (dd, *J* = 8.2, 1.7 Hz, 1H), 7.7 – 7.3 (m, 3H), 7.0 (s, 1H), 6.1 – 5.8 (m, 1H), 5.8 – 5.6 (m, 1H), 3.8 – 3.6 (m, 1H), 3.4 – 3.0 (m, 2H), 1.6 – 1.0 (m, 12H). **^{13}C NMR** (126 MHz, Chloroform-*d*) δ 169.2, 156.9, 148.4, 139.0, 138.5, 136.6, 134.2, 128.1, 127.4, 125.4, 121.8, 121.7, 121.6, 116.7, 80.2, 57.7, 42.0,

28.2, 18.9. **HRMS**: calculated for $C_{20}H_{25}N_3O_3$ [M+H$^+$] 356.1969, found 356.1968.

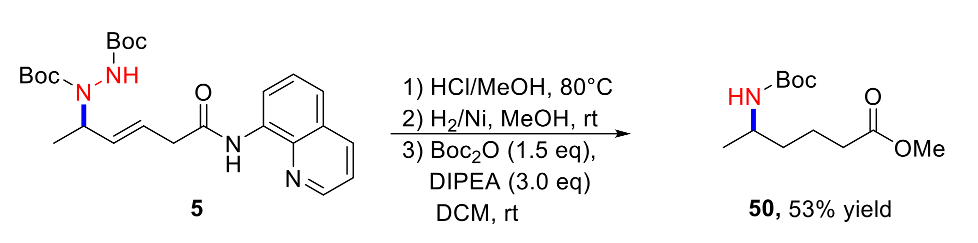

The **5** (188 mg, 0.4 mmol, 1.0 equiv.) was dissolved in HCl/MeOH (2.0 mL) solution at 80 °C. After 5 hours, the solvent was removed in vacuo and the crude product was dissolved in MeOH (4.0 mL). The reaction solution was treat with Raney-Ni (400 mg) and stirred under H_2 (3 atm) atmosphere for 24 h. The reaction mixture was filtered over celite and concentrated to give crude amino. The crude amino was dissolved in DCM (4.0 mL). DIPEA (200 µL, 0.12 mmol, 3 equiv.) and Boc_2O (104 µL, 0.44 mmol, 1.1 equiv.) were added. After the reaction mixture was stirred overnight. The solvent was removed in vacuo and the crude product was directly loaded onto silica gel column then isolated through a silica gel flash column (PE : EtOAc = 4:1) as colorless oil **50** (51.9 mg, 53% yield, over 3 steps).

Methyl 5-((tert-butoxycarbonyl)amino)hexanoate

Colorless liquid. **^{1}H NMR** (400 MHz, Chloroform-*d*) δ 4.4 (s, 1H), 3.7 (s, 4H), 2.4 – 2.3 (m, 2H), 1.7 – 1.6 (m, 2H), 1.4 (s, 11H), 1.1 (d, J = 6.6 Hz, 3H). ^{13}C NMR (101 MHz, Chloroform-*d*) δ 174.0, 155.5, 79.1, 51.6, 46.2, 36.7, 33.8, 28.5, 21.5, 21.3. **HRMS**: calculated for $C_{12}H_{23}NO_4$ [M+Na$^+$] 268.1519, found 268.1522.

6. Primary kinetic isotope effects

a) Preparation of deuterated substrate 55-d_3

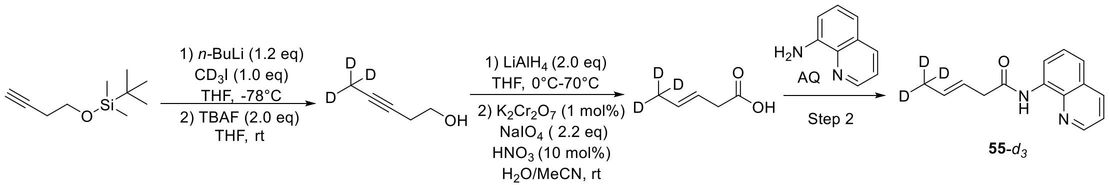

To a dry 250 mL round-bottom flask were added (but-3-yn-1-yloxy)(tert-butyl)-dimethylsilane (6.36 g, 35.0 mmol) and THF (100 mL). The solution was cooled in a dry ice bath to −78 °C. To this was added *n*-BuLi (16.6 mL, 38.0 mmol, 1.2 equiv), and the reaction was allowed to stir for 2 h. Iodomethane-d_3 (5.00 g, 34.5 mmol) in THF (10 mL) was added to the mixture, which was stirred overnight with warming to room temperature. The reaction was quenched with water (10 mL). The layers were separated, and the aqueous layer was extracted with diethyl ether (3 × 10 mL). The

combined organic layers were washed with brine (3 × 10 mL), dried over magnesium sulfate, and concentrated under reduced pressure. The product was purified using flash chromatography and recovered in a 79% yield.

The protected alcohol, tertbutyldimethyl(pent-3-yn-5-d_3-1-yloxy)silane (5.54 g, 27.4 mmol), was deprotected using 1 M tetra-n-butylammonium fluoride (TBAF) (14.3 mL, 54.8 mmol, 2 equiv) in THF (50 mL). The reaction was allowed to stir overnight and was quenched with water (15 mL). The layers were separated, and the aqueous layer was extracted with diethyl ether (3 × 10 mL). The combined organic layers were washed with brine (3 × 10 mL), dried over magnesium sulfate, and concentrated under reduced pressure. The alcohol was carried on without purification. To reduce the alkyne to the alkene and oxidizing alcohols into carboxylic acid can refer to the post sequence synthesis method of **57**-d_2. The next step is general procedure (step 2) for the preparation of amide substrates **55**-d_3.

Supplementary Figure 1 ^{1}H NMR-spectrum (400 MHz, $CDCl_3$) of **55**-d_3

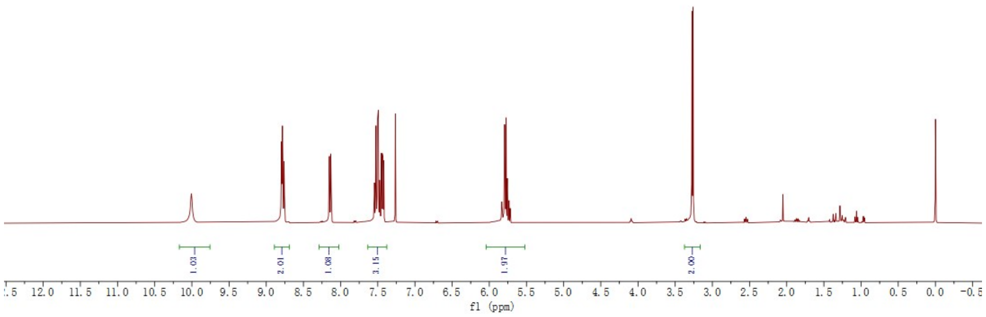

Substrate **55**-d_3 was treated in standard conditions to give **58**-d_2

Supplementary Figure 2 ^{1}H NMR-spectrum (400 MHz, $CDCl_3$) of **58**-d_2

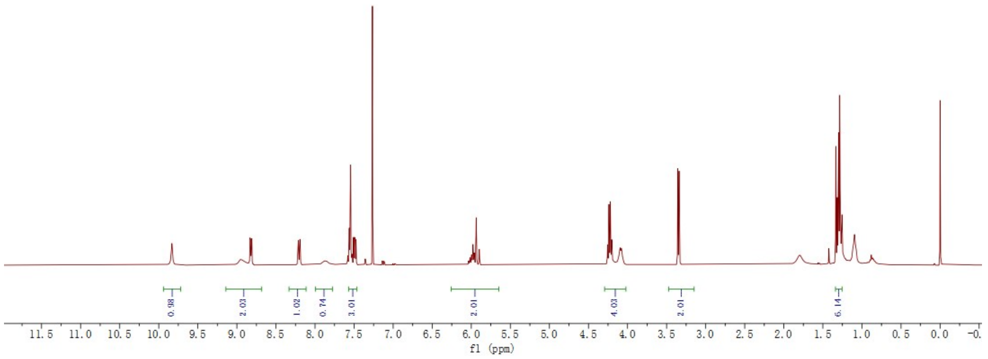

b) Preparation of deuterated substrate 56-d_2

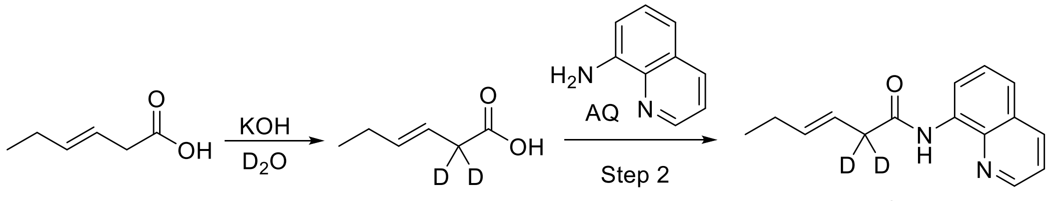

56-d_2

(*E*)-2,2-dideutero-hex-3-enoic acid: To a 10 ml solution of 5 M KOH dissolved in D_2O, (E)-hex-3-enoic acid was added (400 mg). The solution was set to stir at 50 ºC for 1 h, after which the solvent was removed in vacuo. An additional 5 ml of D_2O was added to solution, which was again set to stir at 50 ºC for 1 h, and the solvent was removed in vacuo. The process of adding fresh D_2O followed by removal in vacuo was repeated a total of four times. The solution was then acidified with 10 M HCl to a pH of approximately 2, and extracted three times with EtOAc. Solvent was removed in vacuo, to afford (*E*)-2,2-dideutero-hex-3-enoic acid (390 mg, 98%). The next step is general procedure (step 2) for the preparation of amide substrates **56**-d_2 (^{1}H NMR).

Supplementary Figure 3 ^{1}H NMR-spectrum (400 MHz, $CDCl_3$) of **56**-d_2

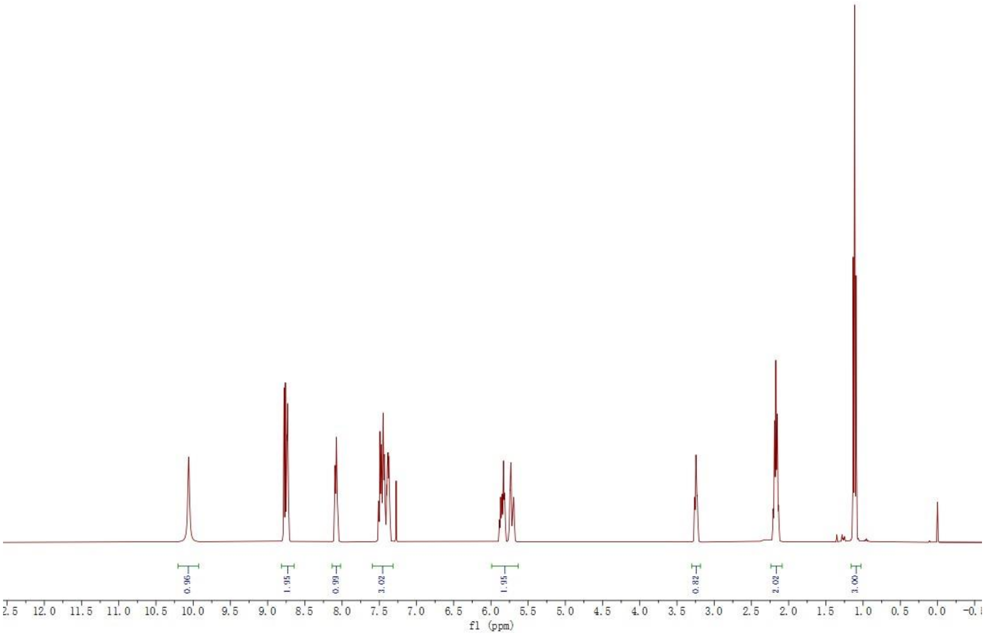

Substrate **56**-*d*$_2$ was treated in standard conditions to give **59**-*d*$_2$

Supplementary Figure 4 ^{1}**H NMR**-spectrum (400 MHz, $CDCl_3$) of **59**-*d*$_2$

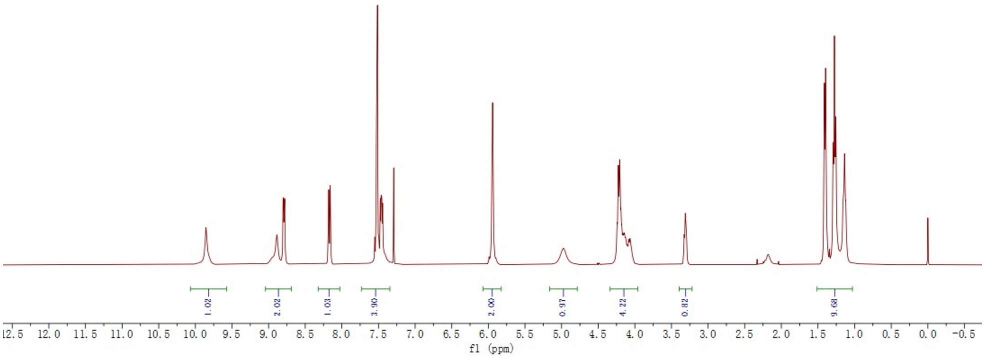

c) Preparation of deuterated substrate 57-*d*$_2$

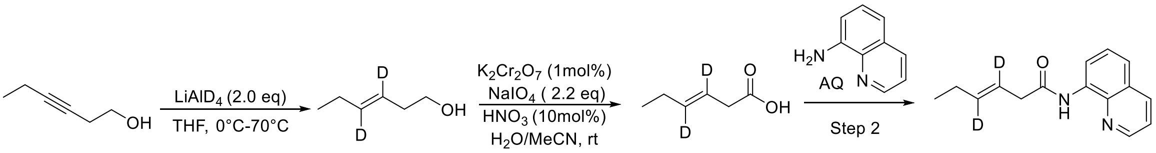

57-*d*$_2$

(*E*)-hex-3-en-3,4-*d*$_2$-1-ol: To a dry 2-necked, 250 mL roundbottom flask equipped with a stir

bar was added lithium aluminum deuteride (10.2 mmol, 2 equiv). The flask was fitted with an addition funnel and a reflux condenser. The apparatus was purged with nitrogen and dry THF (50 mL, 0.1 M) was added. The solution was then cooled to 0 °C while stirring. To the closed addition funnel under nitrogen was added 3-hexyn-1-ol (5.1 mmol, 1 equiv) and THF (10 mL, 0.5 M). The addition funnel was then opened and washed with THF (5 mL). The reaction was allowed to warm to room temperature over 1 h. The reaction was then heated to 70 °C for 72 h. The reaction was cooled in an ice bath and then quenched by addition of D_2O (10 mL). Et_2O (10 mL) was added and the layers were separated. The aqueous layer was extracted with Et_2O (3 × 10 mL). The organic solution was washed with brine (10 mL) and dried over Na_2SO_4. After filtration, the solvent was removed *in vacuo* to afford the crude material that was used directly in the next step.

To a solution of $K_2Cr_2O_7$ (6 mg, 0.02 mmol, 1 mol%), 65% aq. HNO_3 (39 mg, 0.2 mmol, 10 mol%) and $NaIO_4$ (941 mg, 4.4 mmol, 2.2 eq.) in H_2O (2.15 mL) were subsequently at 0 °C added 4 mL of MeCN and 2.36 mL of (*E*)-3-en-1-ol (2.0 g, 2 mmol, 1.0 eq.). The reaction mixture was stirred at 0 °C for 8 h and at 10 °C overnight (TLC conversion 98%). Inorganic salts were filtrated off and washed with Et_2O. The organic phase was separated and the aqueous phase was extracted with Et_2O (3×10 mL). The combined organic phases were dried (Na_2SO_4) and evaporated. The crude product was purified by flash chromatography. The next step is general procedure (step 2) for the preparation of amide substrates **57**-*d*2. (^{1}H NMR)

Supplementary Figure 5 ^{1}H NMR-spectrum (400 MHz, $CDCl_3$) of **57**-*d*2

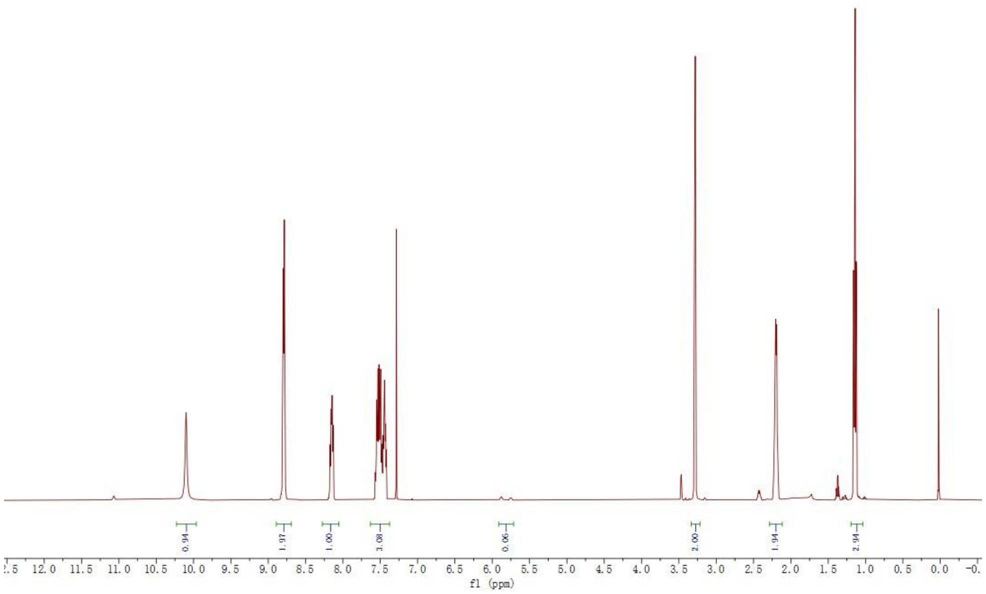

Substrate **57**-*d*2 was treated in standard conditions to give **60**-*d*2

Supplementary Figure 6 ^{1}H NMR-spectrum (400 MHz, $CDCl_3$) of **60**-*d*2

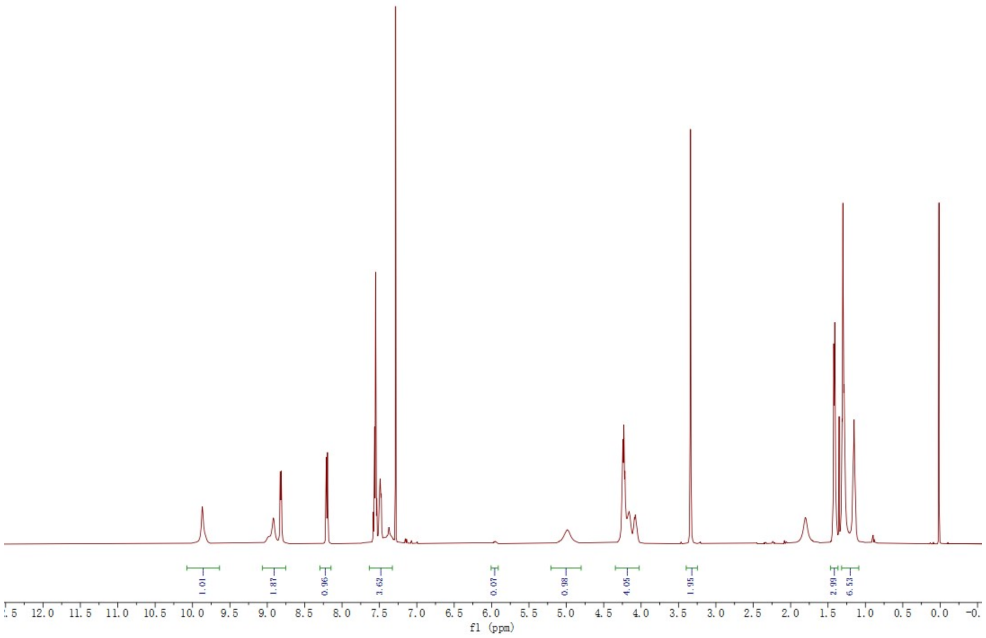

7. General Procedure for KIE Experiments

a) Parellel kinetic isotope effect

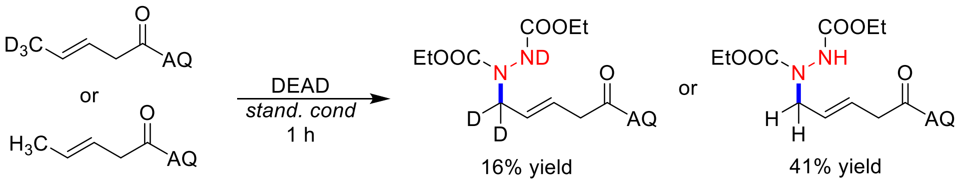

One mixture of trans-3-pentenamide (0.4 mmol, 96 mg), CuCl (0.004 mmol, 0.01 equiv), and azodicarboxylate (0.8 mmol, 2 equiv) in DCE (2 mL) in a 10 mL glass vial (sealed with PTFE cap). The other mixture of amide **55**-d_3 (0.4 mmol, 96 mg), CuCl (0.004 mmol, 0.01 equiv), and azodicarboxylate (0.8 mmol, 2 equiv) in DCE (2 mL) in a 10 mL glass vial (sealed with PTFE cap). After heated at 90 °C for 1 h and cooled to room temperature, the reaction mixtures were diluted with DCM, filtered through a pad of Celite and concentrated *in vacuo*. The resulting residues were purified by flash column chromatography to give the amidated product **11** as a colorless oil. (65.6 mg, 41% yield) and the amidated product **58**-d_2 as a colorless oil. (25.7 mg, 16% yield) respectively. By the yield of the product, KIE=41%/16%=2.5625.

b) Intermoleculr kinetic isotope effect

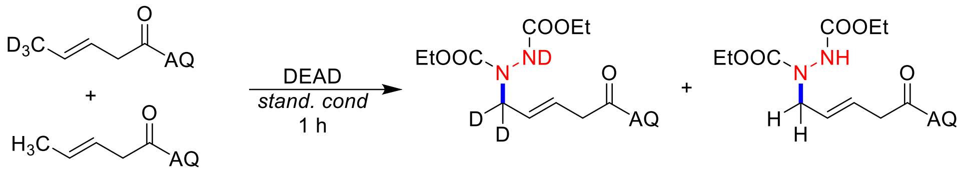

KIE = 2.7

A mixture of trans-3-pentenamide (0.4 mmol, 96 mg), amide **55-d_3** (0.4 mmol, 96 mg), CuCl (0.008 mmol, 0.01 equiv), and azodicarboxylate (1.6 mmol, 2 equiv) in DCE (4 mL) in a 20 mL glass vial (sealed with PTFE cap). After heated at 90 °C for 1 h and cooled to room temperature, the reaction mixtures were diluted with DCM, filtered through a pad of Celite and concentrated *in vacuo*. The resulting residue was purified by flash column chromatography to give the amidated product **11/58-d_2** as a colorless oil (23 mg). By the analysis of the ^{1}H NMR of **11/58-d_2**, KIE=1.46/0.54=2.7037.

Supplementary Figure 7 ^{1}H NMR-spectrum (400 MHz, $CDCl_3$) of **11/58-d_2**

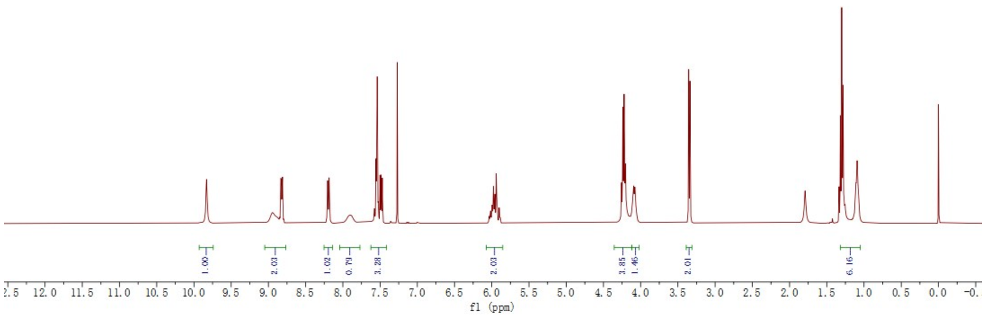

8. Crystal structure information for compound 3a

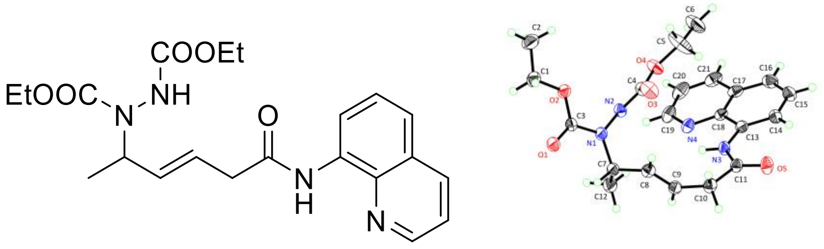

X-ray structure of **3a** (CCDC 215143)

A colorless block shaped crystal of **3a** ($C_{21}H_{26}N_4O_5$) was used for the X-ray crystallographic analysis. The X-ray intensity data were measured at 296(2) K, on a Bruker D8 VENTURE CMOS photon 100 diffractometer with helios mx multilayer monochrmator Cu Kα radiation (λ = 1.54178 Å).

Supplementary Table 2. Crystal Data and Structure Refinement for **3a**.

Empirical formula	$C_{21}H_{26}N_4O_5$
Formula weight	414.19
Temperature	296(2) K
Wavelength	1.54178 A
Crystal system, space group	Triclinic, P-1
Unit cell dimensions	a = 8.2565(19) A alpha = 85.680 (14) deg.<br>b = 11.307(2) A beta = 84.82(2)deg.<br>c = 11.622(3) A gamma = 88.29 (2) deg.
Volume	1077.2(4) A^3
Z, Calculated density	2, 1.275 Mg/m^3
Absorption coefficient	0.764mm^-1
F(000)	438
Crystal size	0.180 x 0.160 x 0.150mm
Theta range for data collection	3.921 to 68.529 deg.
Limiting indices	-9<=h<=9, -13<=k<=13, -14<=l<=14
Reflections collected / unique	18842 / 3927 [R(int) = 0.0352]
Completeness to theta =63.754	99.1 %
Refinement method	Full-matrix least-squares on F^2
Data / restraints / parameters	3927 / 0 / 277
Goodness-of-fit on F^2	1.051
Final R indices [I>2sigma(I)]	R_1 = 0.0438, wR_2 = 0.1235
R indices (all data)	R_1 = 0.0524, wR_2 = 0.1313
Extinction coefficient	n/a
Largest diff. peak and hole	0.248 and -0.217 e.A^-3

9. Computational details

The UM06 density functional[4] was employed for the computational study. The Lanl2DZ basis set together with the Lanl2DZ pseudopotential[5,6] was used to describe Cu atoms and 6-31g(d,p) basis set[7,8] was utilized for other atoms. Vibrational frequency analyses were carried out at the same level to confirm all the optimized structures as transition states (only one imaginary frequency), and provided the thermal relative Gibbs free energy correction. The solvent effect of dichloroethane in

the reaction was evaluated using the SMD solvation model developed by Truhlar and Cramer[9]. This model was used for single point energy calculations based on the gas phase optimized geometries with M06 at a larger basis set (SDD[10,11] for Cu atoms and 6-311+g(d,p) for other atoms). The wavefunction stability of all the computed results were checked. For the purpose of discussion, the solvation Gibbs free energy was used and it was obtained from the addition of solvation single point energy and gas-phase thermal correction to Gibbs free energy. All calculations were carried out by Gaussian 16 program package[12].

10 Supplementary Figures

NMR Spectra

Supplementary Figure 8 ^{1}H NMR-spectrum (400 MHz, $CDCl_3$) of **4**

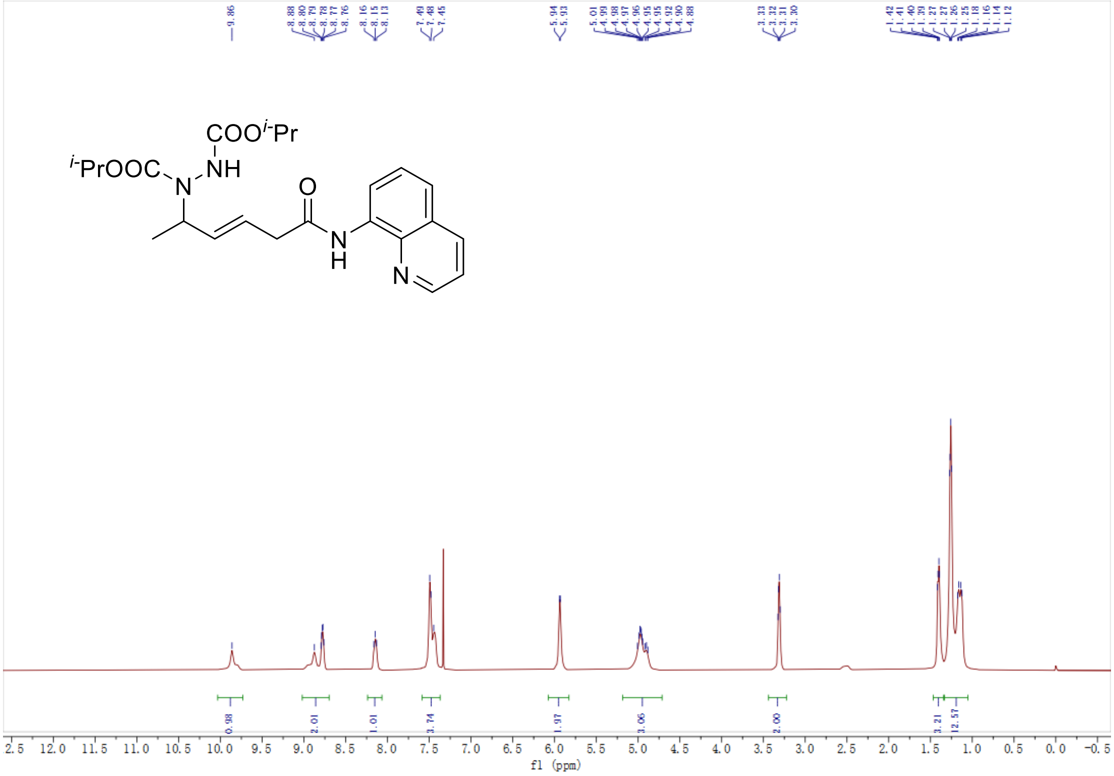

Supplementary Figure 9 ^{13}C NMR-spectrum (101 MHz, $CDCl_3$) of **4**

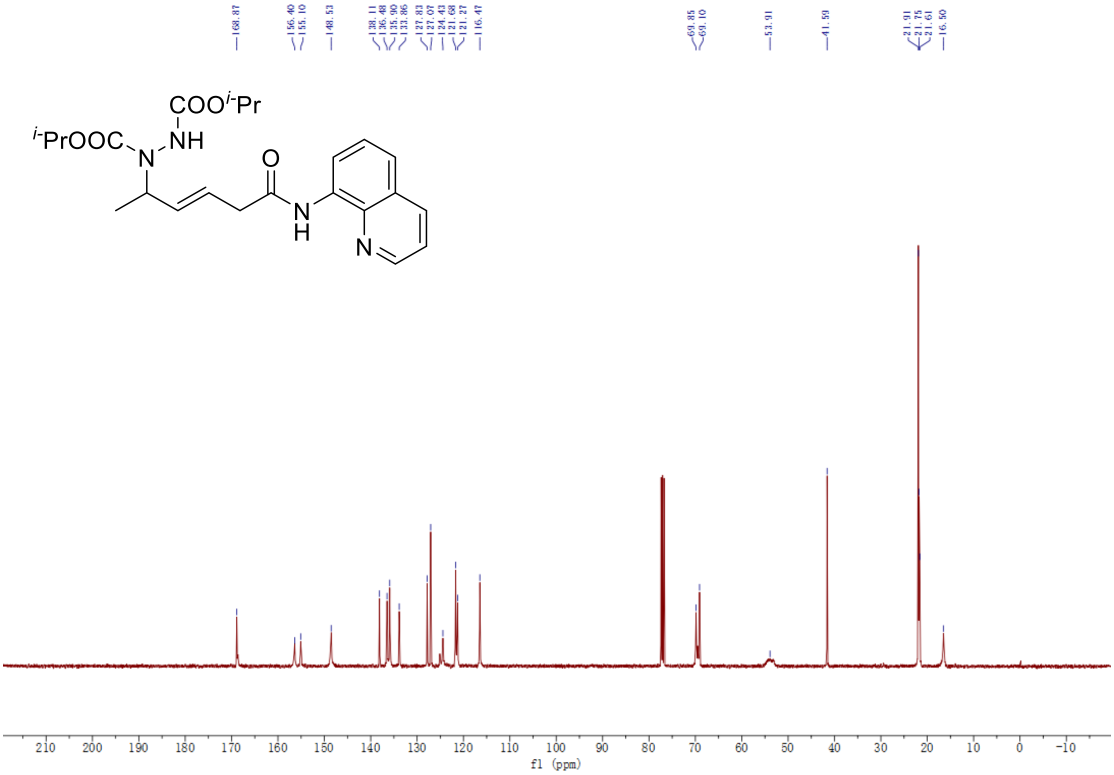

Supplementary Figure 10 ^{1}**H NMR**-spectrum (400 MHz, $CDCl_3$) of **5**

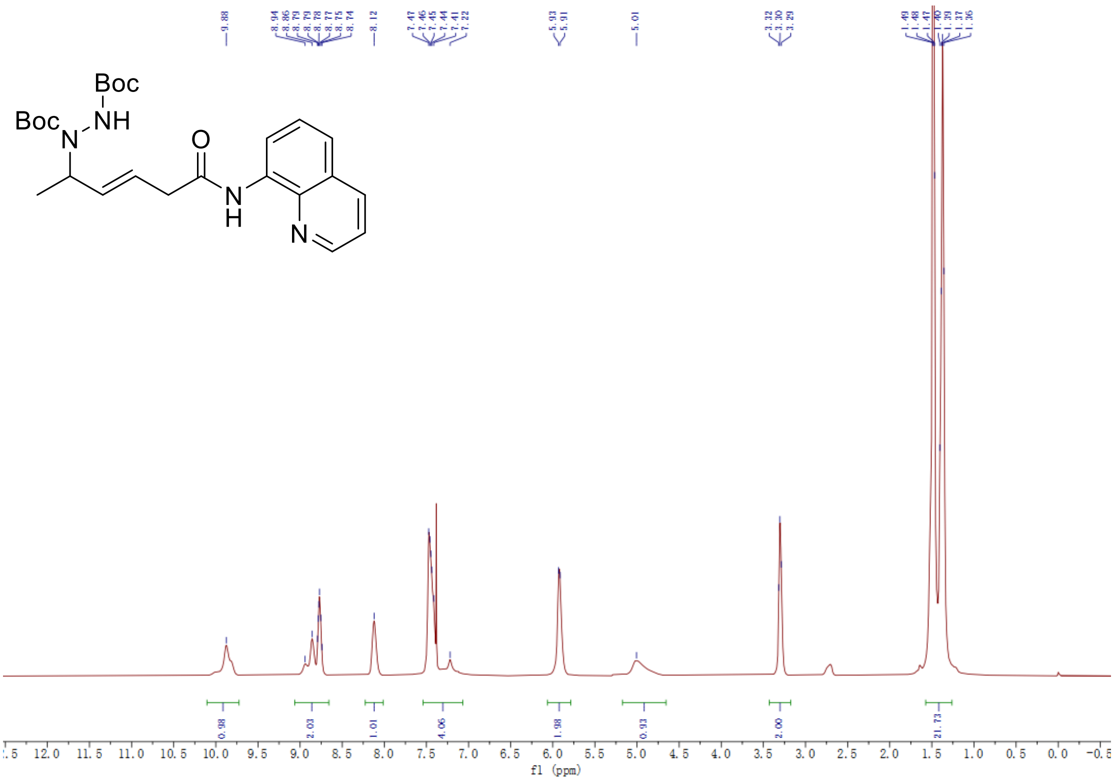

Supplementary Figure 11 ^{13}**C NMR**-spectrum (101 MHz, $CDCl_3$) of **5**

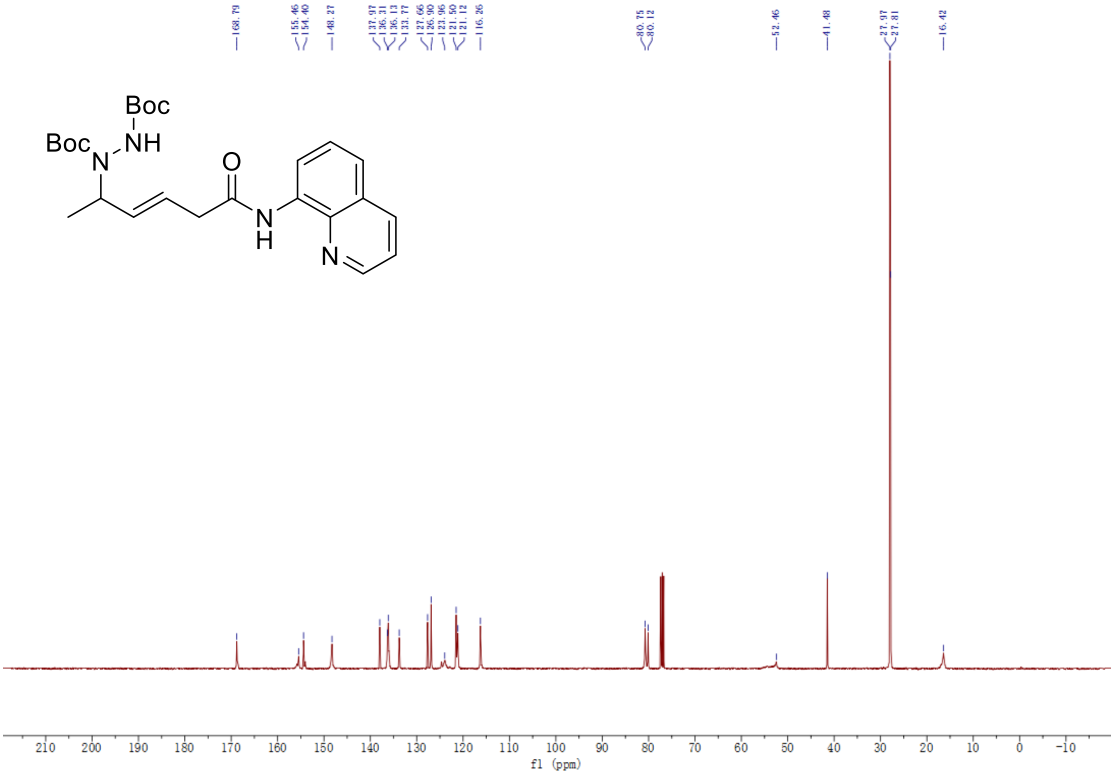

Supplementary Figure 12 ^{1}**H NMR**-spectrum (400 MHz, $CDCl_3$) of **6**

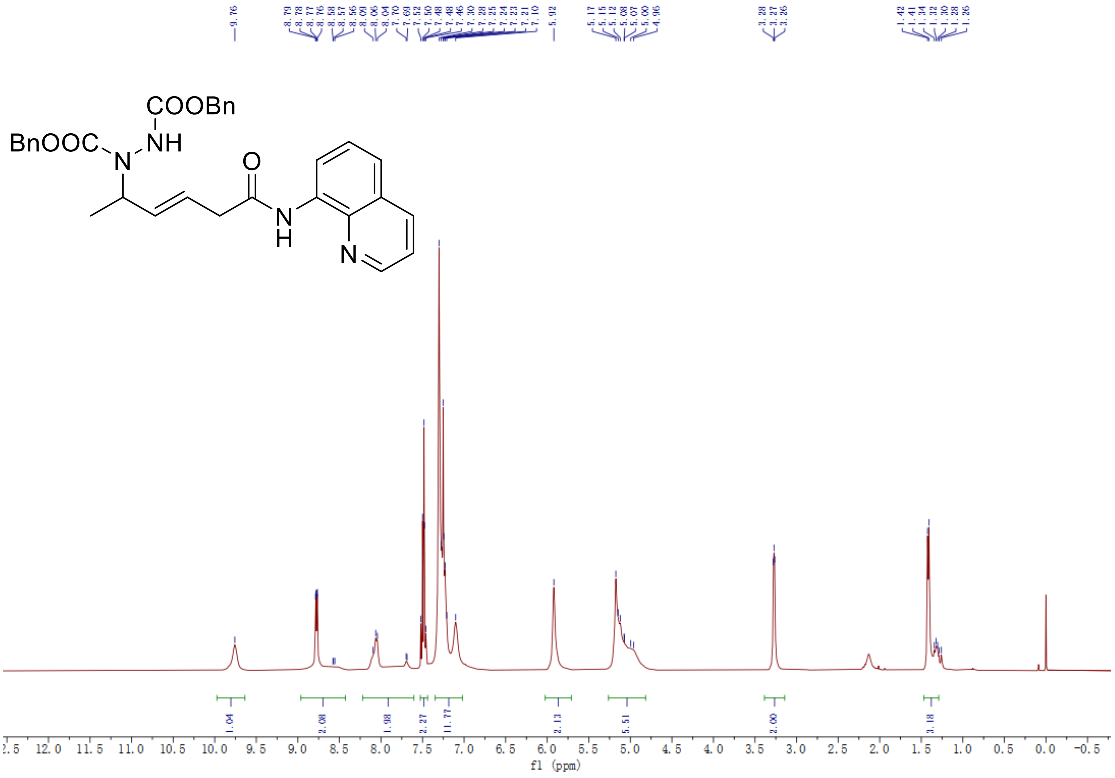

Supplementary Figure 13 ^{13}**C NMR**-spectrum (101 MHz, $CDCl_3$) of **6**

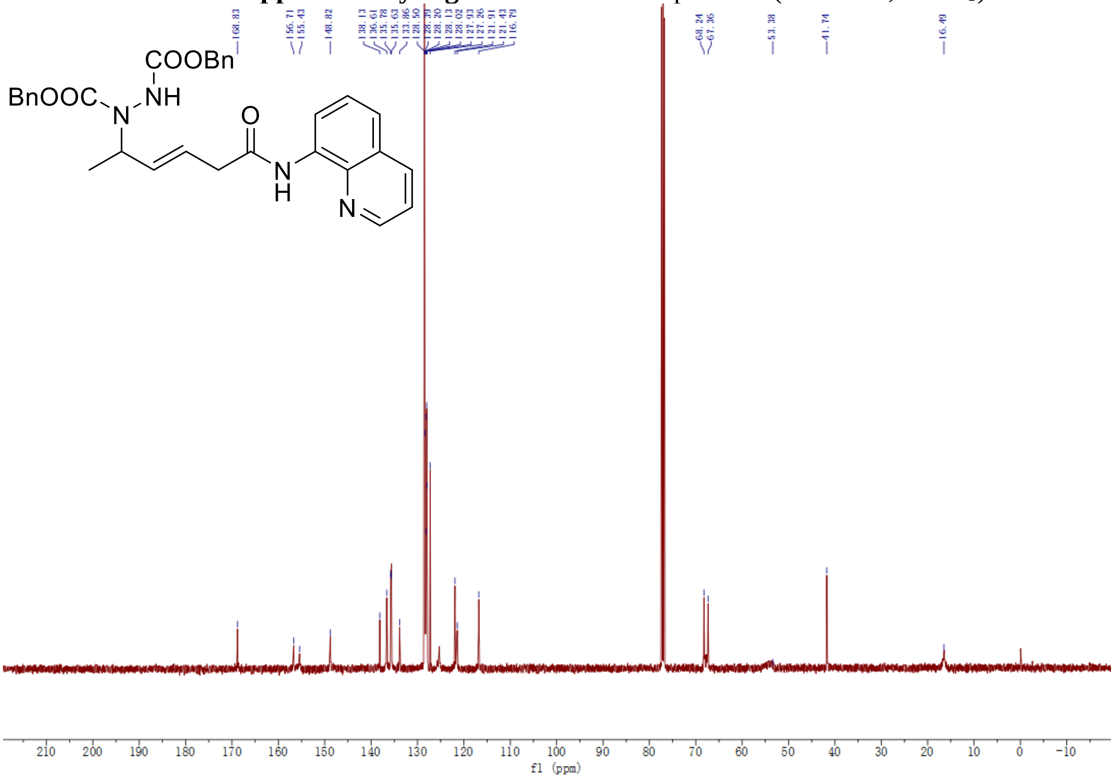

Supplementary Figure 14 ^{1}**H NMR**-spectrum (400 MHz, $CDCl_3$) of **7**

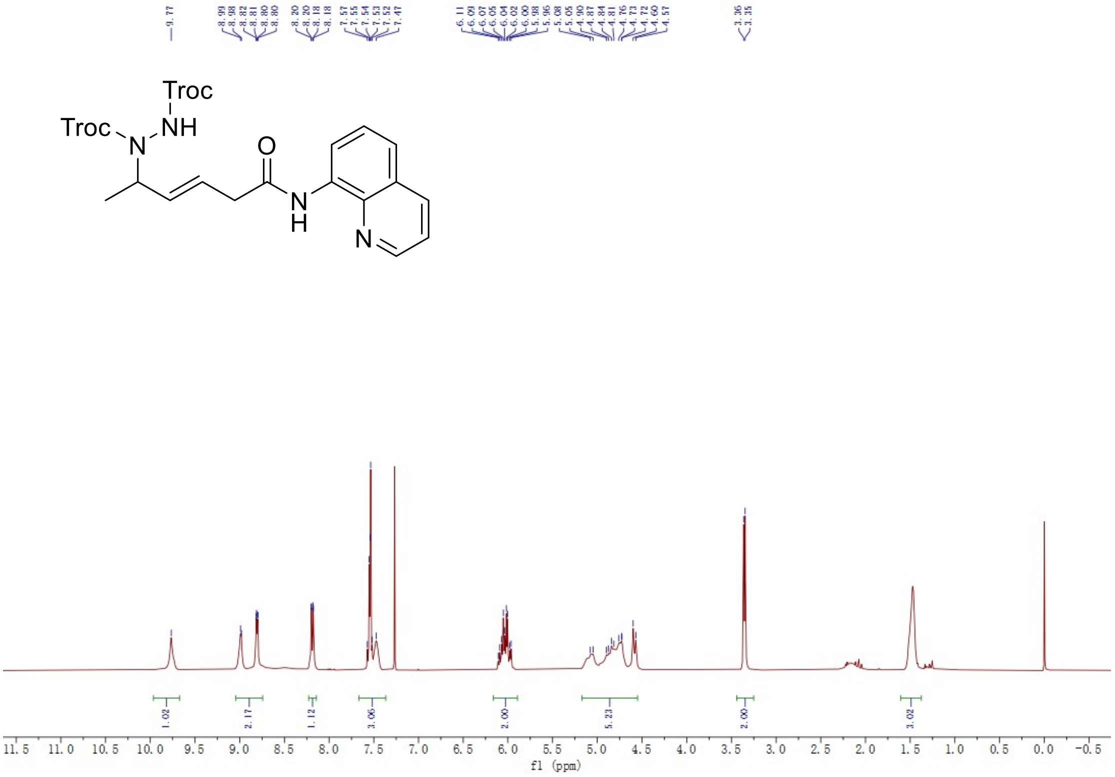

Supplementary Figure 15 ^{13}**C NMR**-spectrum (101 MHz, $CDCl_3$) of **7**

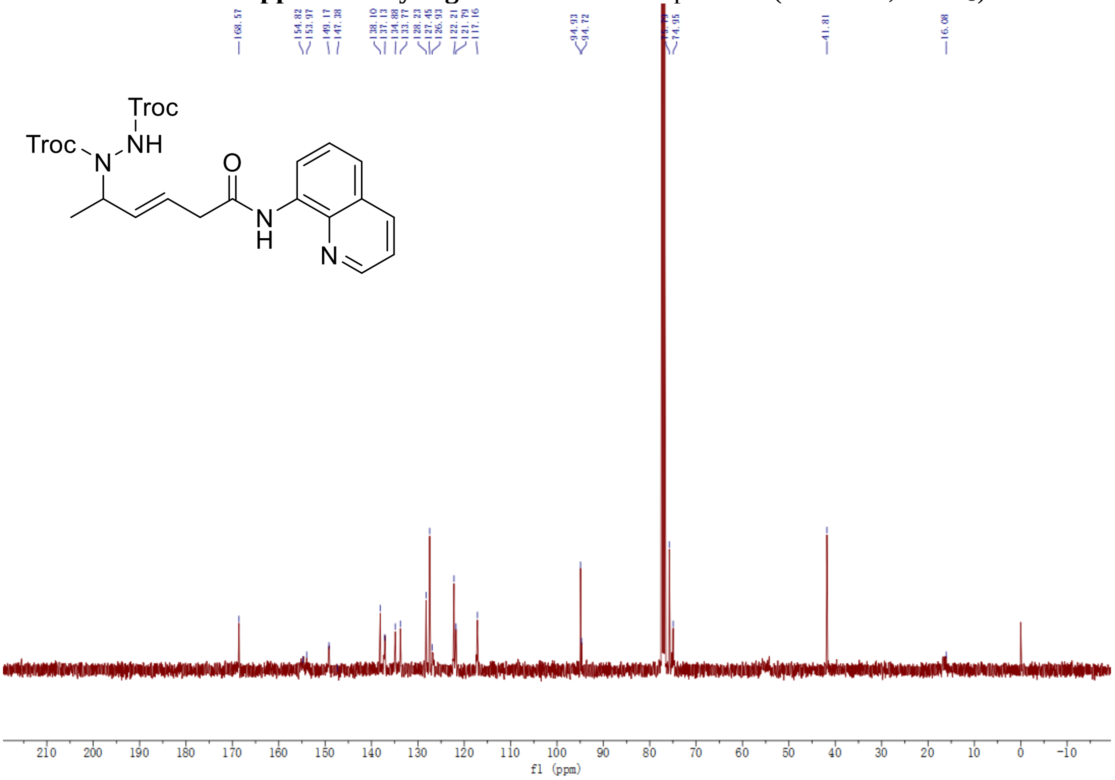

Supplementary Figure 16 ^{1}**H NMR**-spectrum (400 MHz, $CDCl_3$) of **10**

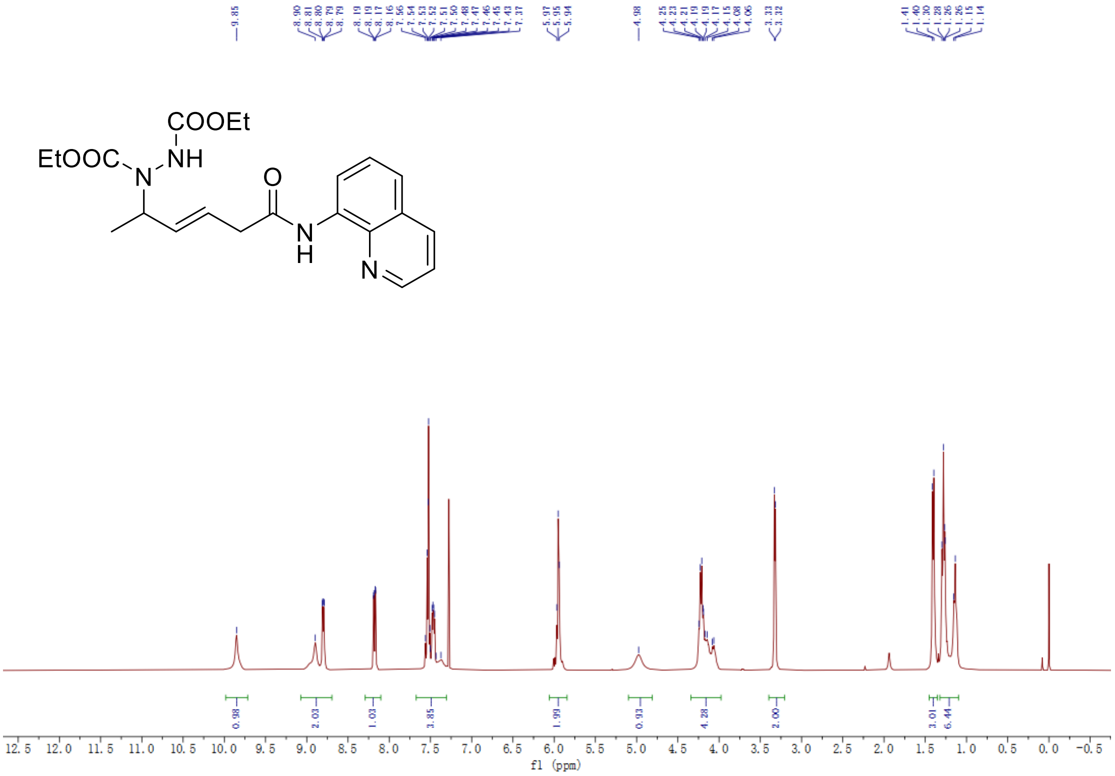

Supplementary Figure 17 ^{13}**C NMR**-spectrum (101 MHz, $CDCl_3$) of **10**

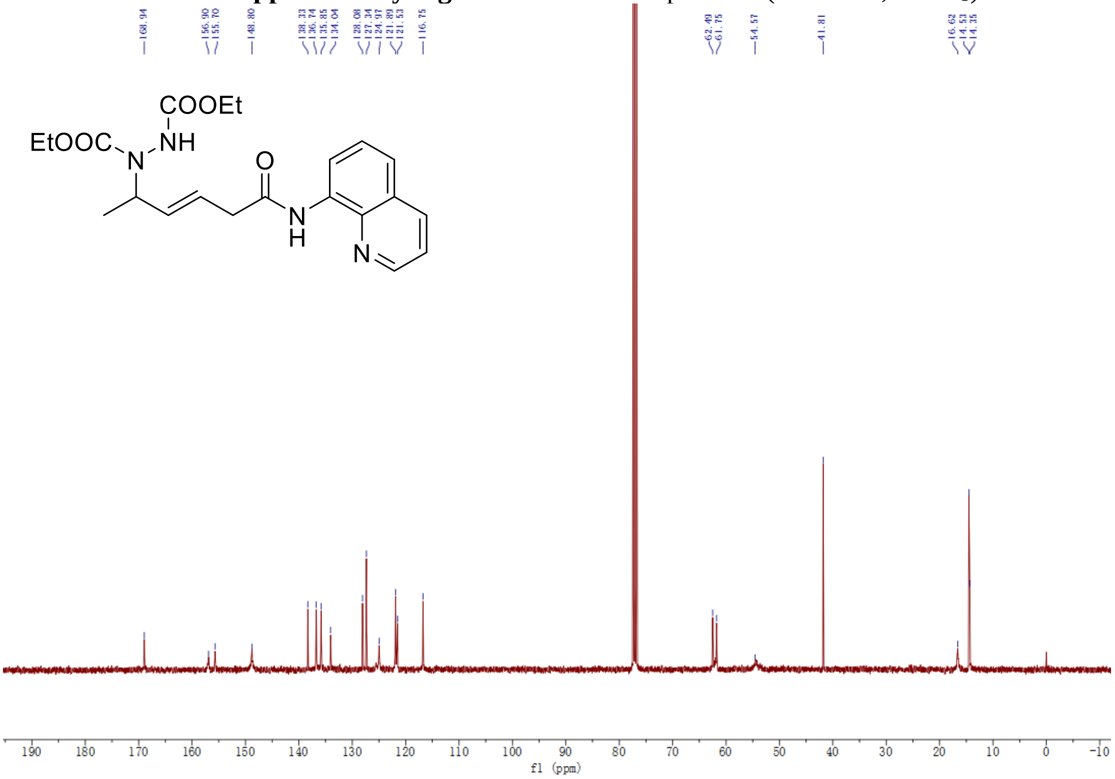

Supplementary Figure 18 ^{1}H NMR-spectrum (400 MHz, $CDCl_3$) of **11**

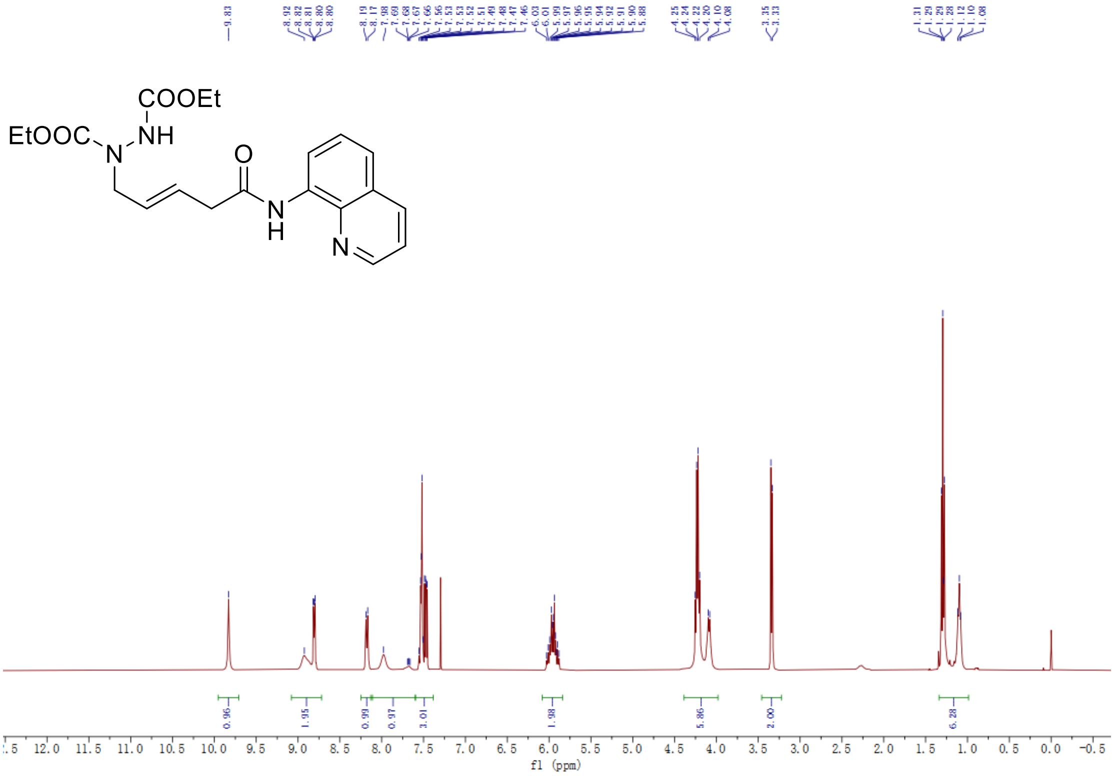

Supplementary Figure 19 ^{13}C NMR-spectrum (101 MHz, $CDCl_3$) of **11**

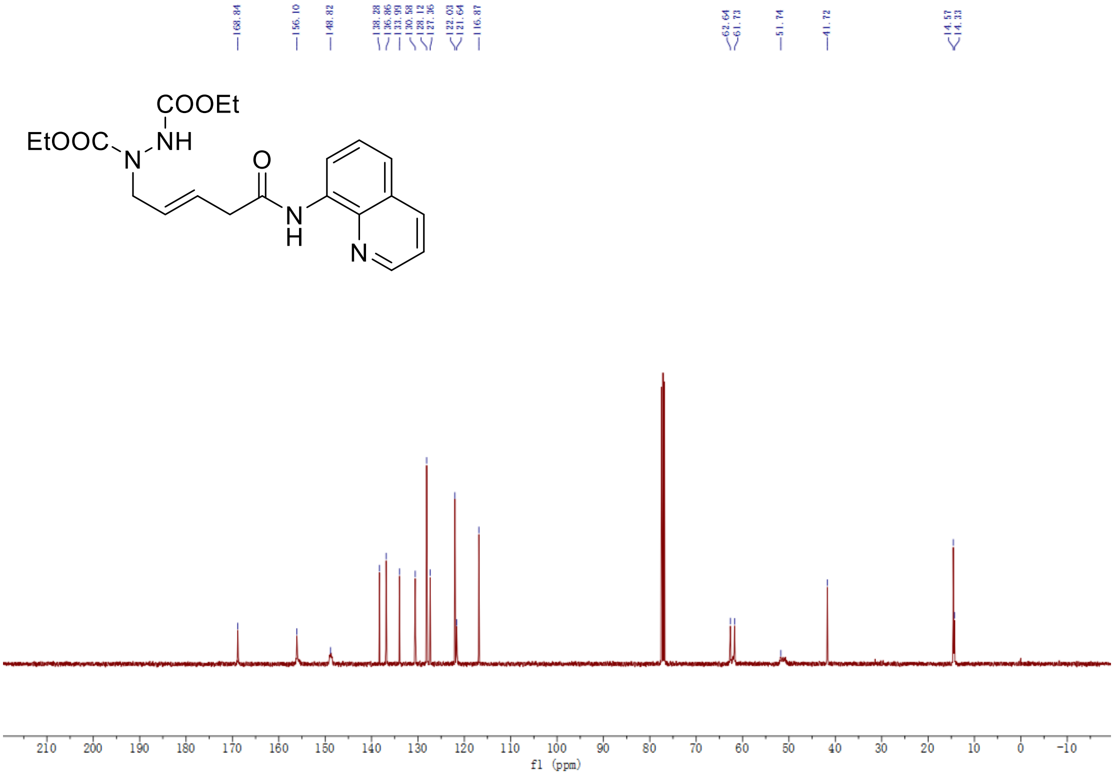

Supplementary Figure 20 ^{1}**H NMR**-spectrum (400 MHz, $CDCl_3$) of **12**

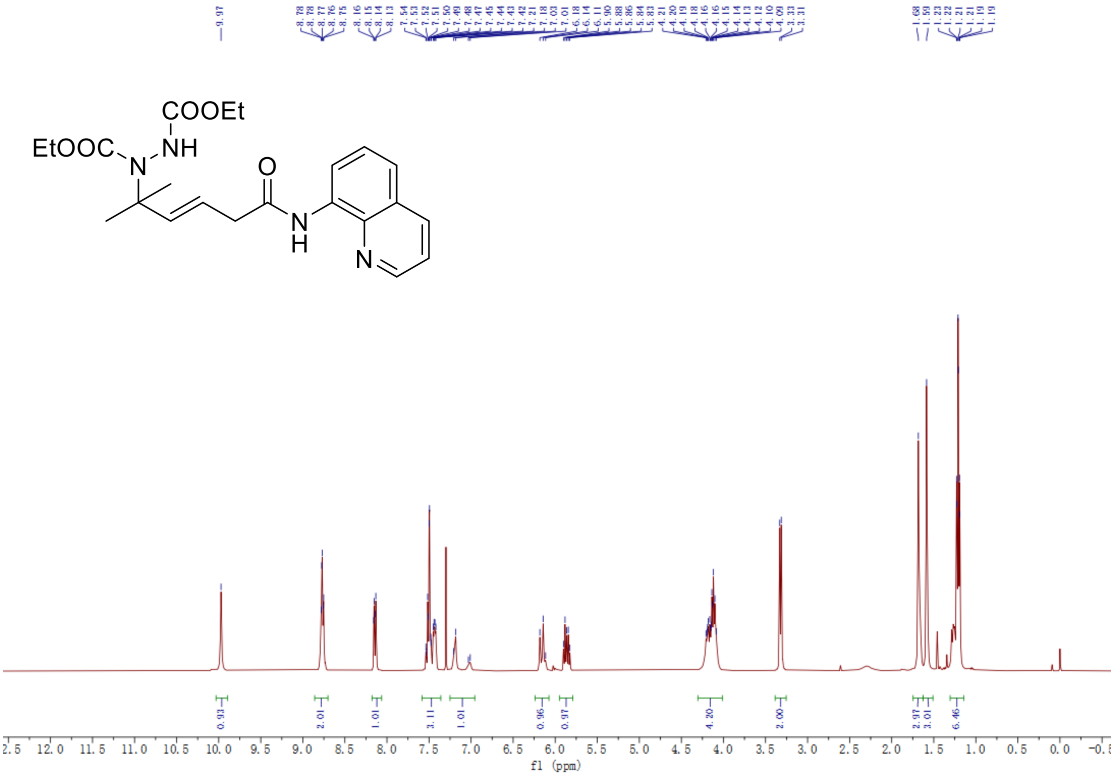

Supplementary Figure 21 ^{13}**C NMR-**spectrum (101 MHz, $CDCl_3$) of **12**

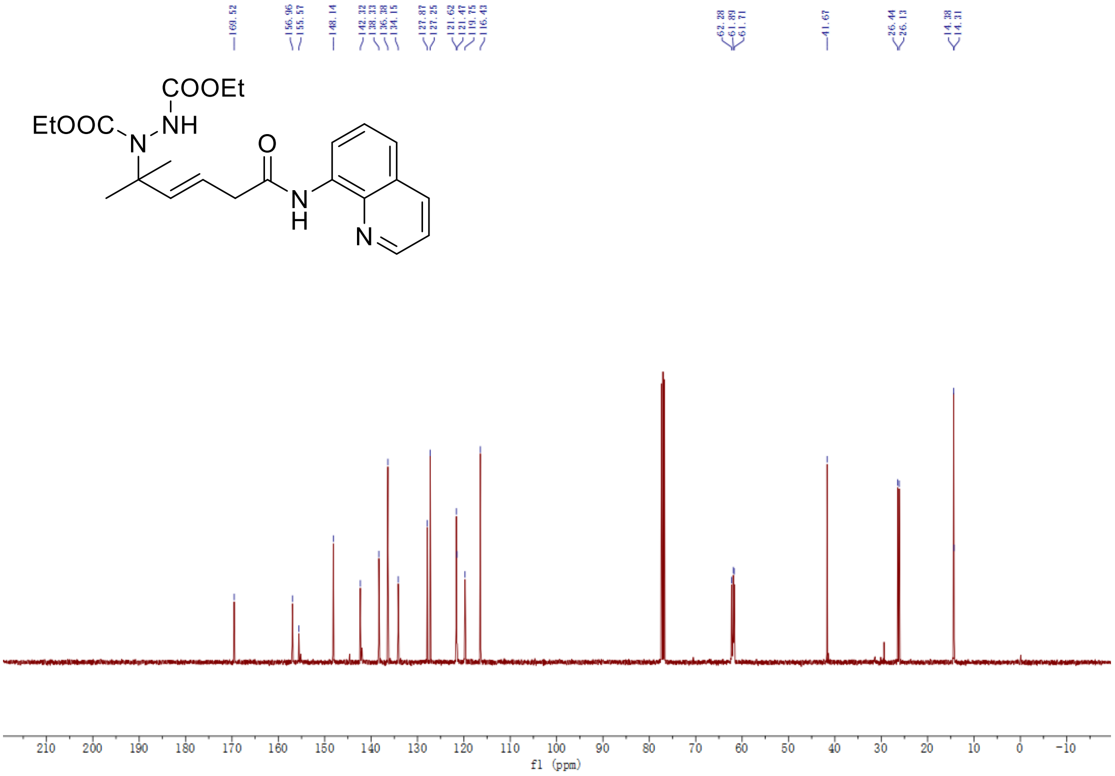

Supplementary Figure 22 ^{1}**H NMR**-spectrum (400 MHz, $CDCl_3$) of **13**

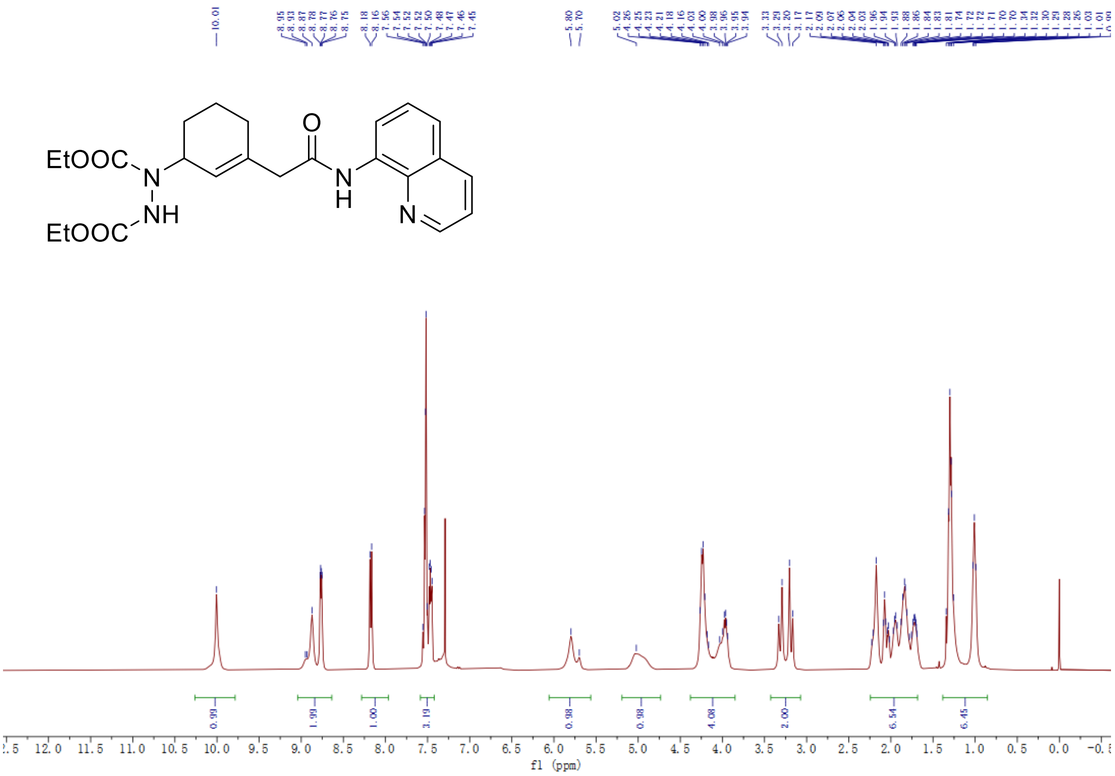

Supplementary Figure 23 ^{13}**C NMR**-spectrum (101 MHz, $CDCl_3$) of **13**

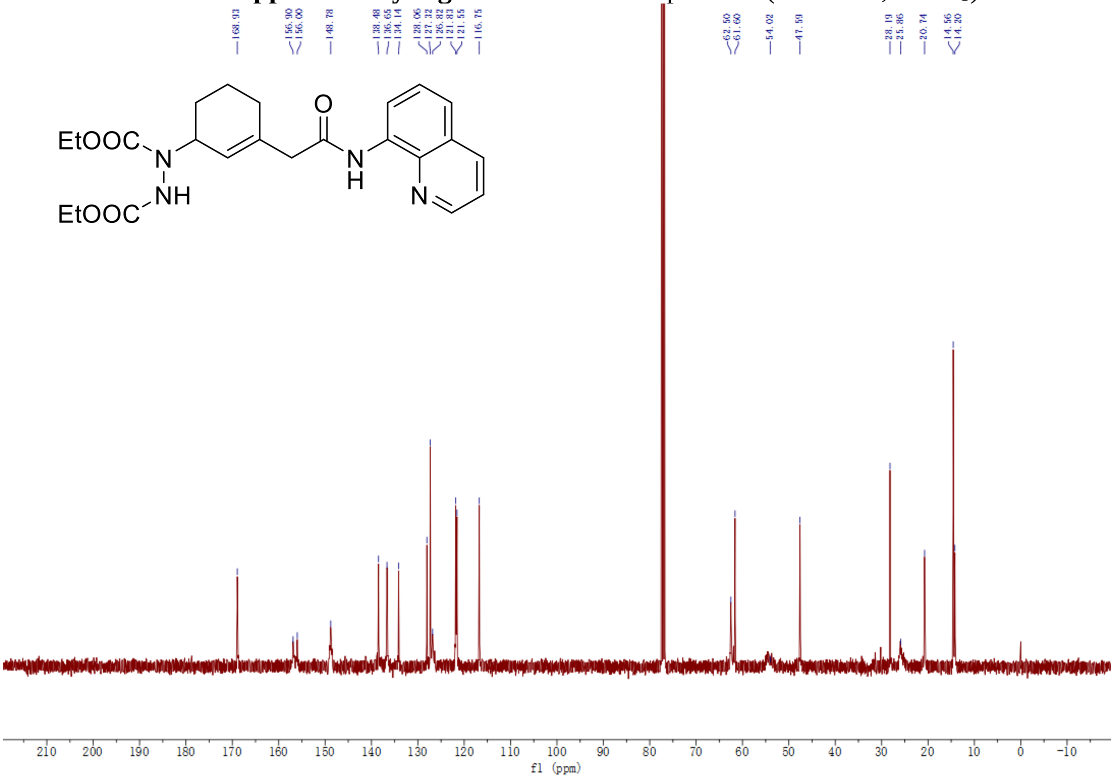

Supplementary Figure 24 ^{1}**H NMR**-spectrum (400 MHz, $CDCl_3$) of **14**

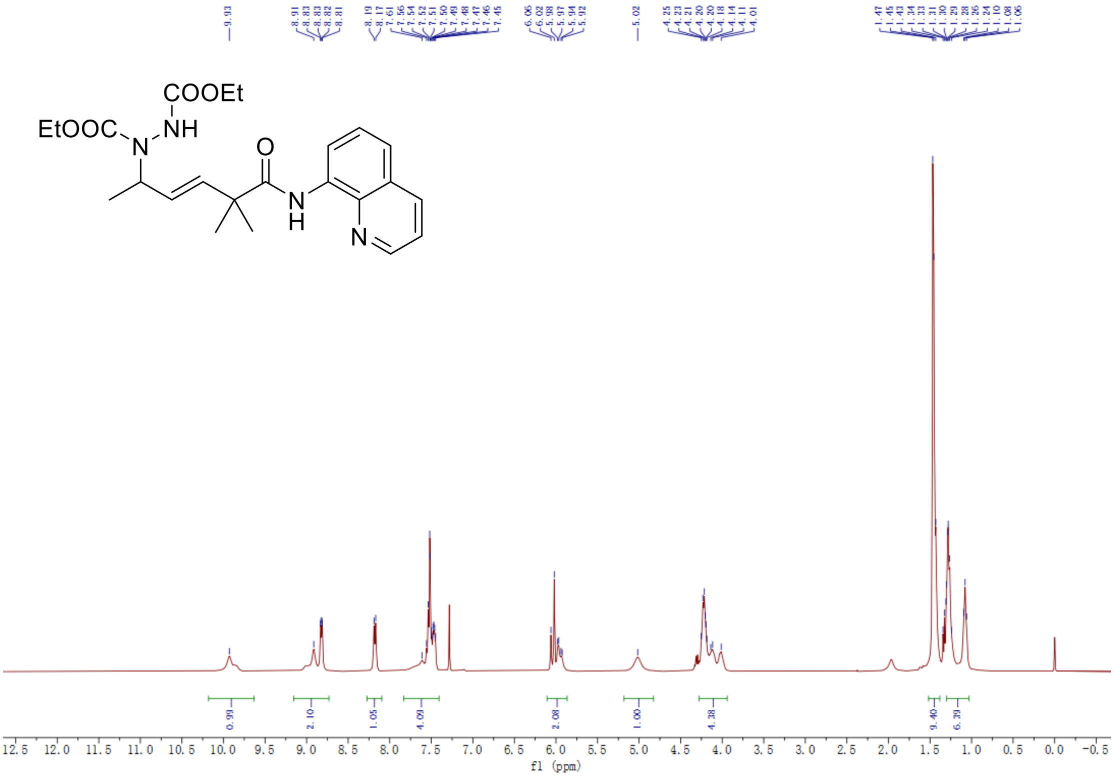

Supplementary Figure 25 ^{13}**C NMR**-spectrum (101 MHz, $CDCl_3$) of **14**

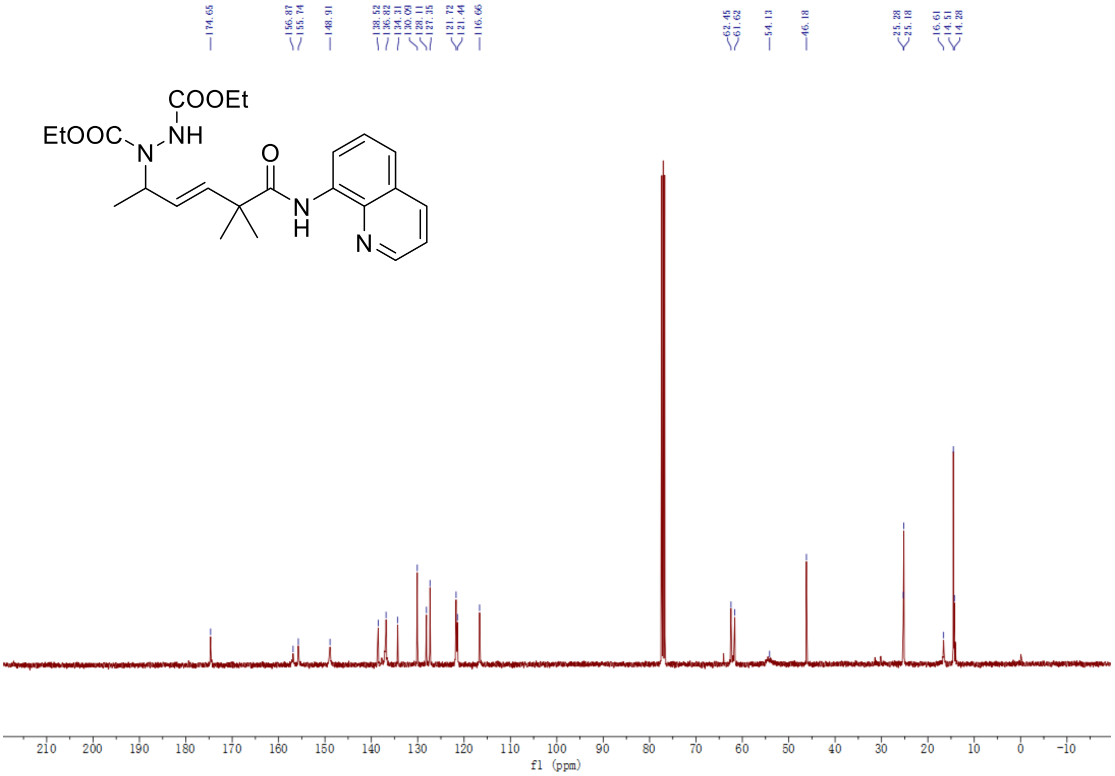

Supplementary Figure 26 ^{1}**H NMR**-spectrum (400 MHz, $CDCl_3$) of **15**

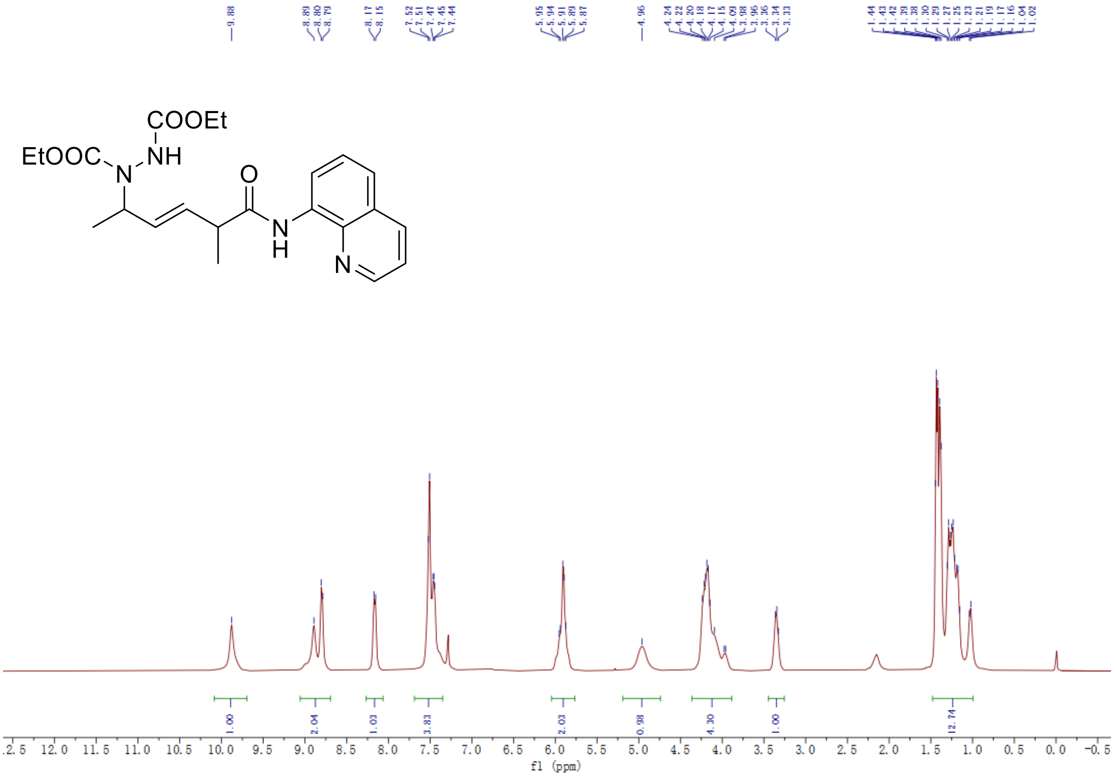

Supplementary Figure 27 ^{13}**C NMR**-spectrum (101 MHz, $CDCl_3$) of **15**

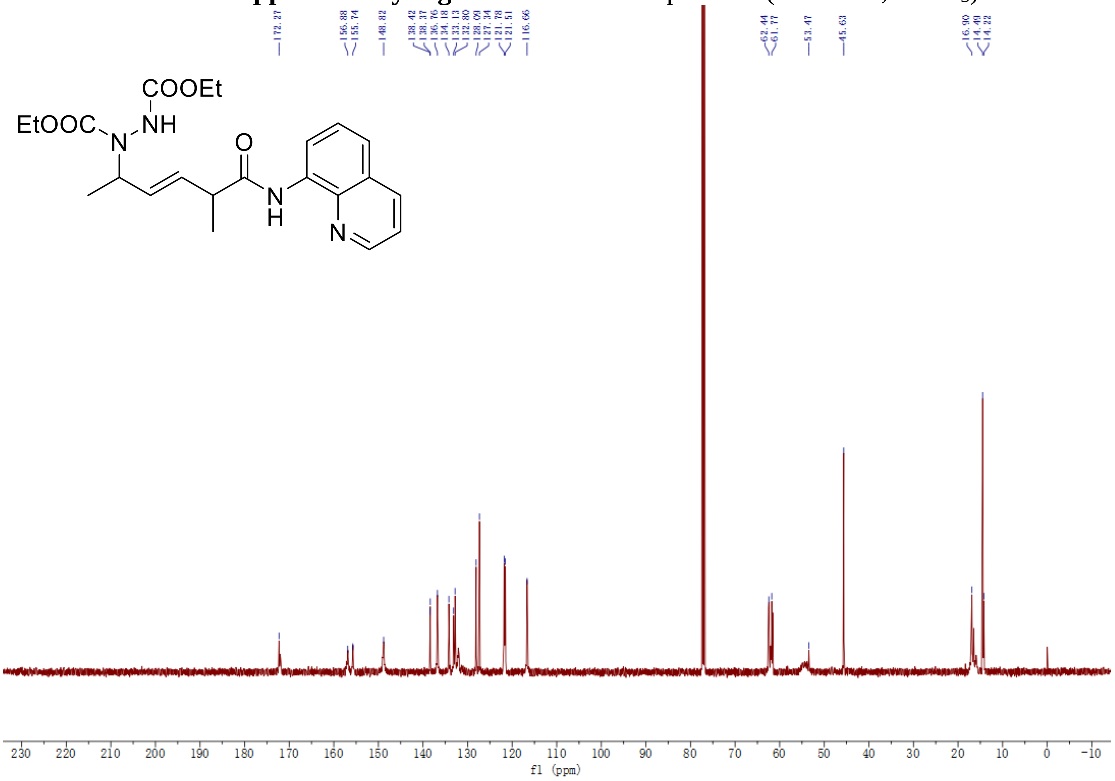

Supplementary Figure 28 ^{1}**H NMR**-spectrum (400 MHz, $CDCl_3$) of **16**

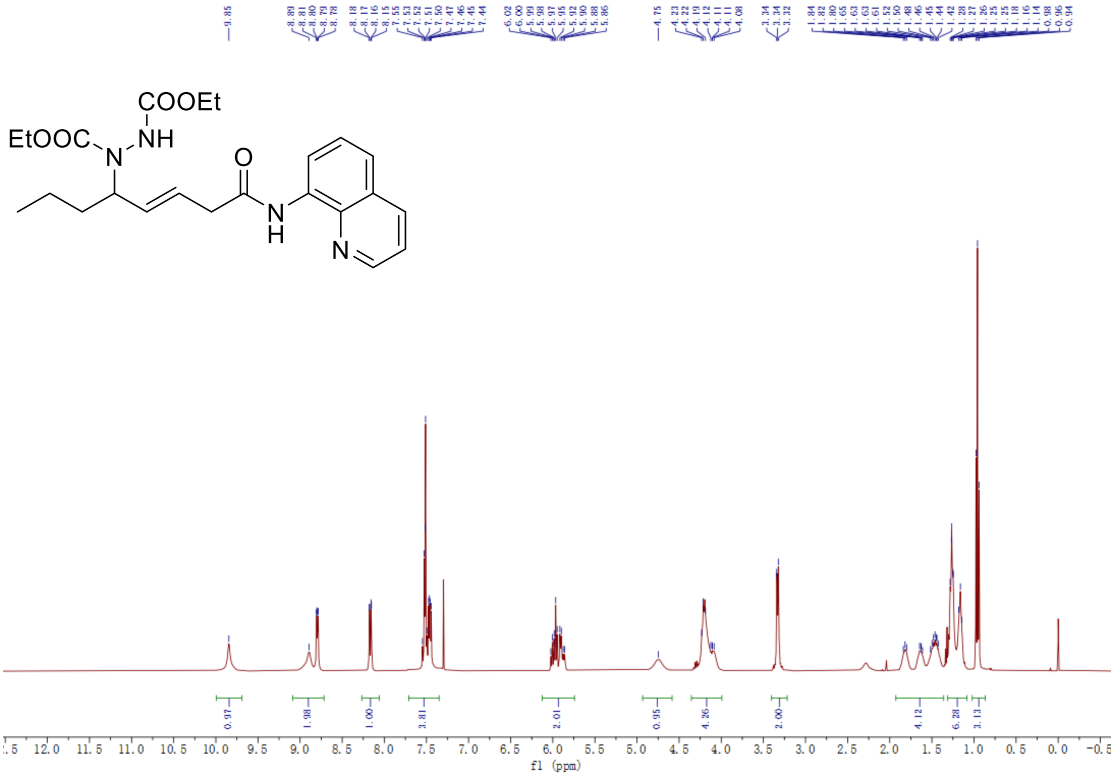

Supplementary Figure 29 ^{13}**C NMR**-spectrum (101 MHz, $CDCl_3$) of **16**

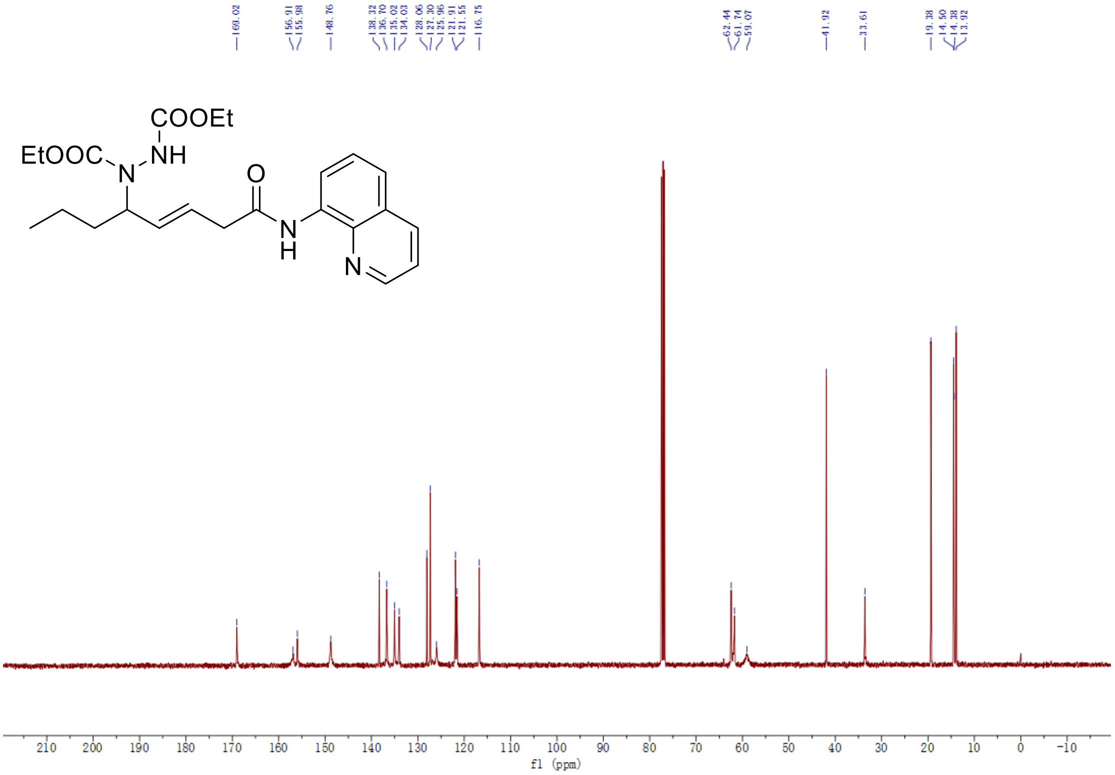

Supplementary Figure 30 ^{1}**H NMR**-spectrum (400 MHz, $CDCl_3$) of **17**

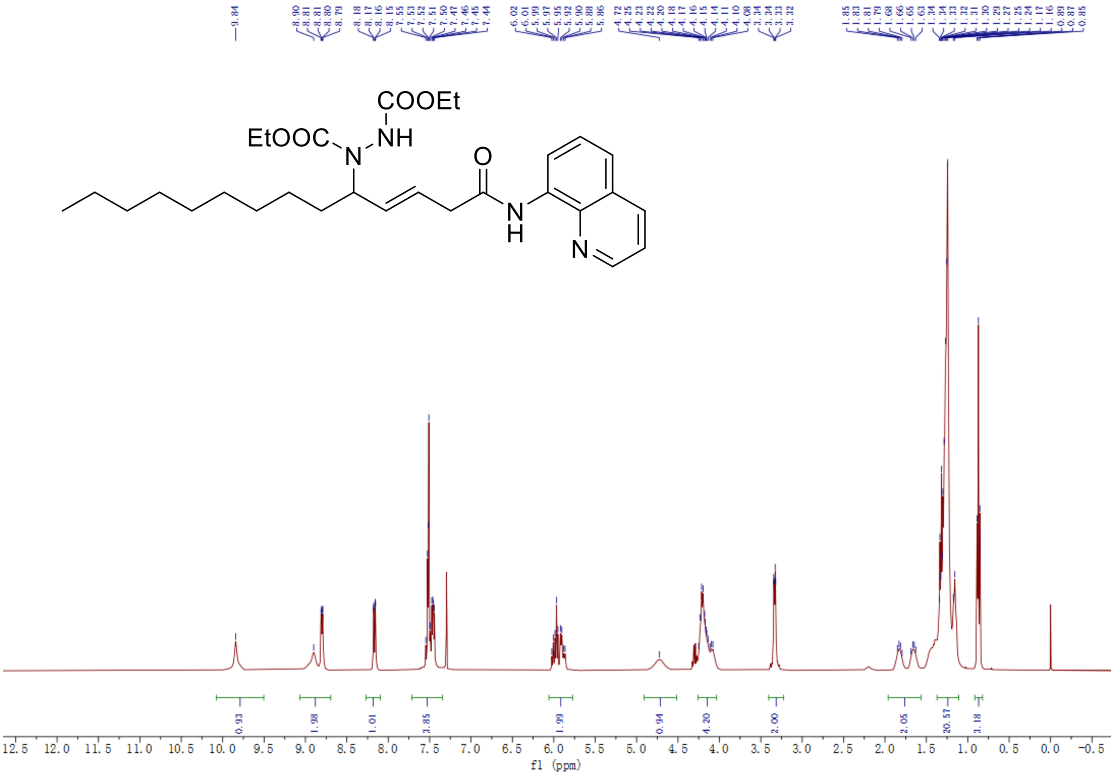

Supplementary Figure 31 ^{13}**C NMR**-spectrum (101 MHz, $CDCl_3$) of **17**

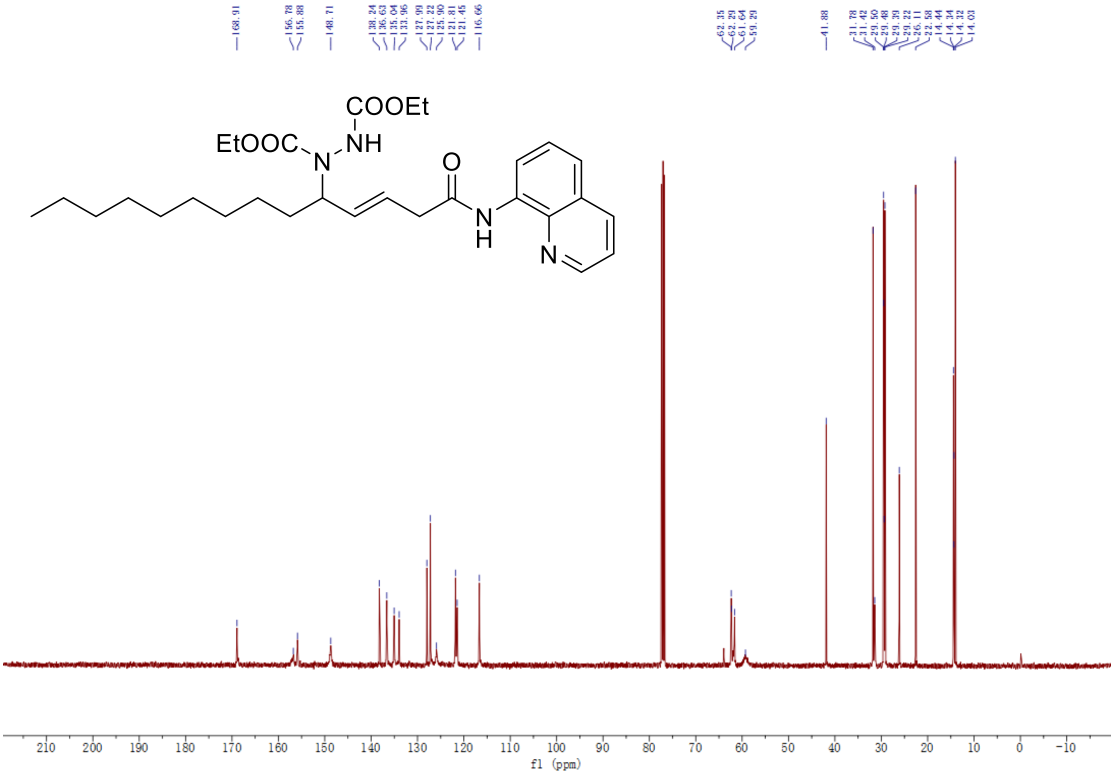

Supplementary Figure 32 ^{1}**H NMR**-spectrum (400 MHz, $CDCl_3$) of **18**

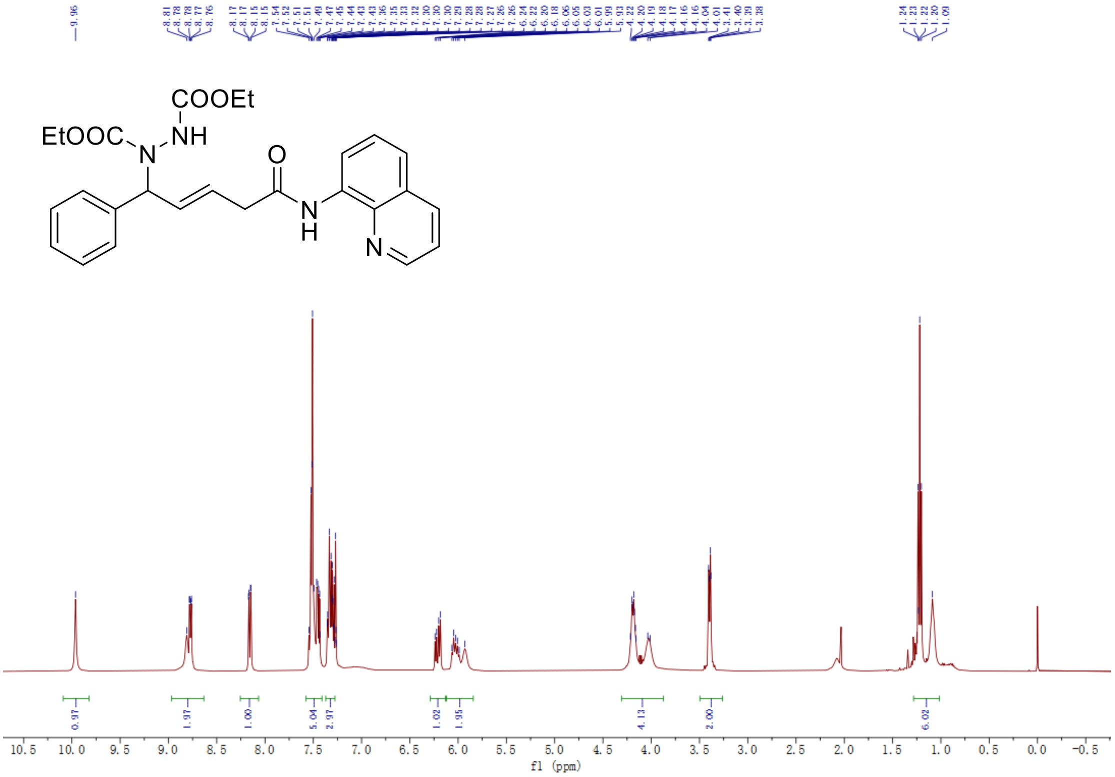

Supplementary Figure 33 ^{13}**C NMR**-spectrum (101 MHz, $CDCl_3$) of **18**

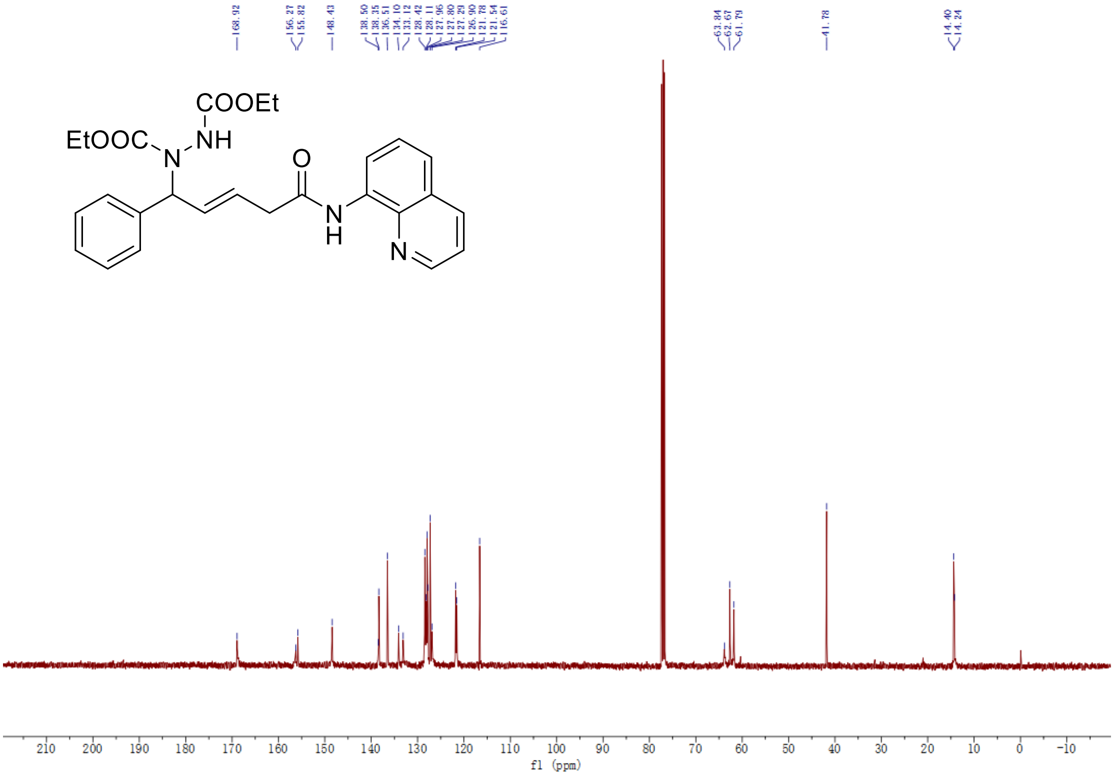

Supplementary Figure 34 ^{1}**H NMR**-spectrum (400 MHz, $CDCl_3$) of **19**

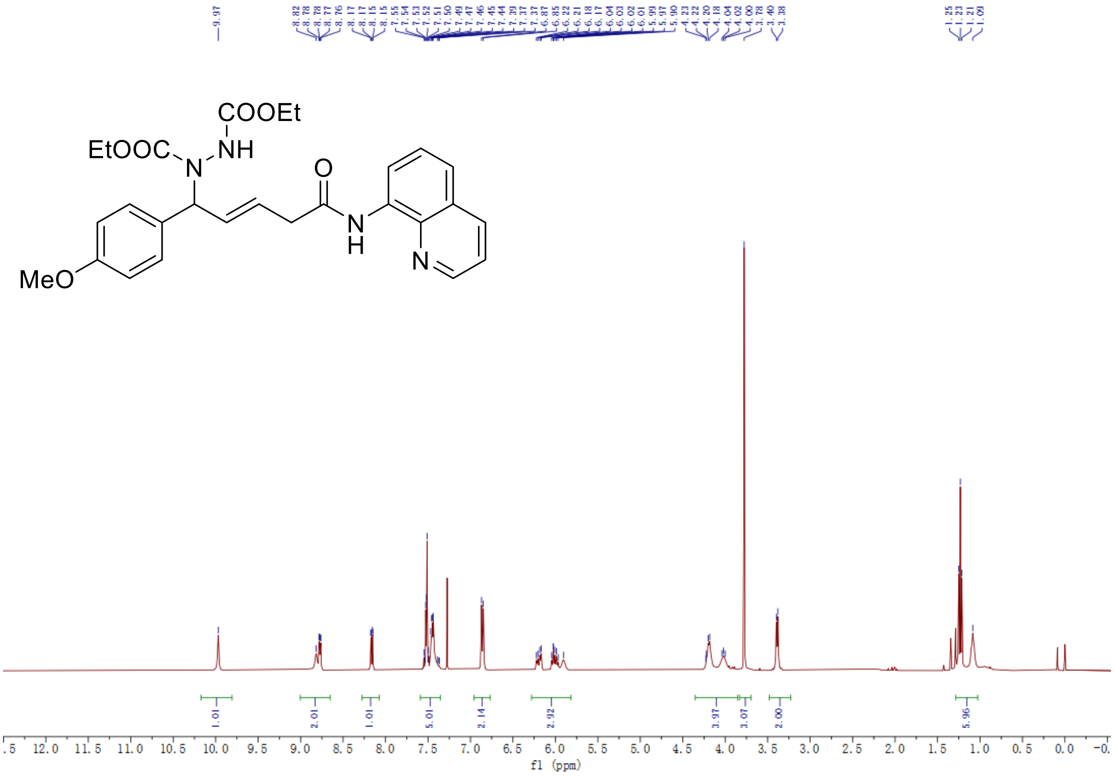

Supplementary Figure 35 ^{13}**C NMR**-spectrum (101 MHz, $CDCl_3$) of **19**

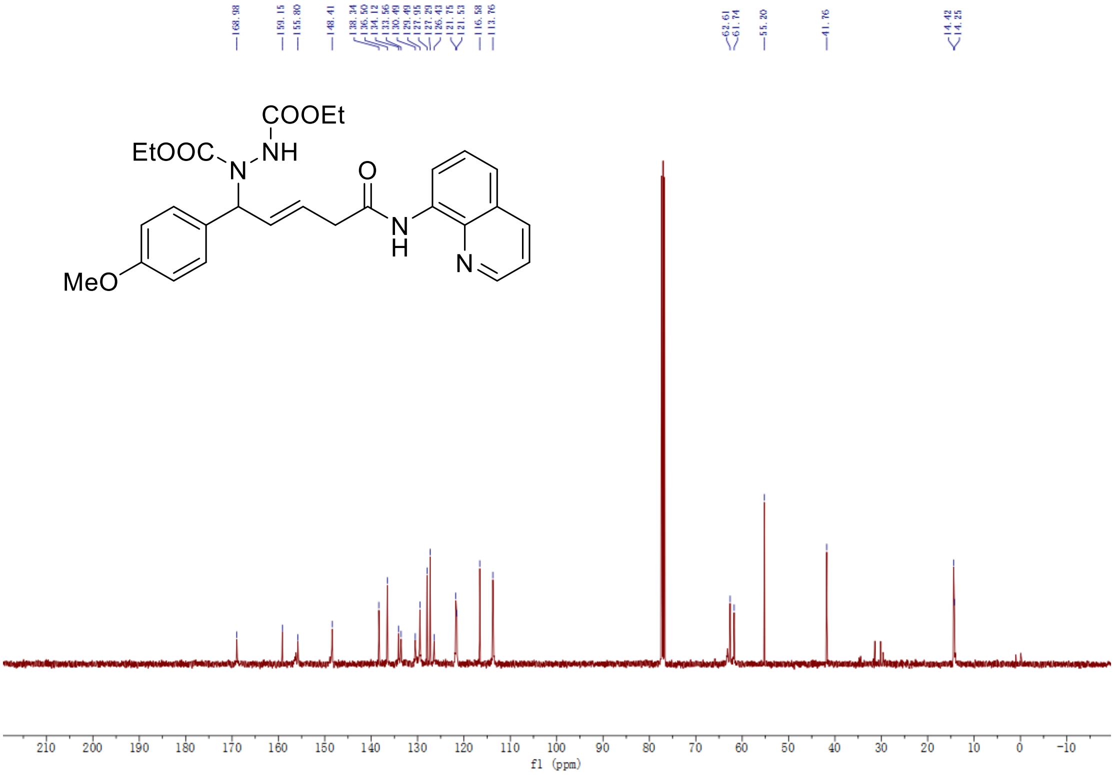

Supplementary Figure 36 ^{1}**H NMR**-spectrum (400 MHz, $CDCl_3$) of **20**

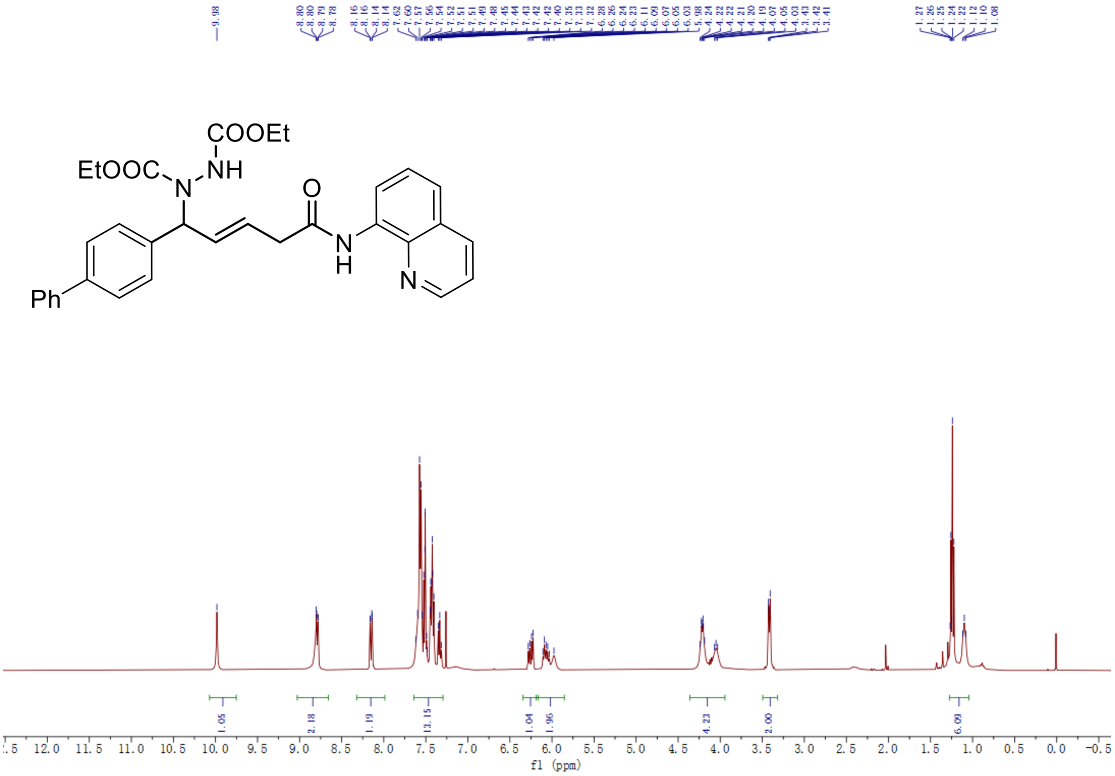

Supplementary Figure 37 ^{13}**C NMR**-spectrum (101 MHz, $CDCl_3$) of **20**

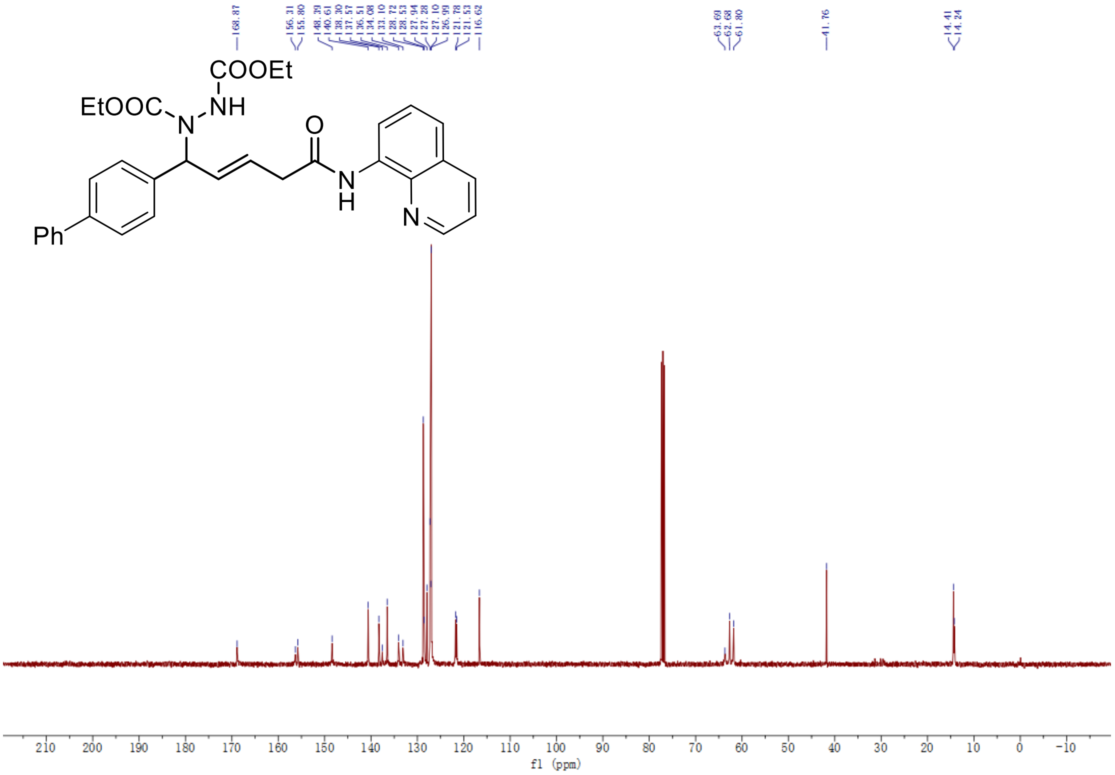

Supplementary Figure 38 ^{1}**H NMR**-spectrum (400 MHz, $CDCl_3$) of **21**

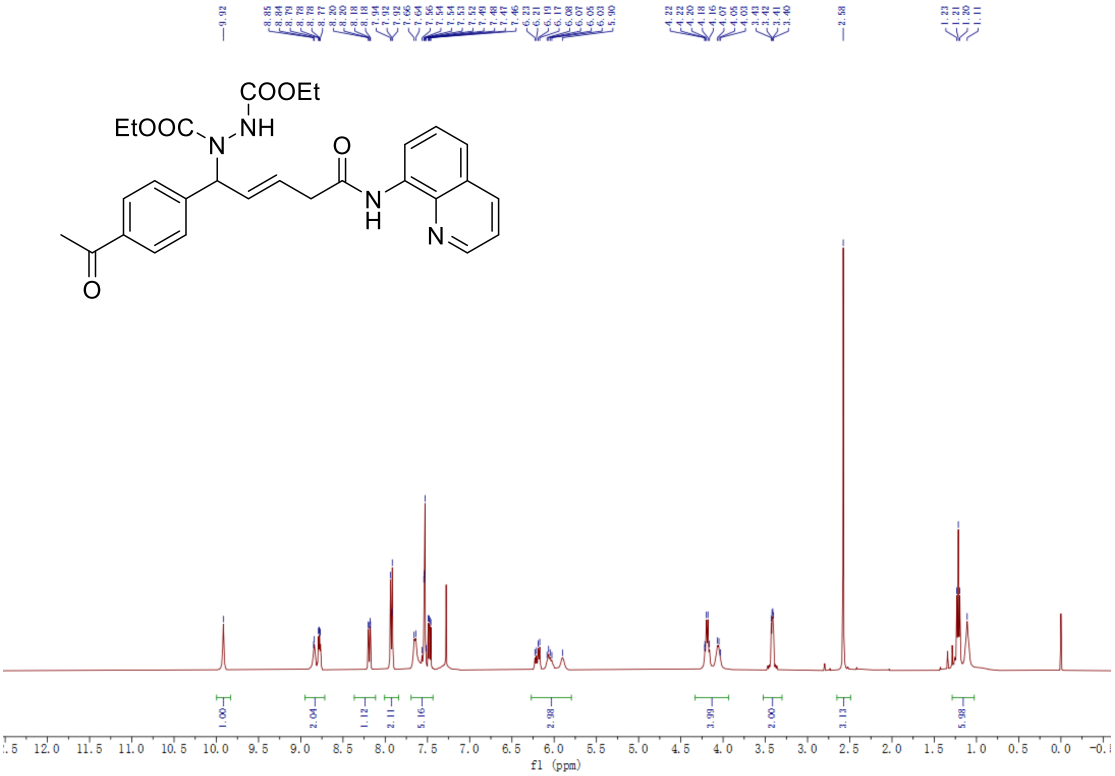

Supplementary Figure 39 ^{13}**C NMR**-spectrum (101 MHz, $CDCl_3$) of **21**

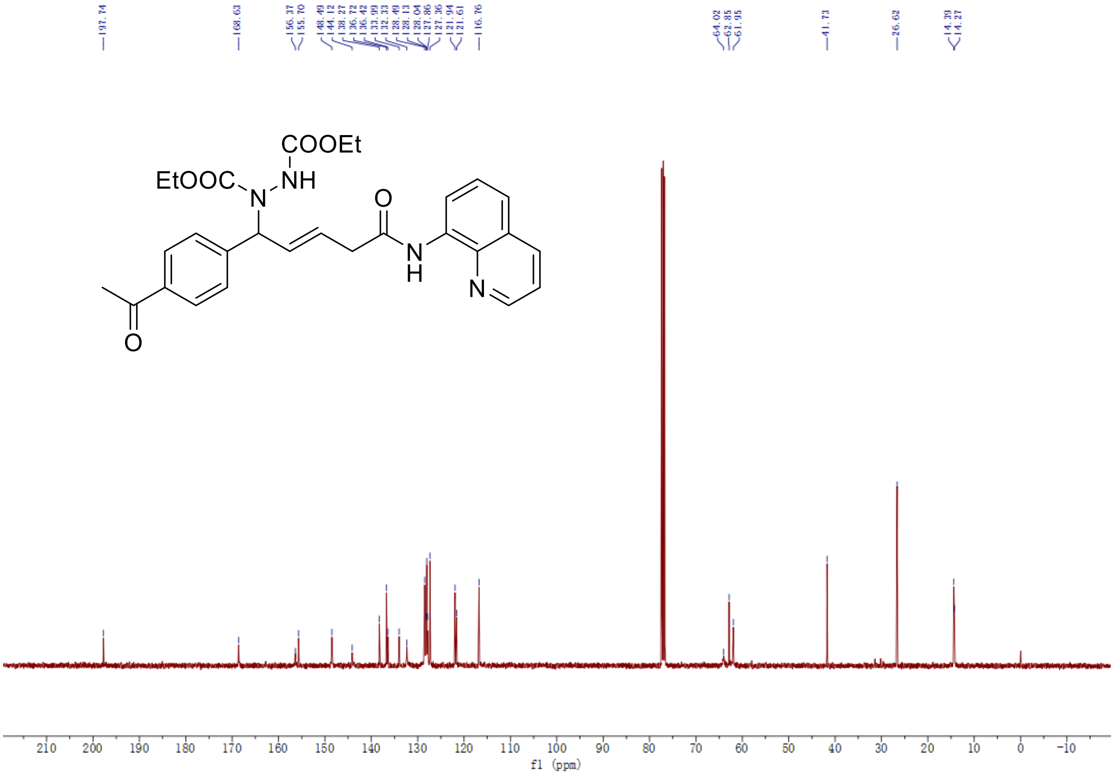

Supplementary Figure 40 ^{1}**H NMR**-spectrum (400 MHz, $CDCl_3$) of **22**

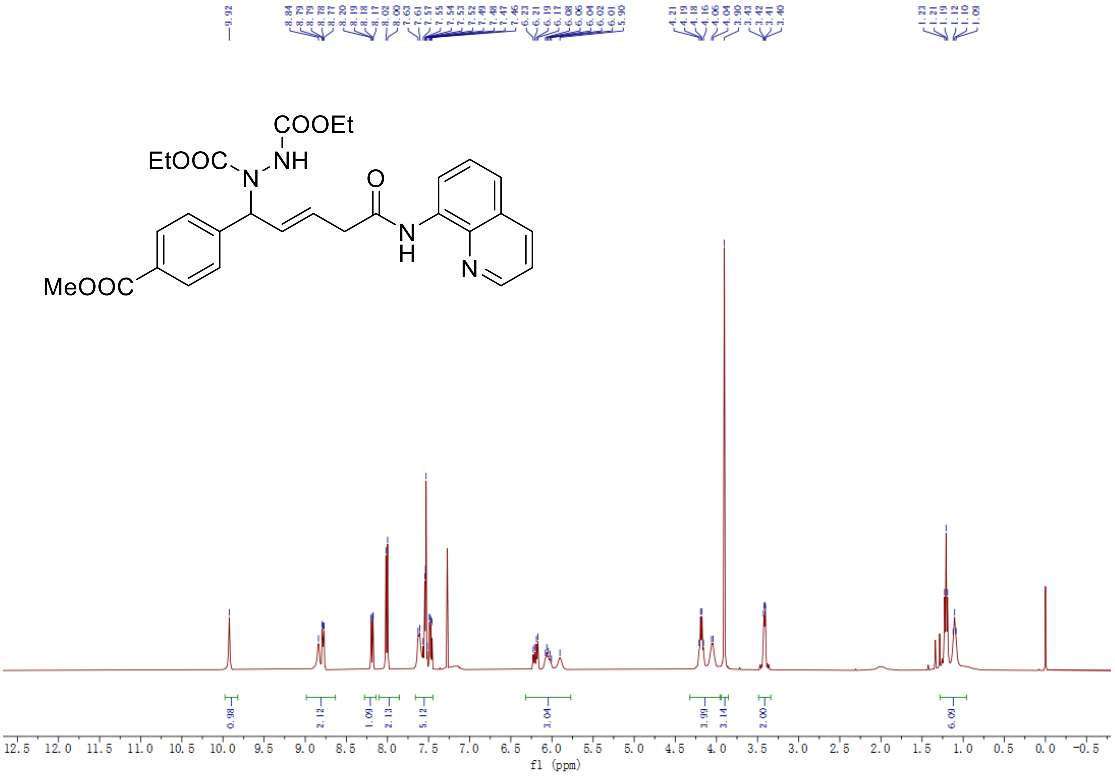

Supplementary Figure 41 ^{13}**C NMR**-spectrum (101 MHz, $CDCl_3$) of **22**

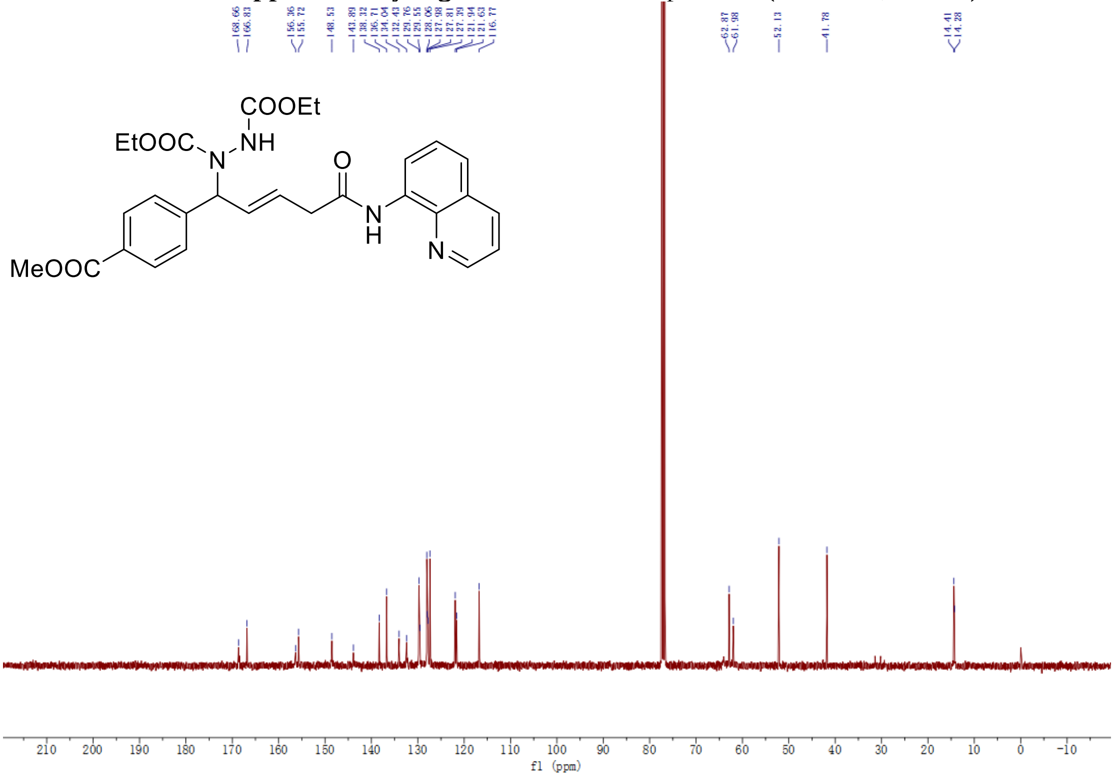

Supplementary Figure 42 ^{1}**H NMR**-spectrum (400 MHz, $CDCl_3$) of **23**

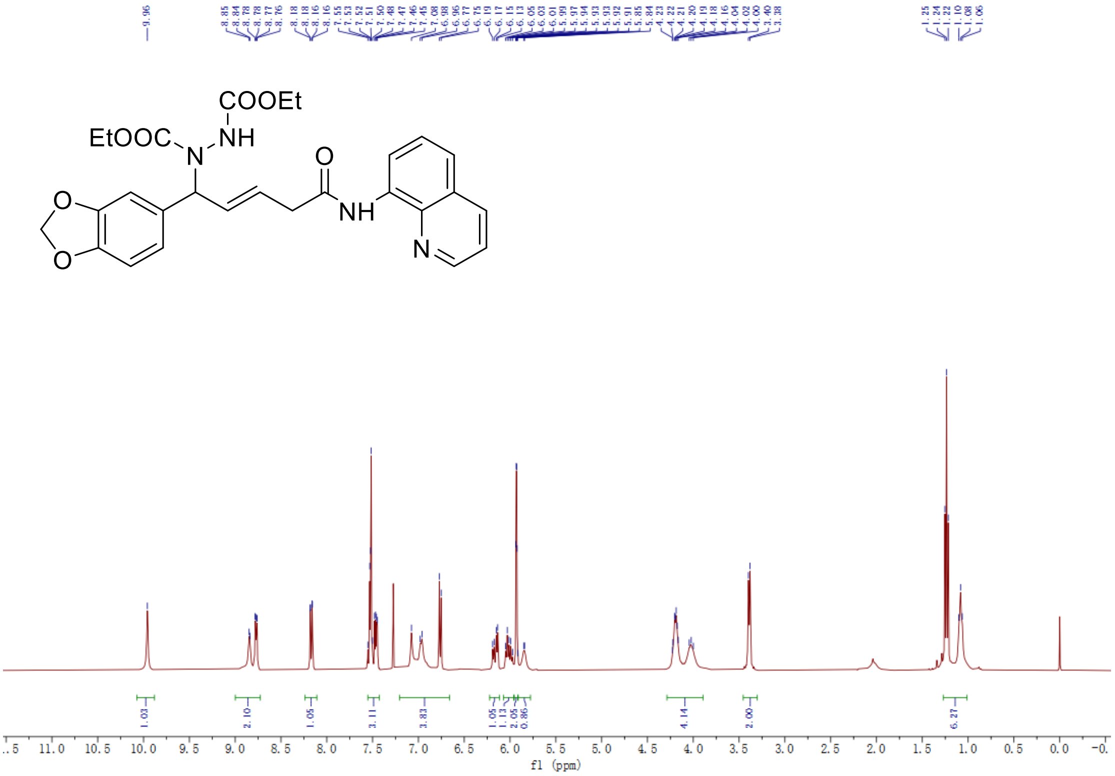

Supplementary Figure 43 ^{13}**C NMR**-spectrum (101 MHz, $CDCl_3$) of **23**

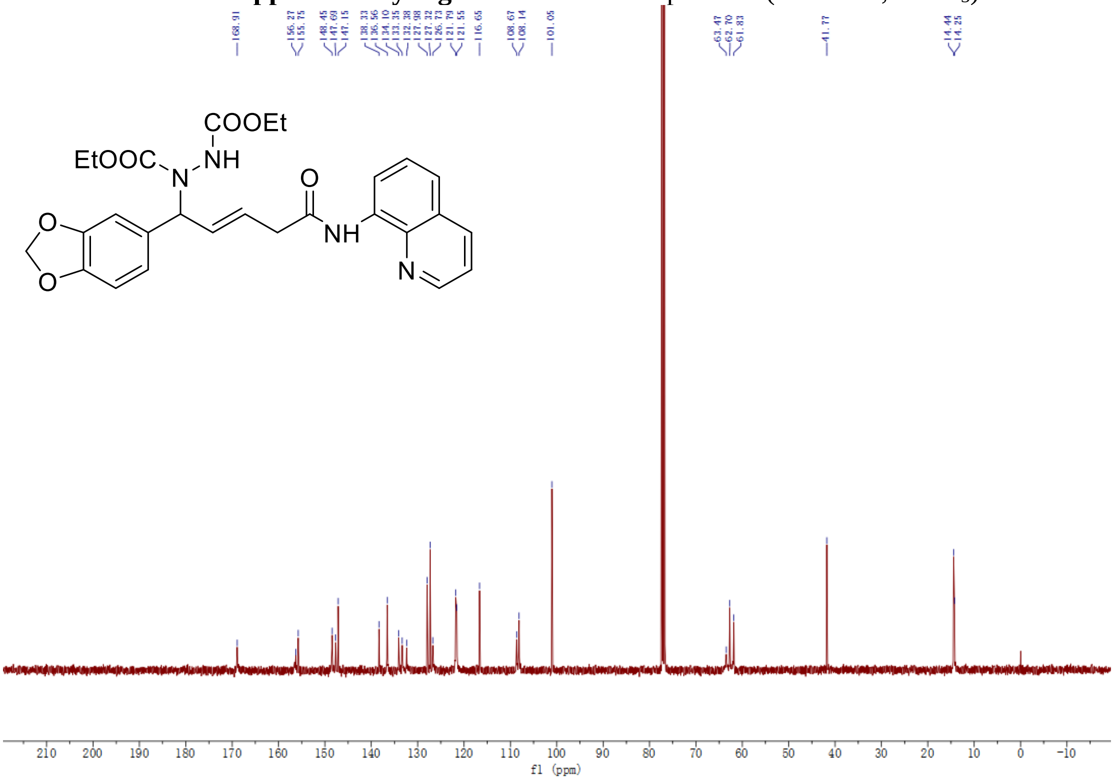

Supplementary Figure 44 ^{1}H NMR-spectrum (400 MHz, $CDCl_3$) of **24**

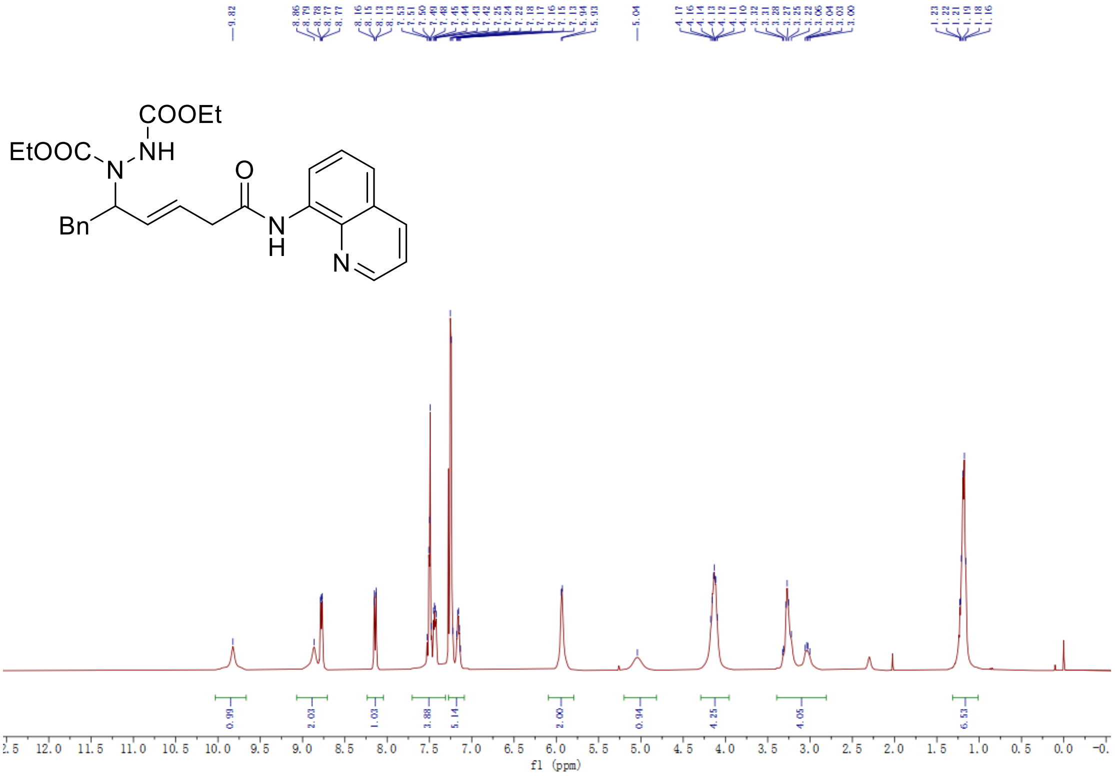

Supplementary Figure 45 ^{13}C NMR-spectrum (101 MHz, $CDCl_3$) of **24**

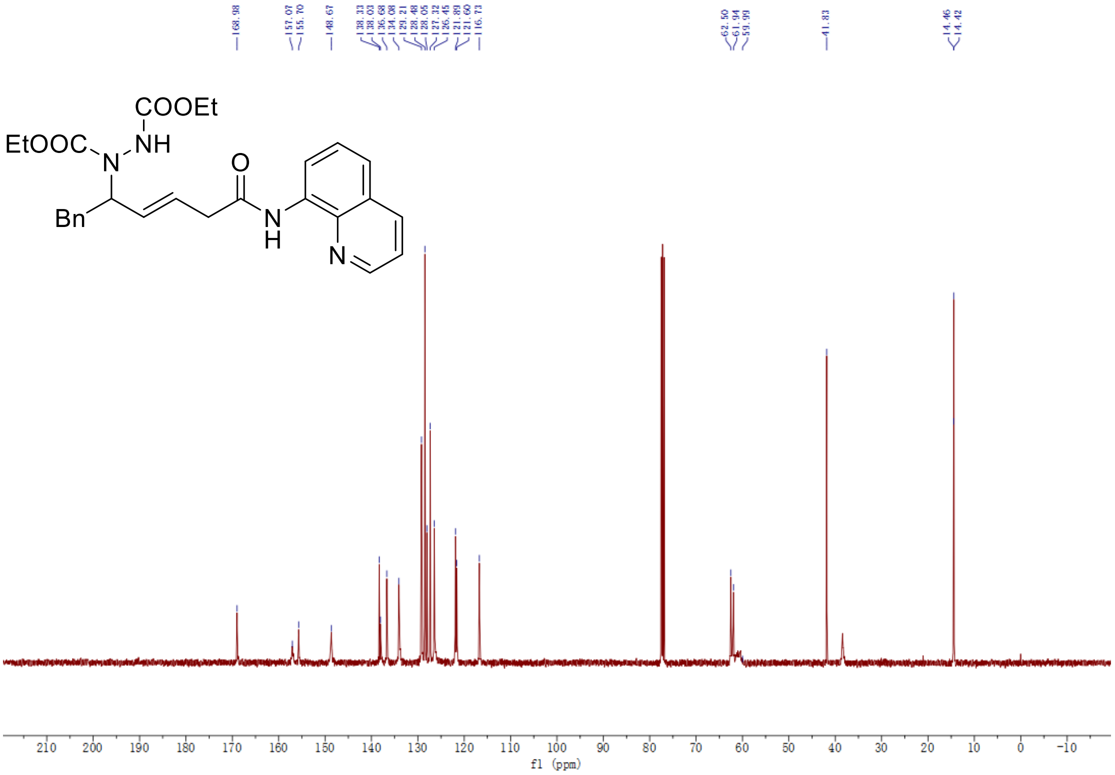

Supplementary Figure 46 ^{1}**H NMR**-spectrum (400 MHz, $CDCl_3$) of **25**

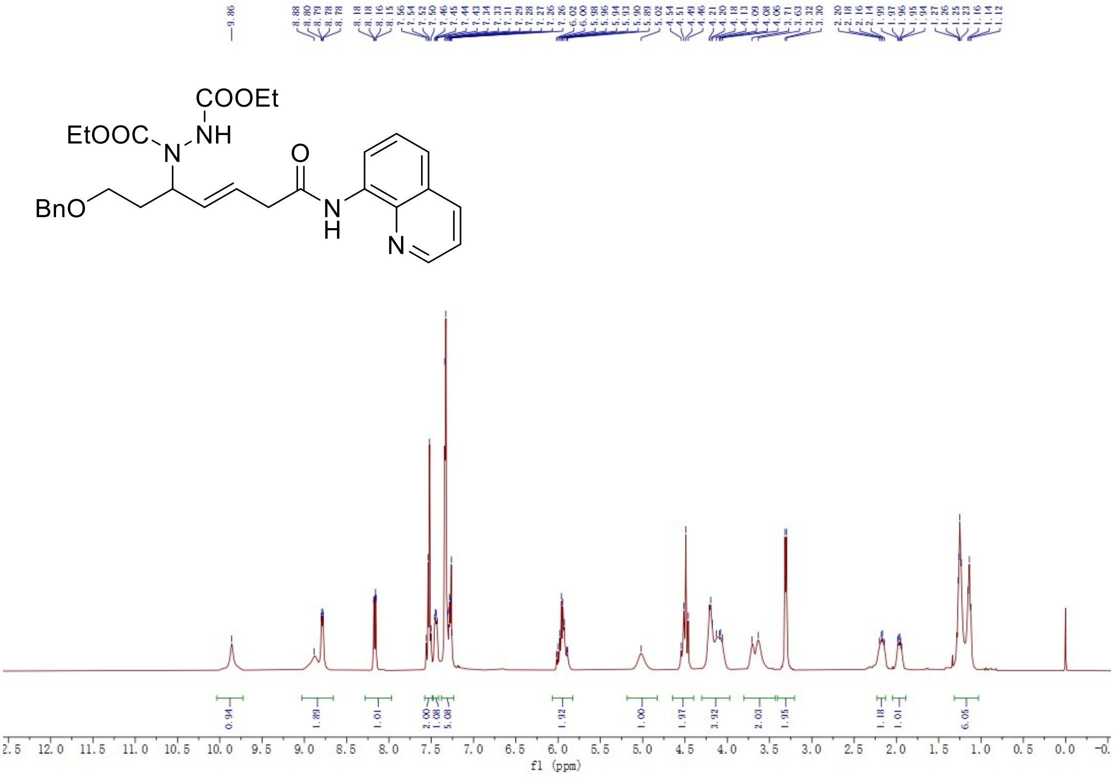

Supplementary Figure 47 ^{13}**C NMR-**spectrum (101 MHz, $CDCl_3$) of **25**

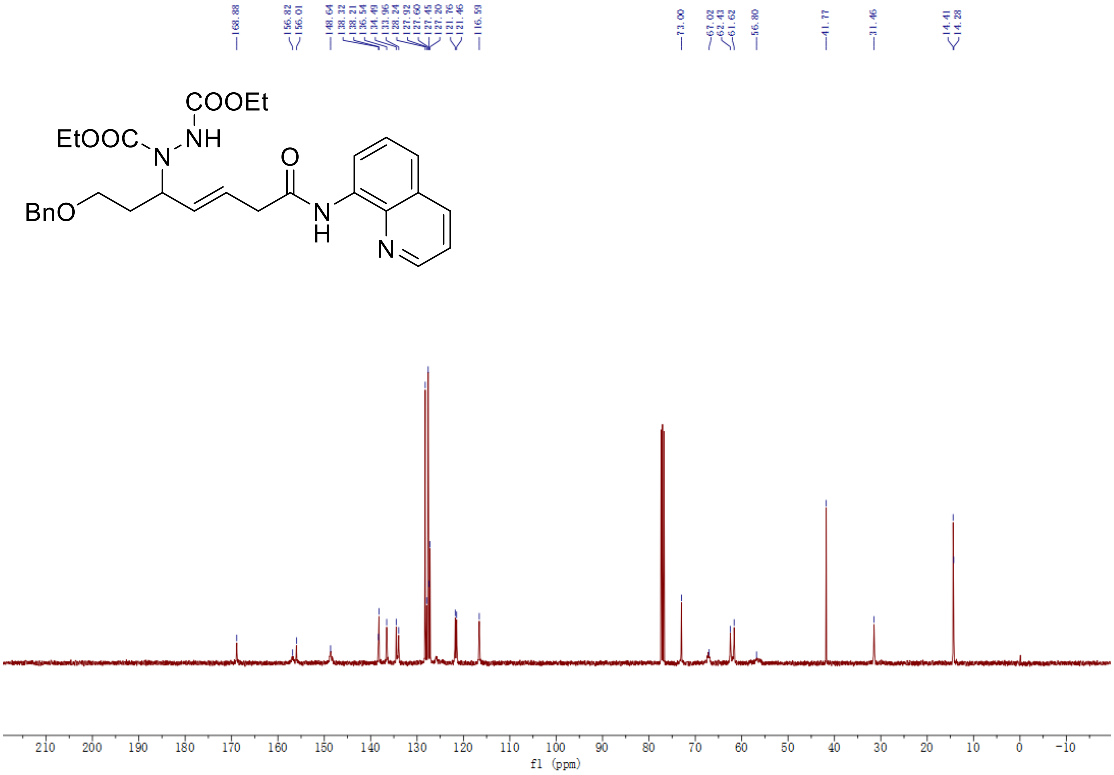

Supplementary Figure 48 ^{1}**H NMR**-spectrum (400 MHz, $CDCl_3$) of **26**

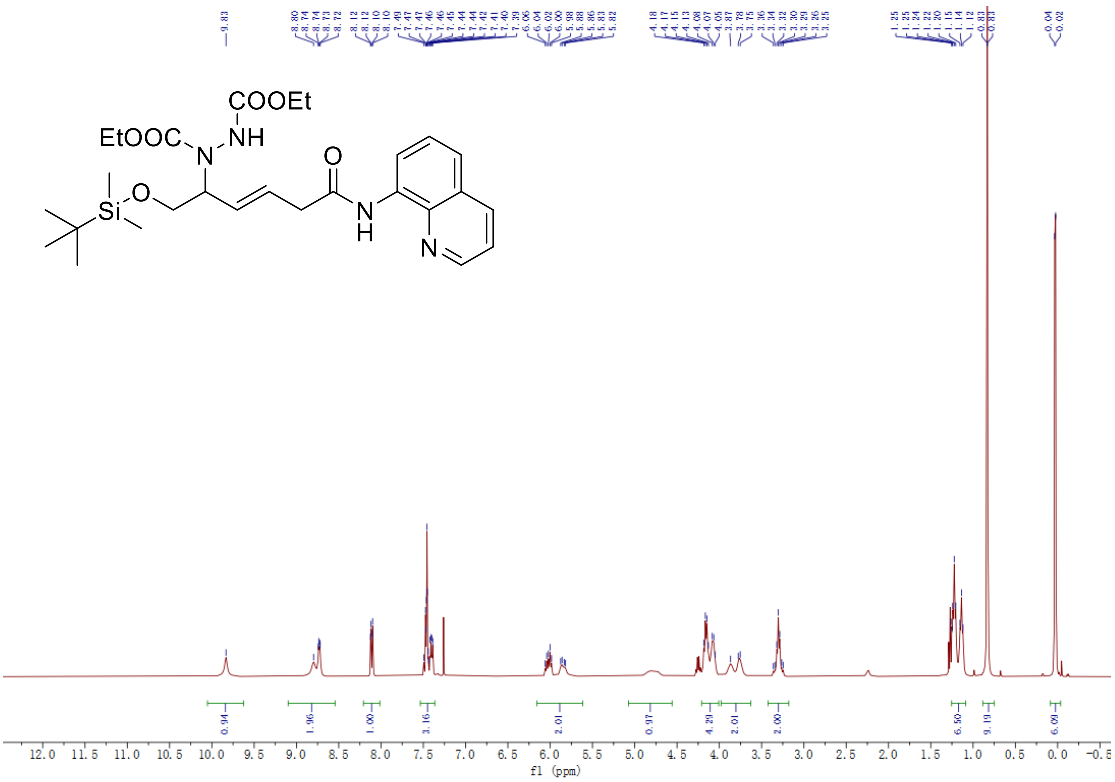

Supplementary Figure 49 ^{13}**C NMR**-spectrum (101 MHz, $CDCl_3$) of **26**

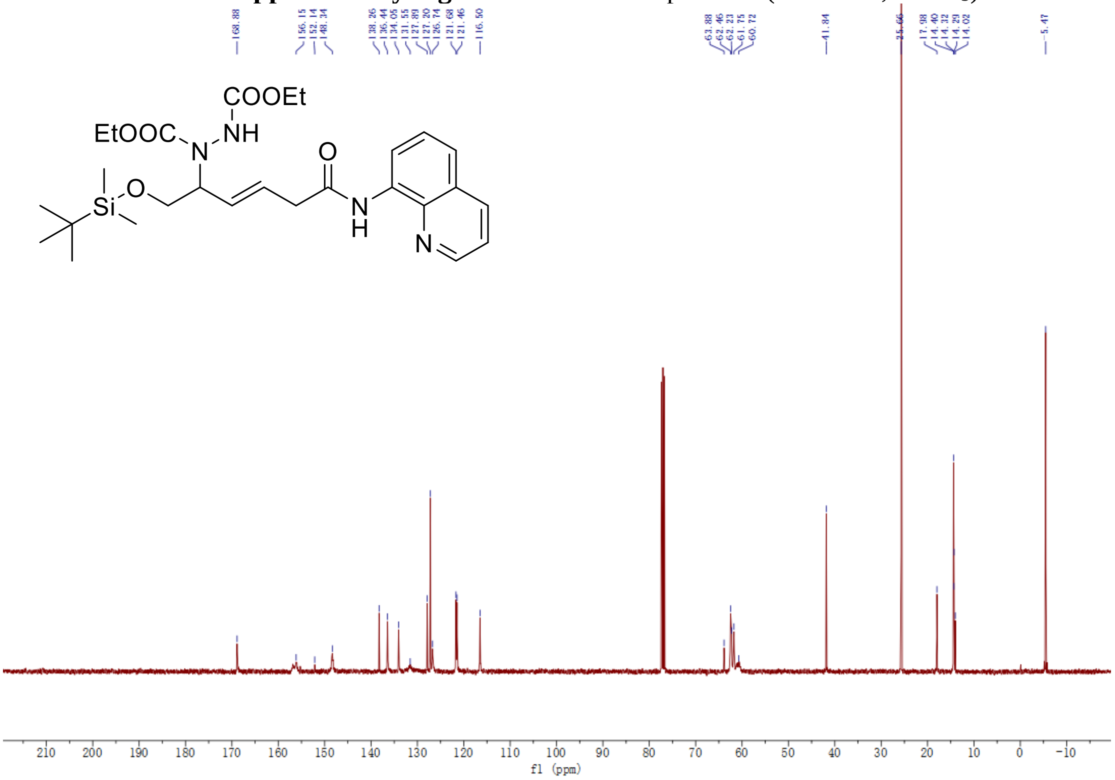

Supplementary Figure 50 ^{1}**H NMR**-spectrum (400 MHz, $CDCl_3$) of **27**

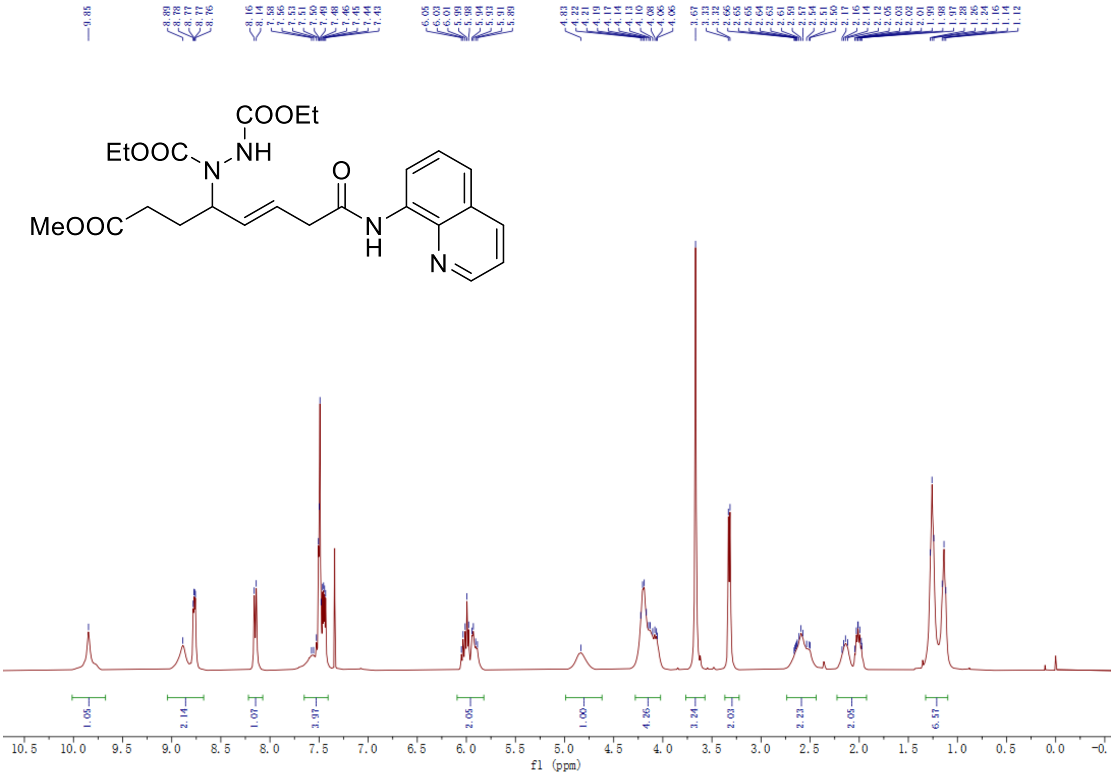

Supplementary Figure 51 ^{13}**C NMR**-spectrum (101 MHz, $CDCl_3$) of **27**

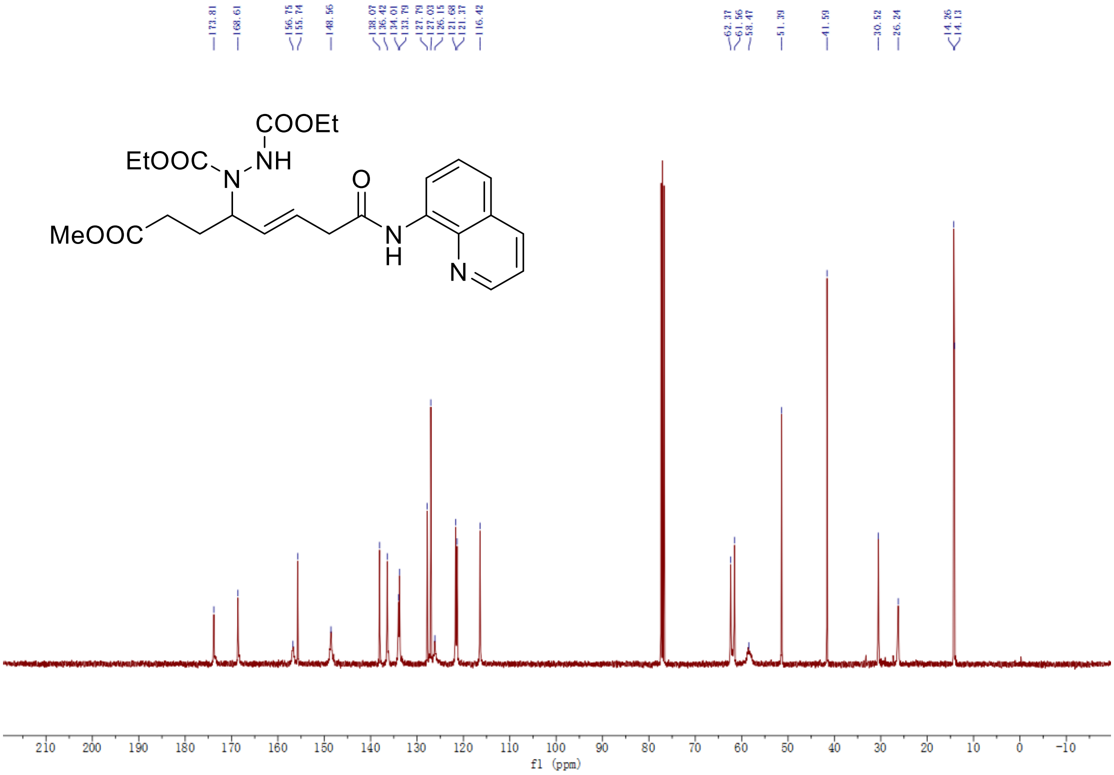

Supplementary Figure 52 **^{1}H NMR**-spectrum (400 MHz, $CDCl_3$) of **28**

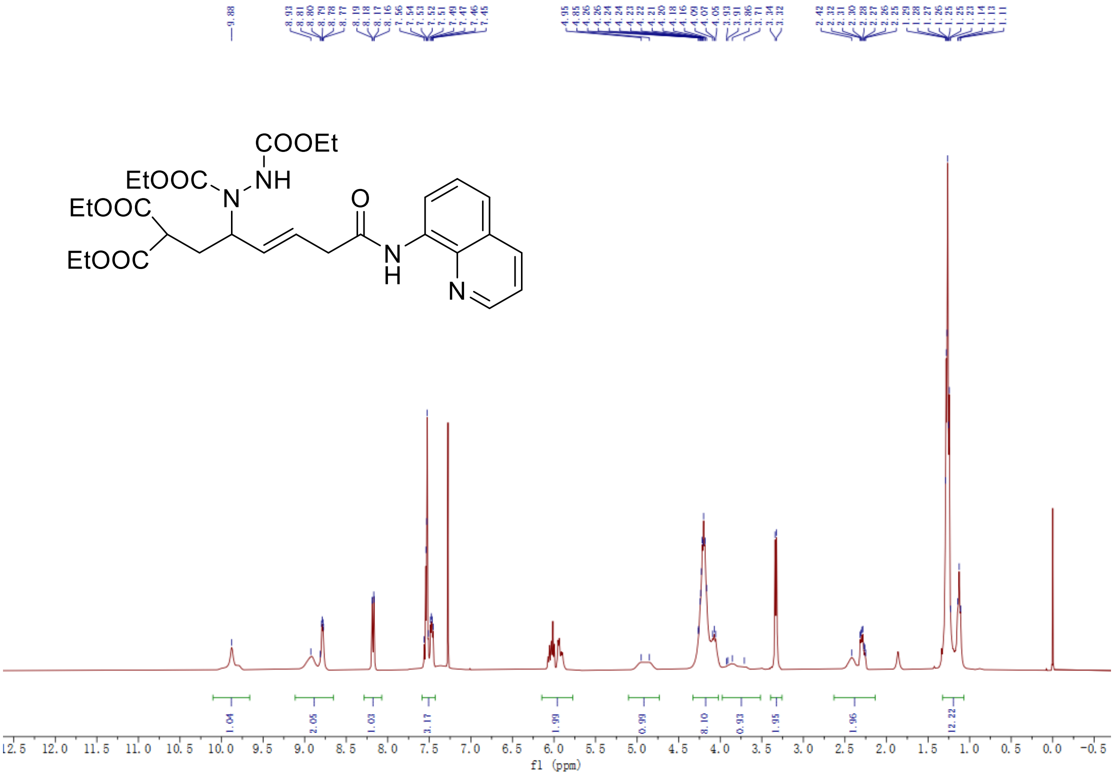

Supplementary Figure 53 **^{13}C NMR-**spectrum (101 MHz, $CDCl_3$) of **28**

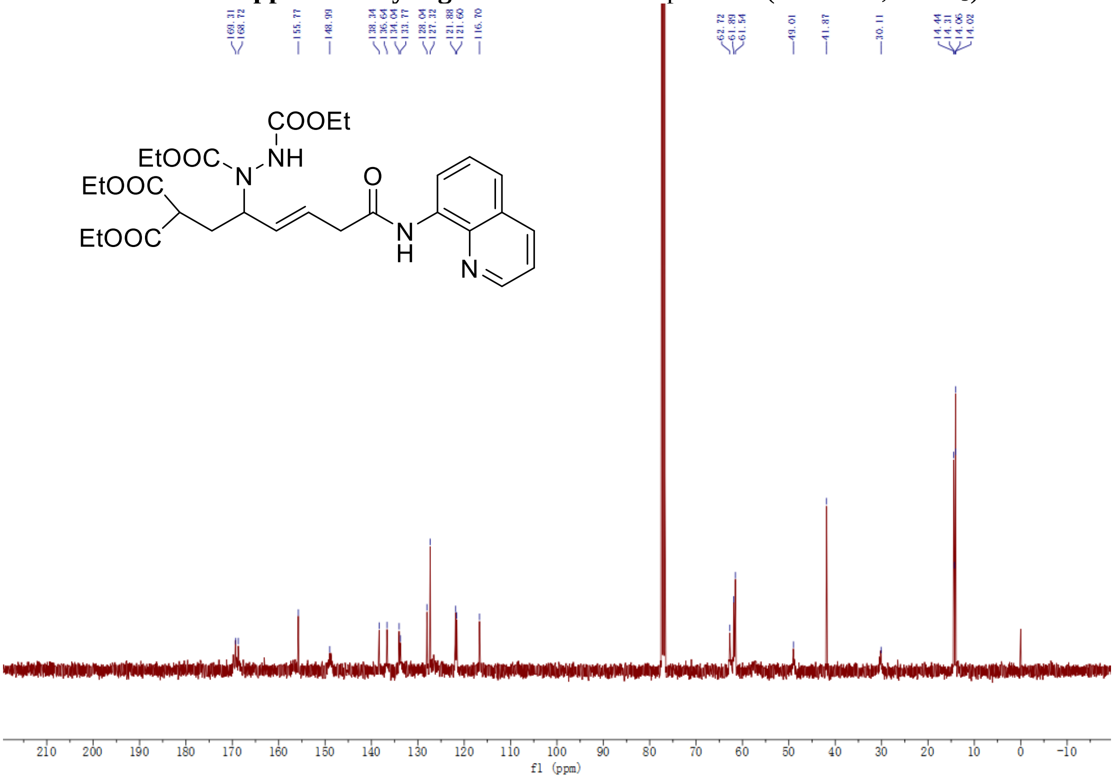

Supplementary Figure 54 ^{1}**H NMR**-spectrum (400 MHz, $CDCl_3$) of **29**

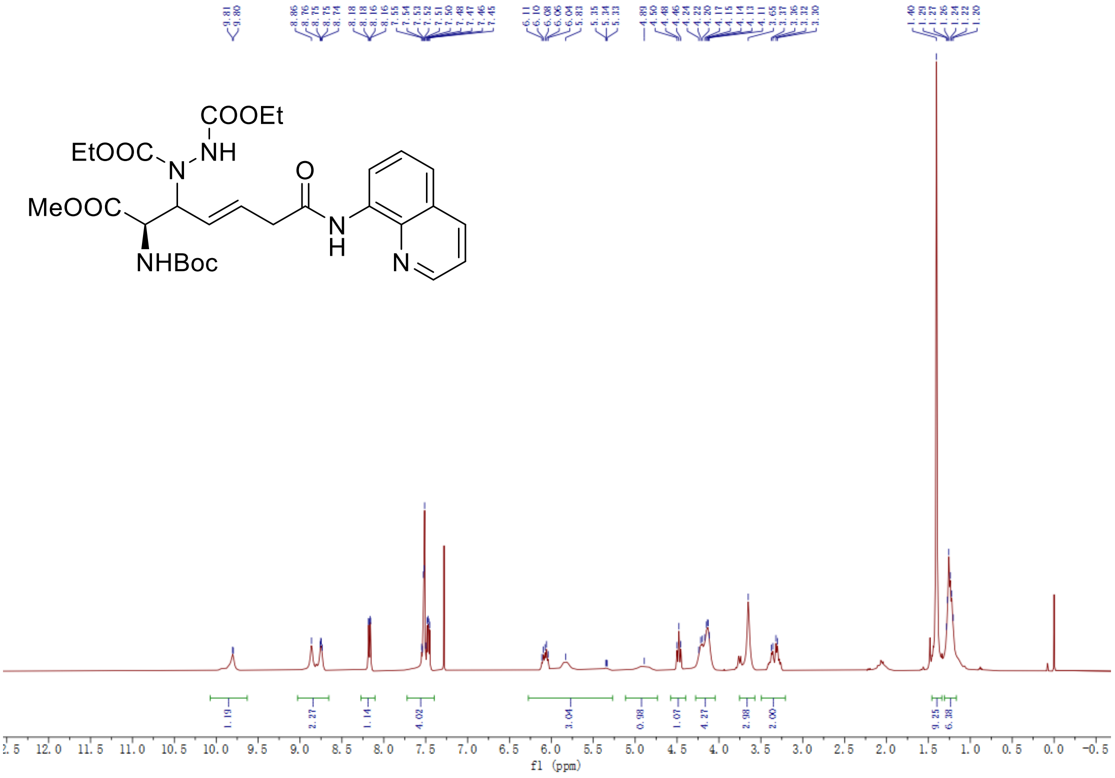

Supplementary Figure 55 ^{13}**C NMR**-spectrum (101 MHz, $CDCl_3$) of **29**

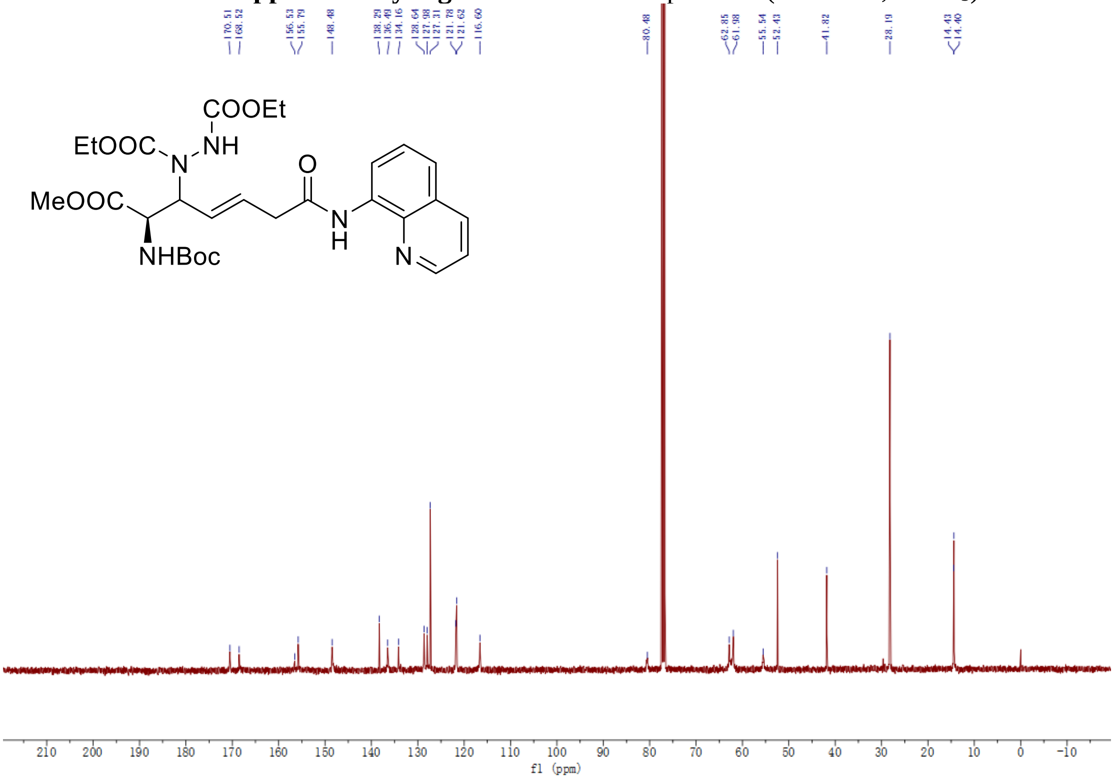

Supplementary Figure 56 ^{1}H NMR-spectrum (500 MHz, $CDCl_3$) of **30**

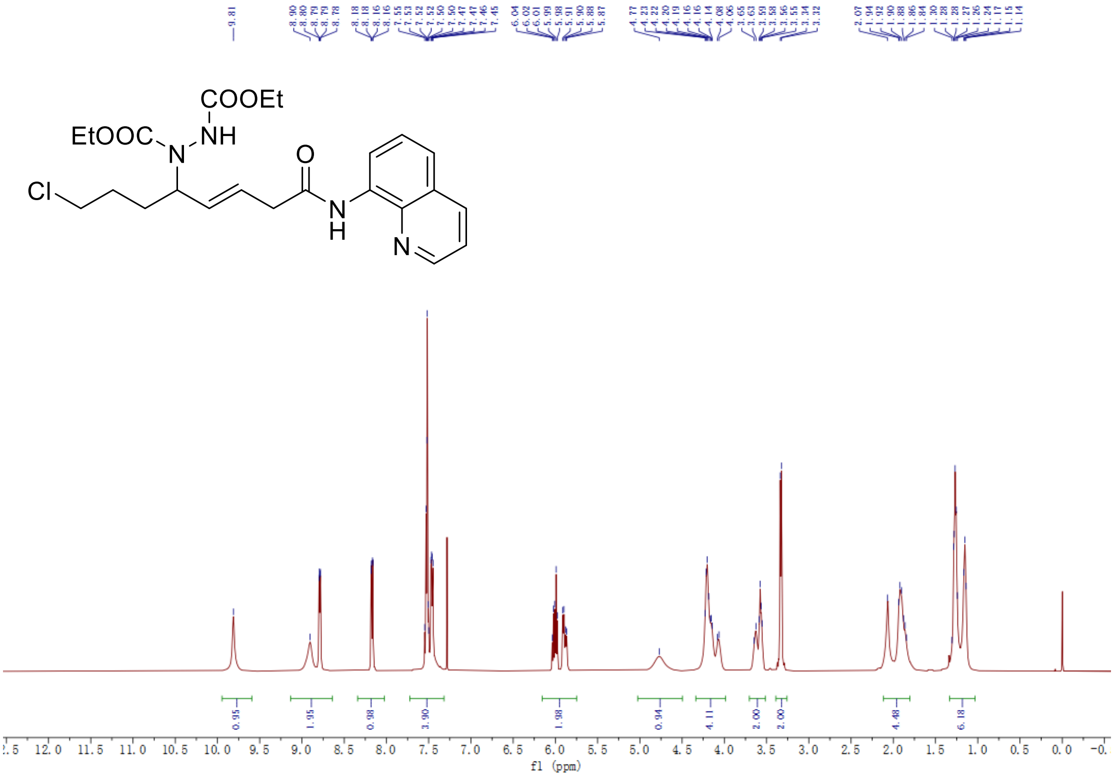

Supplementary Figure 57 ^{13}C NMR-spectrum (101 MHz, $CDCl_3$) of **30**

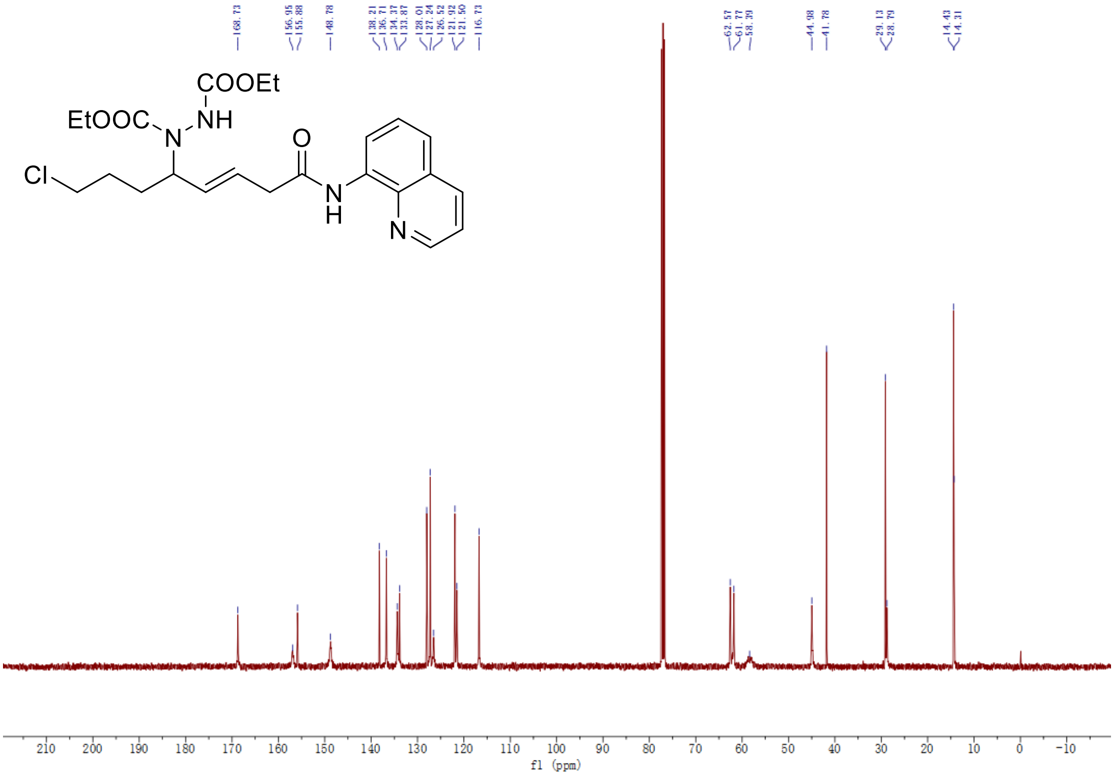

Supplementary Figure 58 ^{1}**H NMR**-spectrum (400 MHz, $CDCl_3$) of **31**

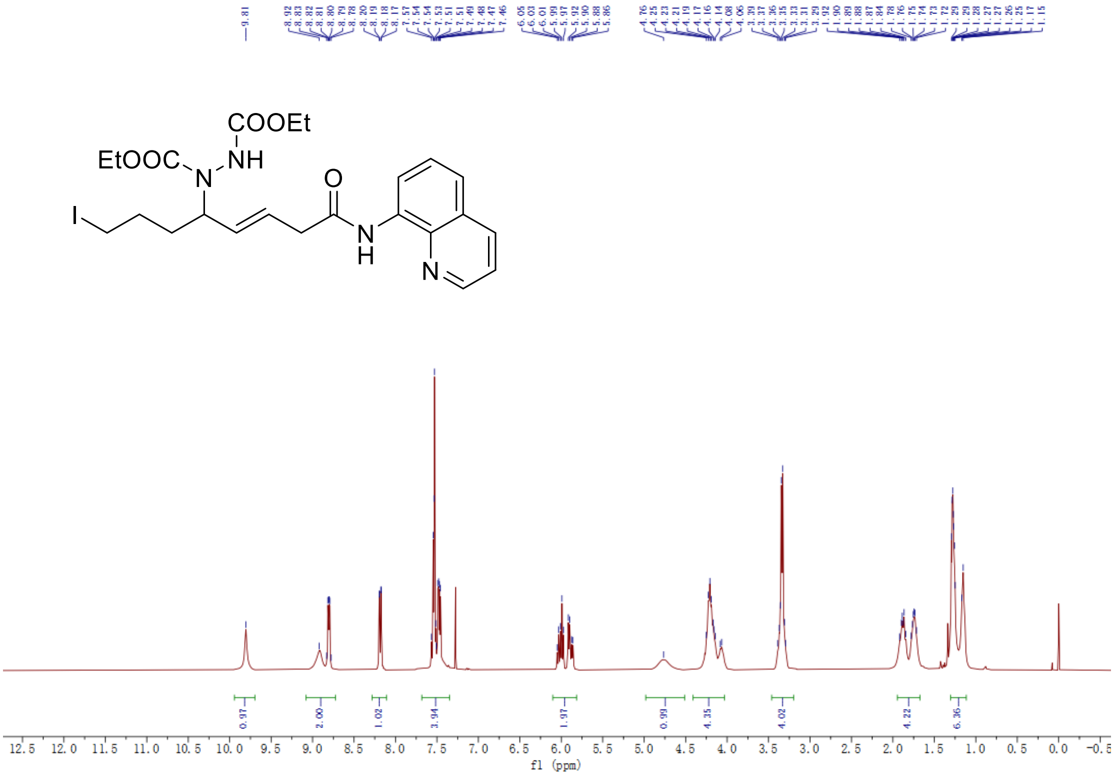

Supplementary Figure 59 ^{13}**C NMR**-spectrum (101 MHz, $CDCl_3$) of **31**

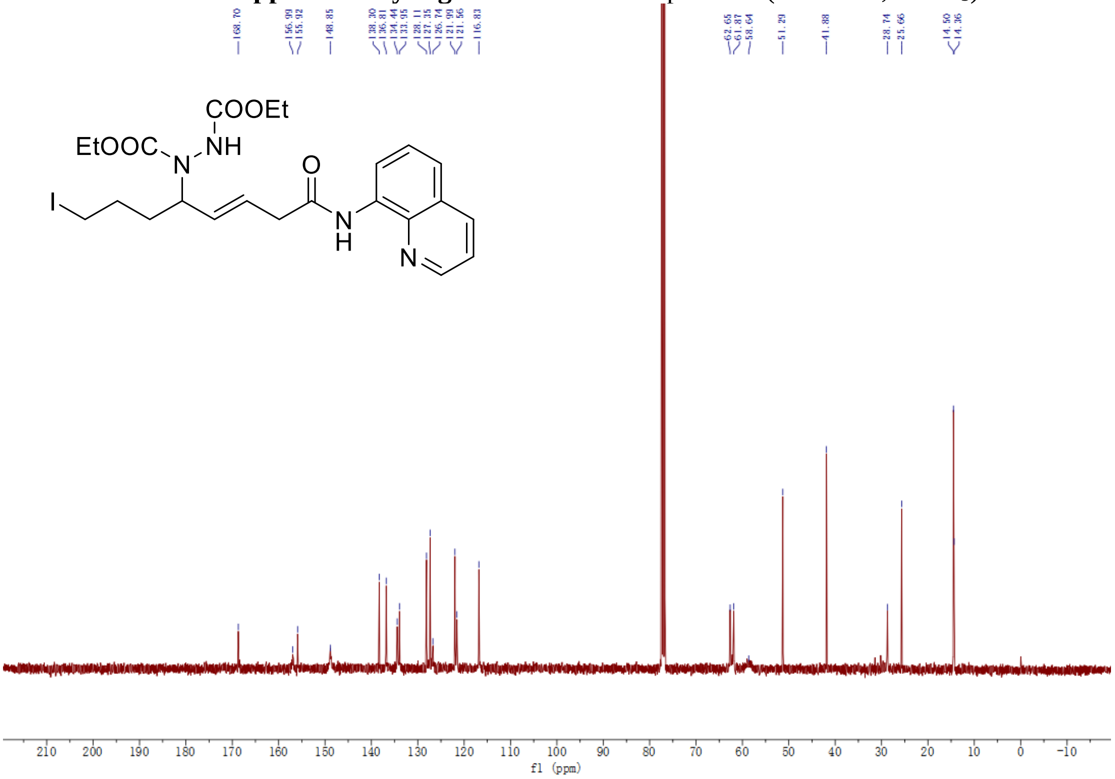

Supplementary Figure 60 ^{1}**H NMR**-spectrum (400 MHz, $CDCl_3$) of **32**

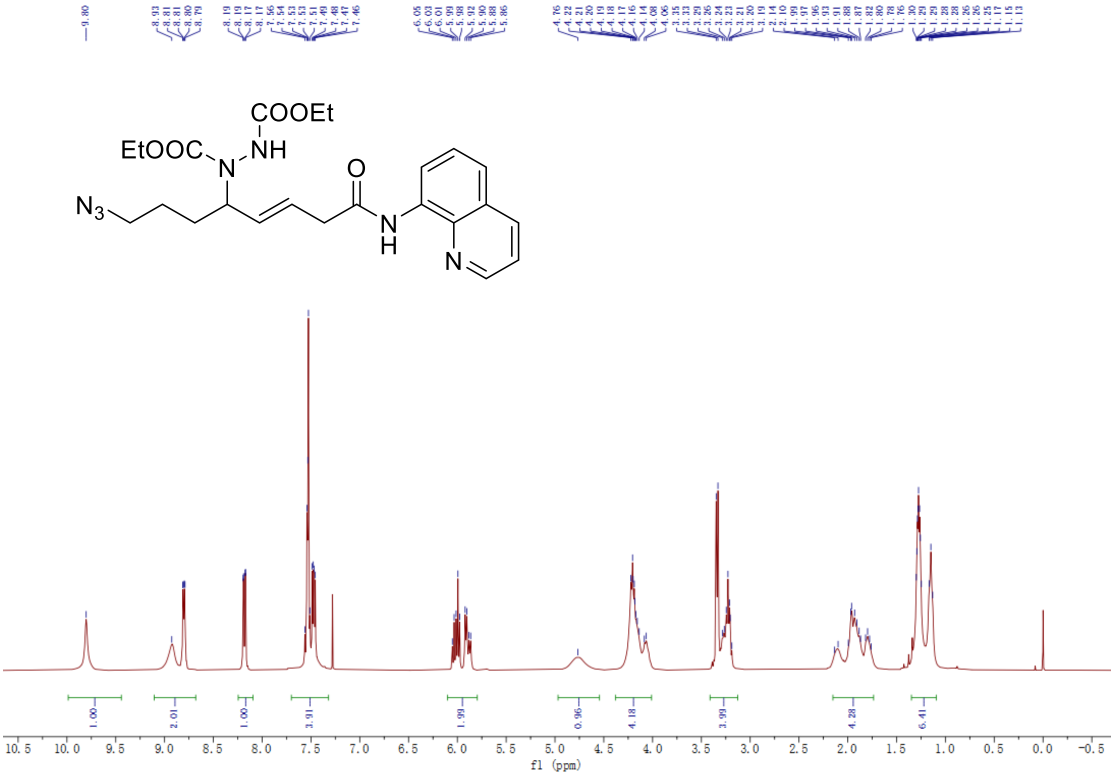

Supplementary Figure 61 ^{13}**C NMR**-spectrum (101 MHz, $CDCl_3$) of **32**

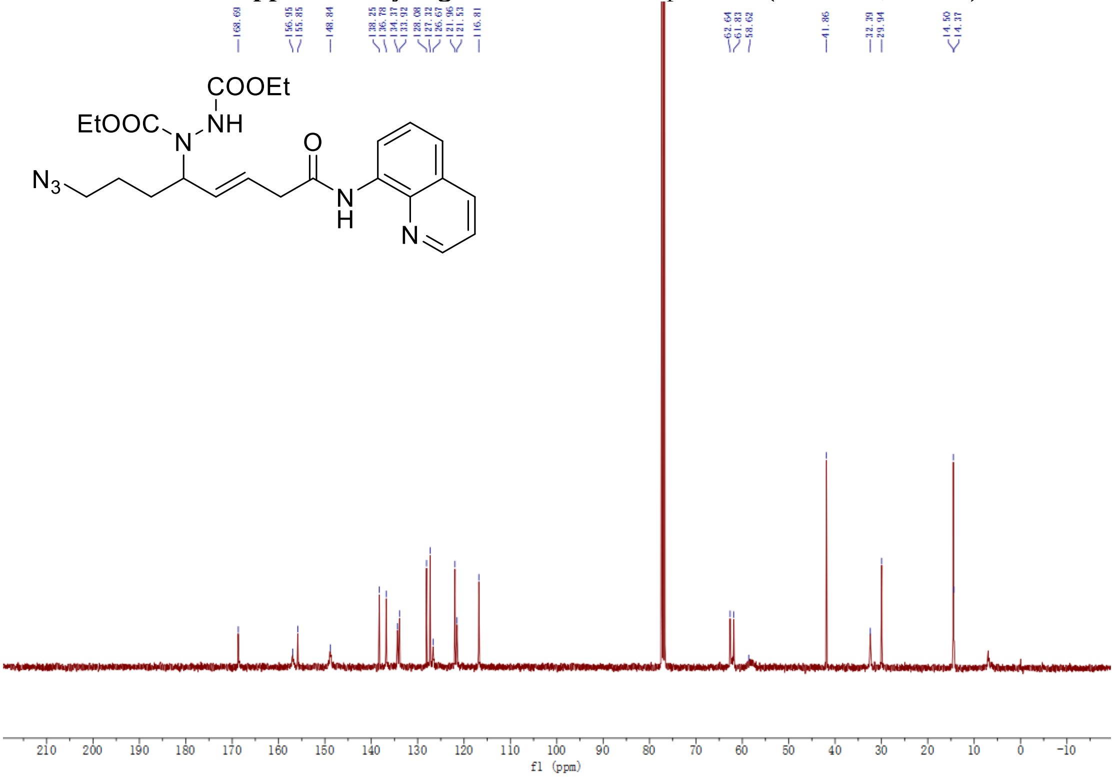

Supplementary Figure 62 ^{1}**H NMR**-spectrum (400 MHz, $CDCl_3$) of **33**

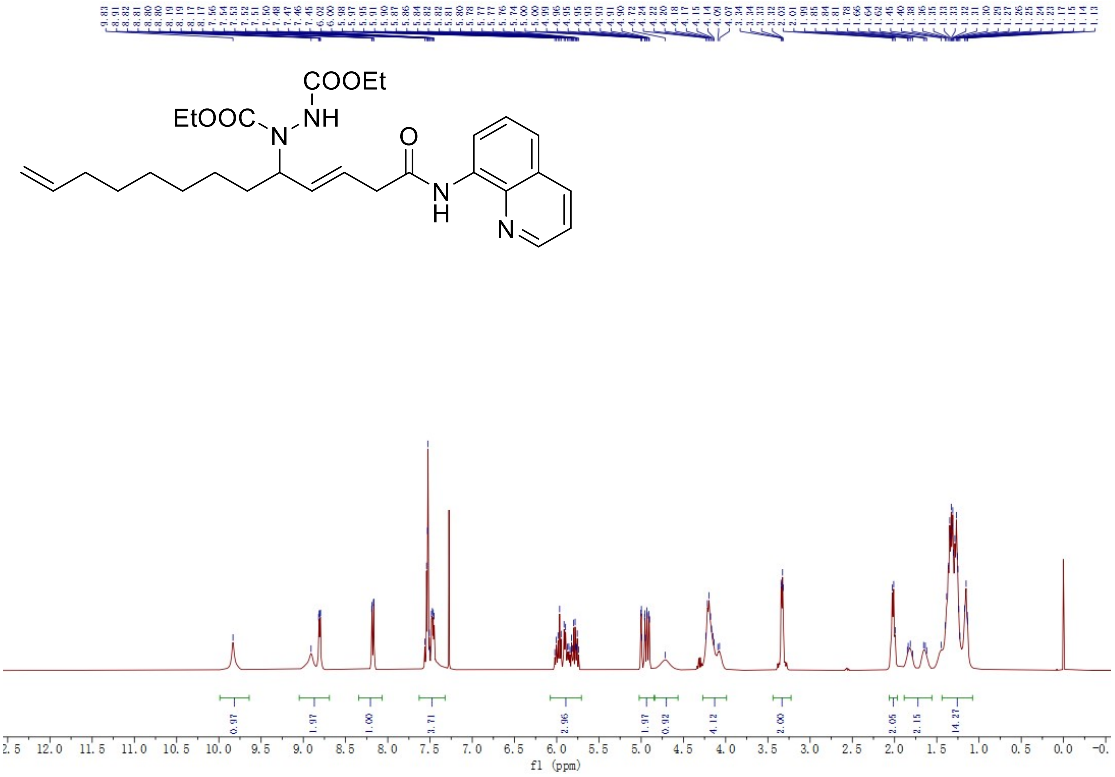

Supplementary Figure 63 ^{13}**C NMR**-spectrum (101 MHz, $CDCl_3$) of **33**

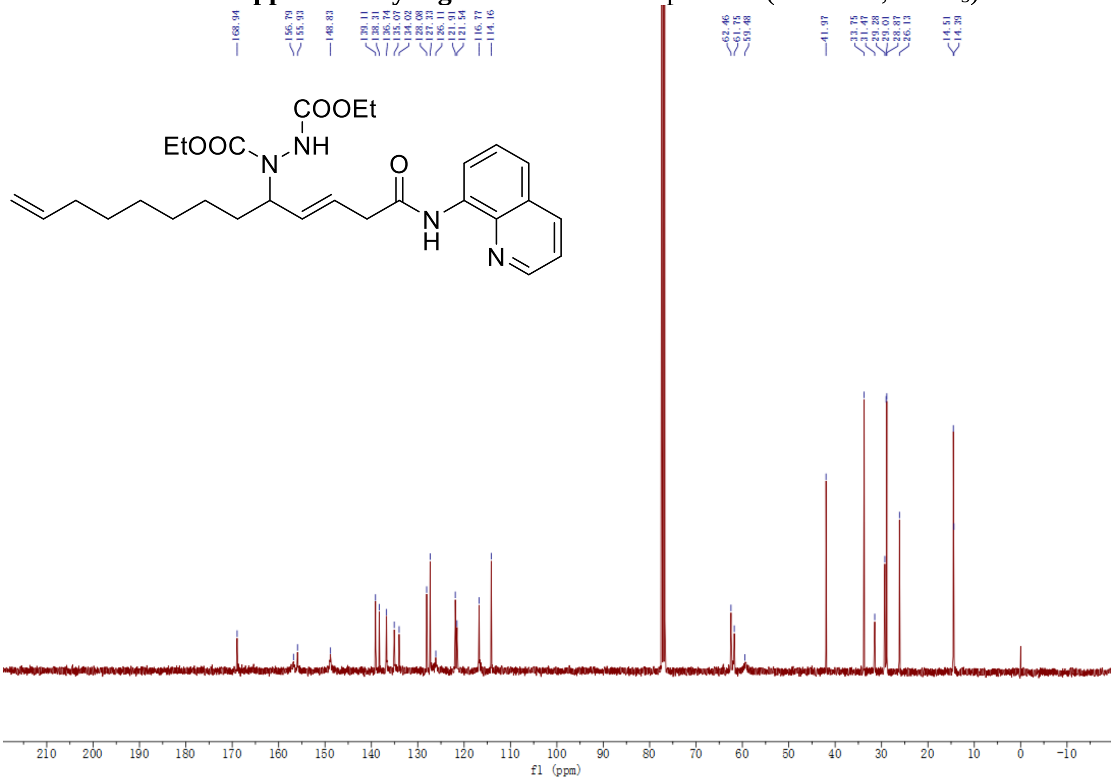

Supplementary Figure 64 ^{1}**H NMR**-spectrum (400 MHz, $CDCl_3$) of **34**

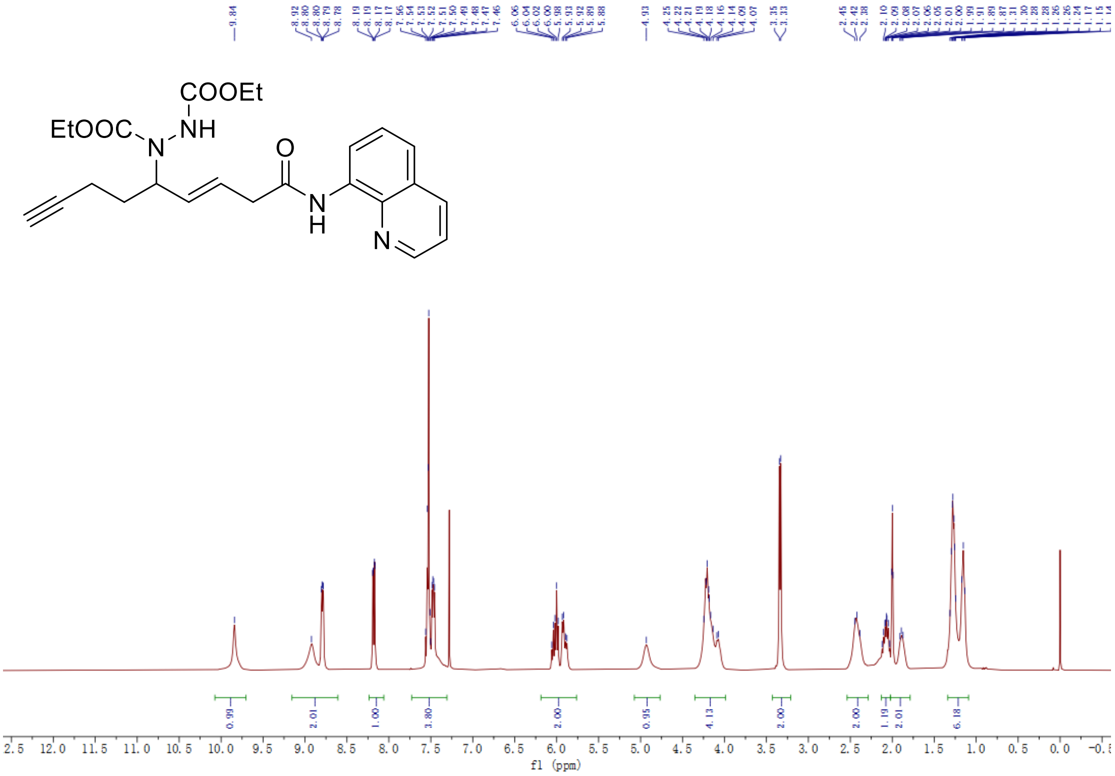

Supplementary Figure 65 ^{13}**C NMR**-spectrum (101 MHz, $CDCl_3$) of **34**

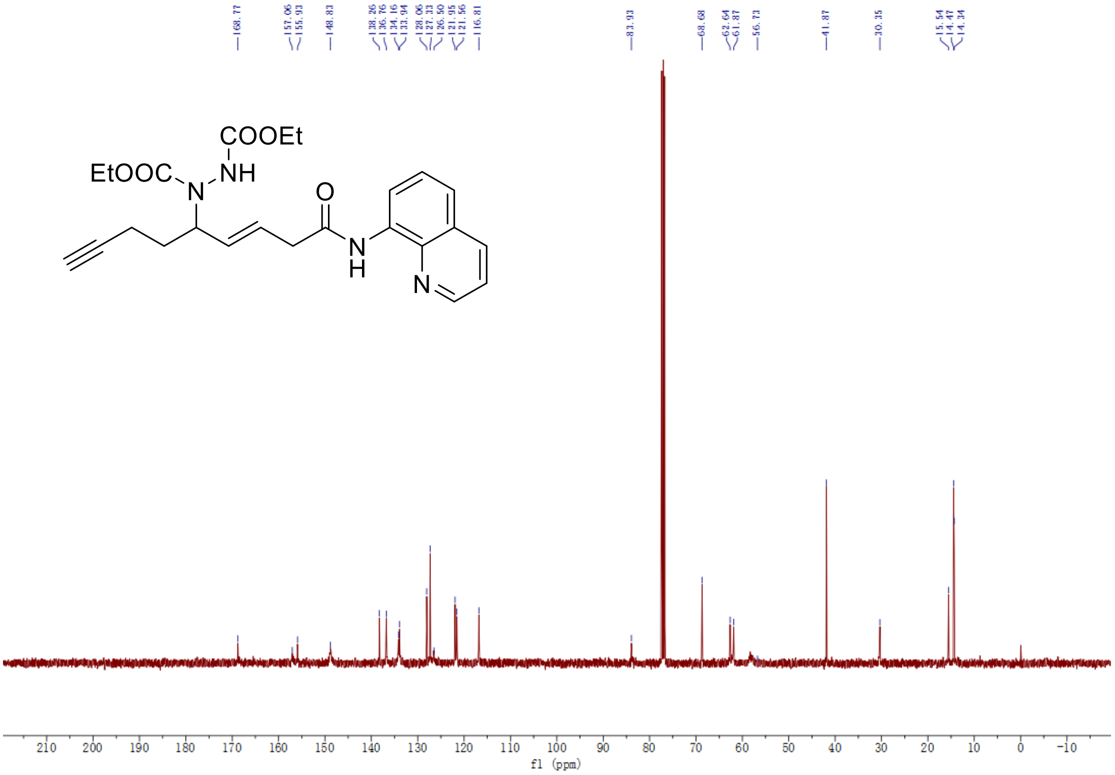

Supplementary Figure 66 ^{1}H NMR-spectrum (400 MHz, $CDCl_3$) of **35**

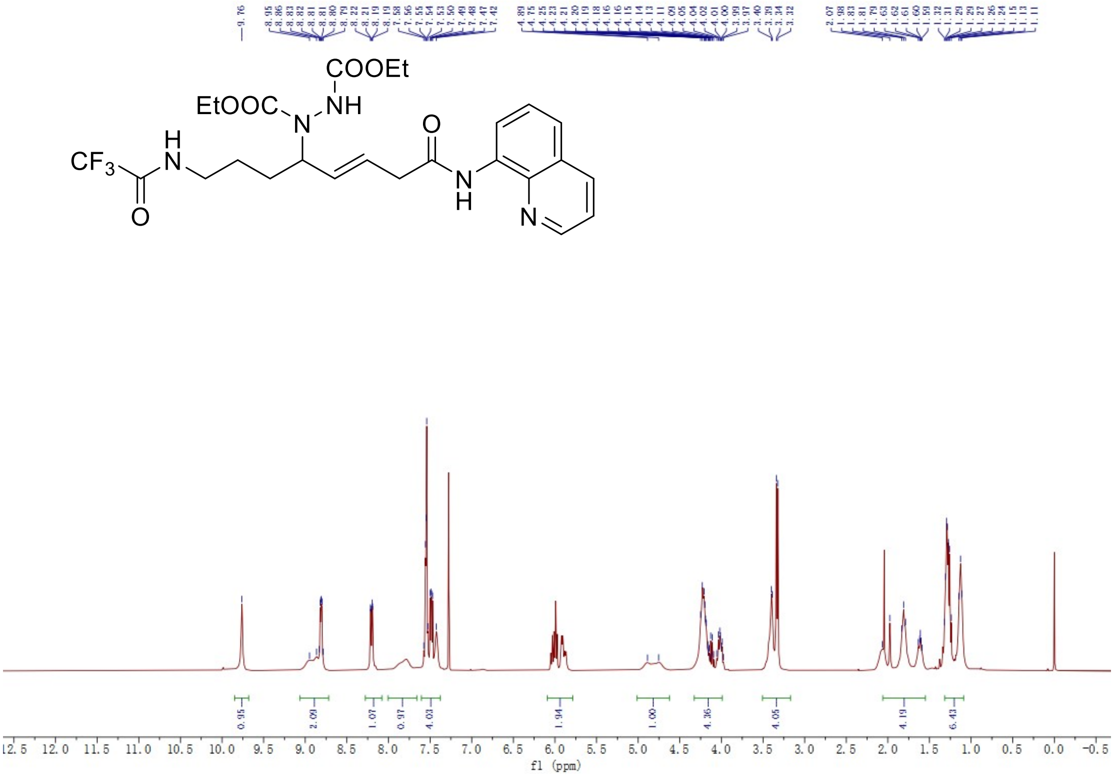

Supplementary Figure 67 ^{13}C NMR-spectrum (101 MHz, $CDCl_3$) of **35**

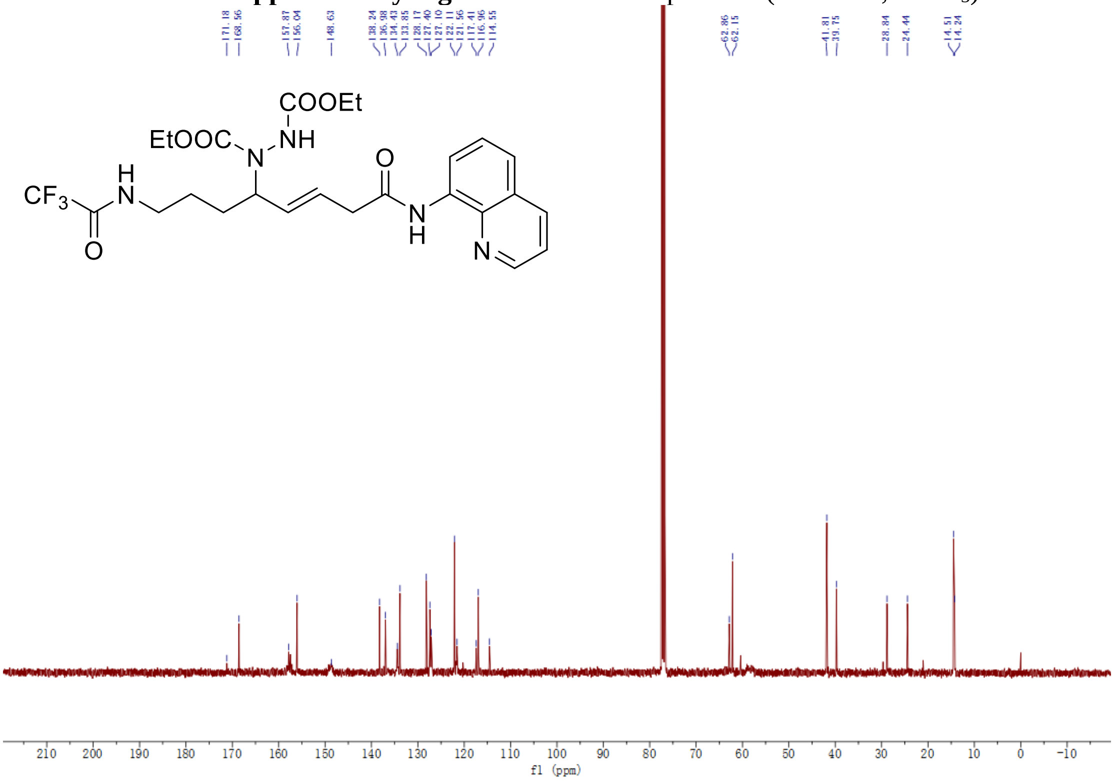

Supplementary Figure 68 ^{19}**F NMR**-spectrum (376 MHz, $CDCl_3$) of **35**

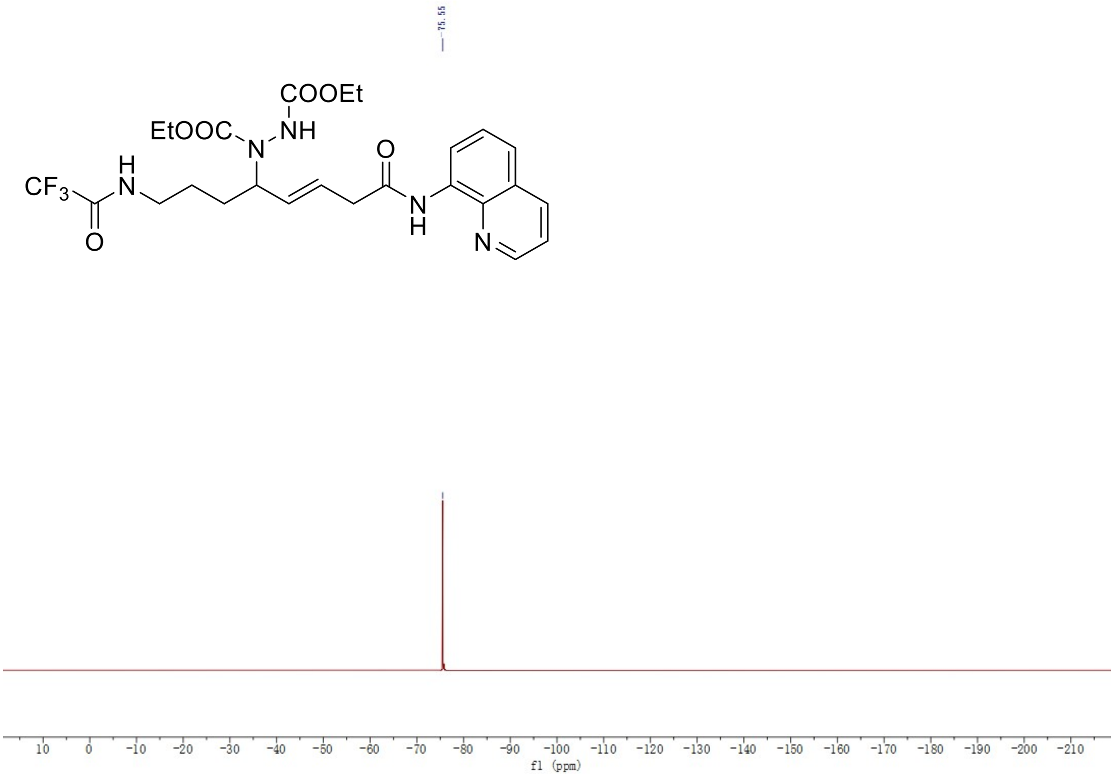

Supplementary Figure 69 ^{1}**H NMR**-spectrum (400 MHz, $CDCl_3$) of **36**

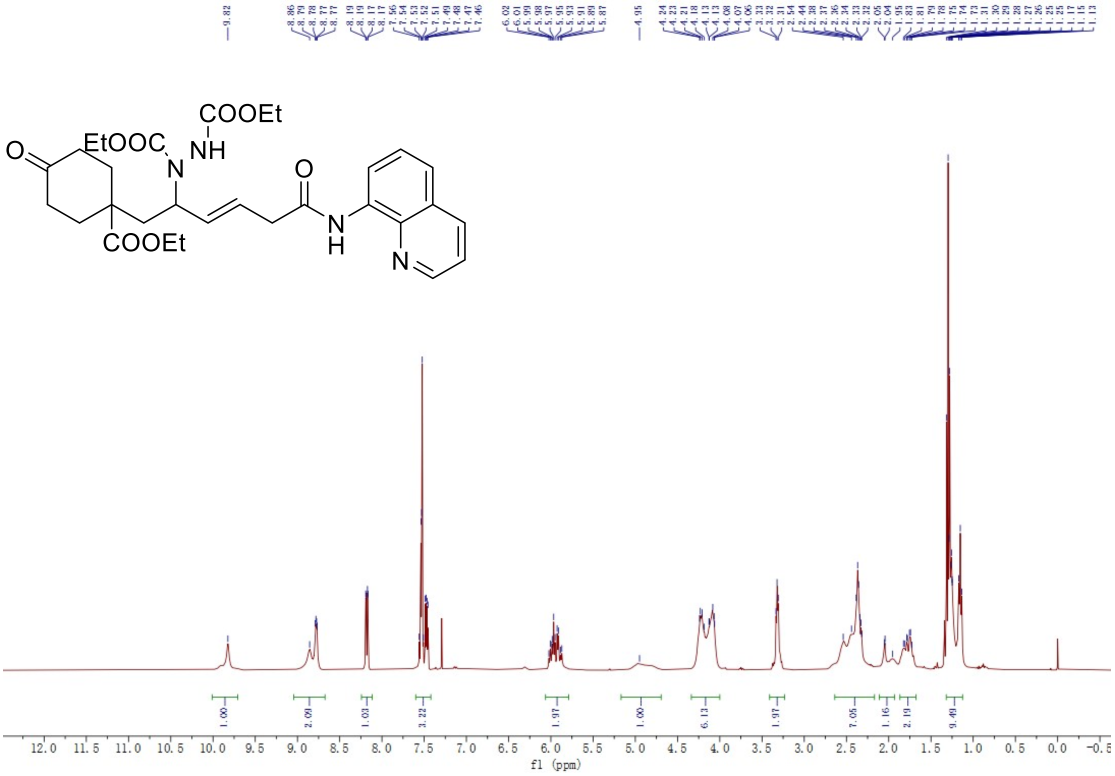

Supplementary Figure 70 ^{13}**C NMR**-spectrum (101 MHz, $CDCl_3$) of **36**

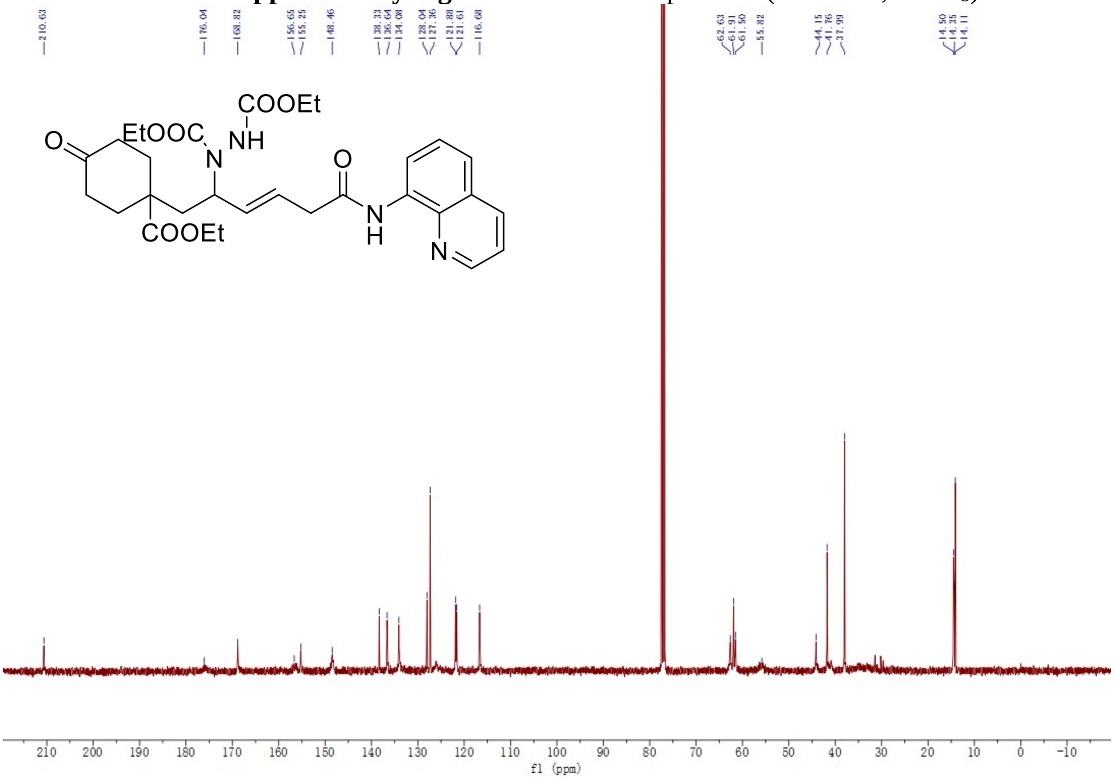

Supplementary Figure 71 ^{1}**H NMR**-spectrum (500 MHz, $CDCl_3$) of **37**

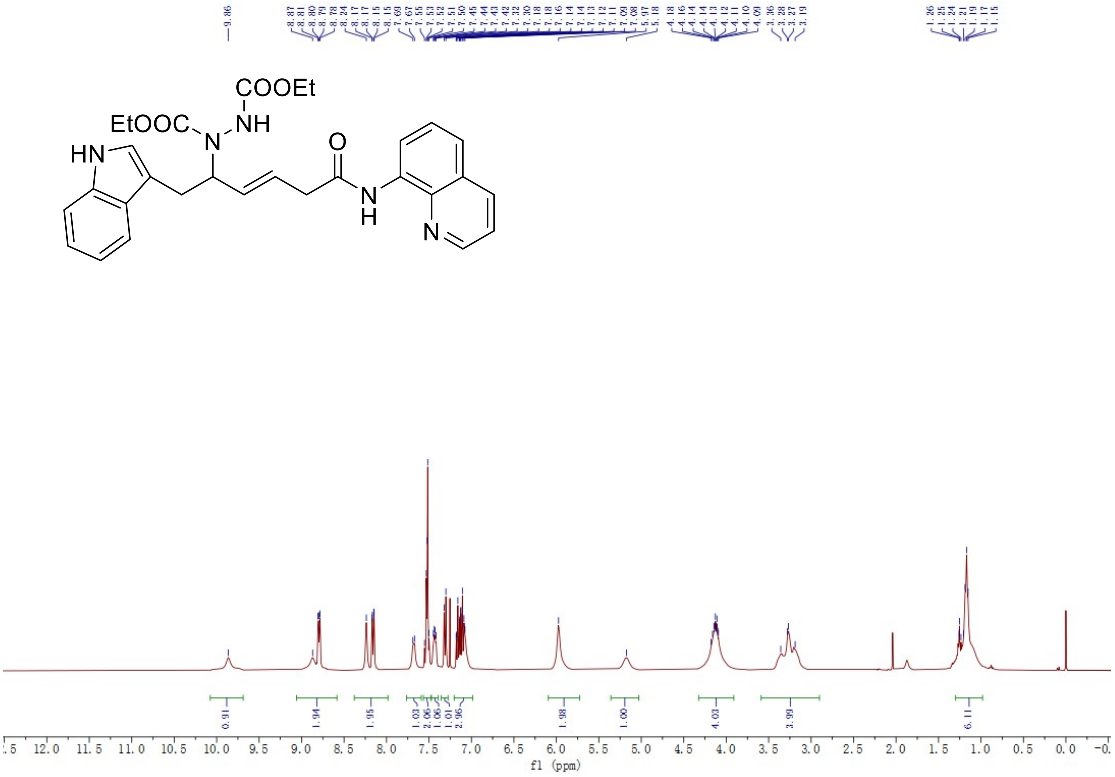

Supplementary Figure 72 ^{13}**C NMR**-spectrum (126 MHz, $CDCl_3$) of **37**

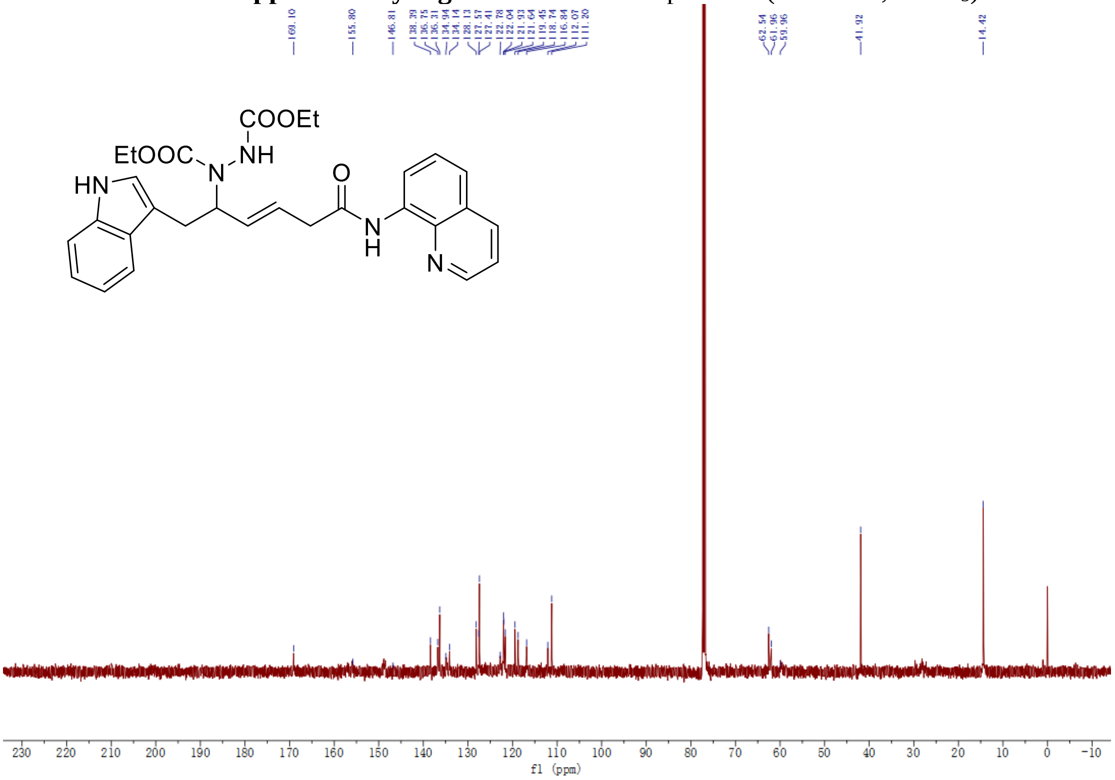

Supplementary Figure 73 ^{1}**H NMR**-spectrum (400 MHz, $CDCl_3$) of **38**

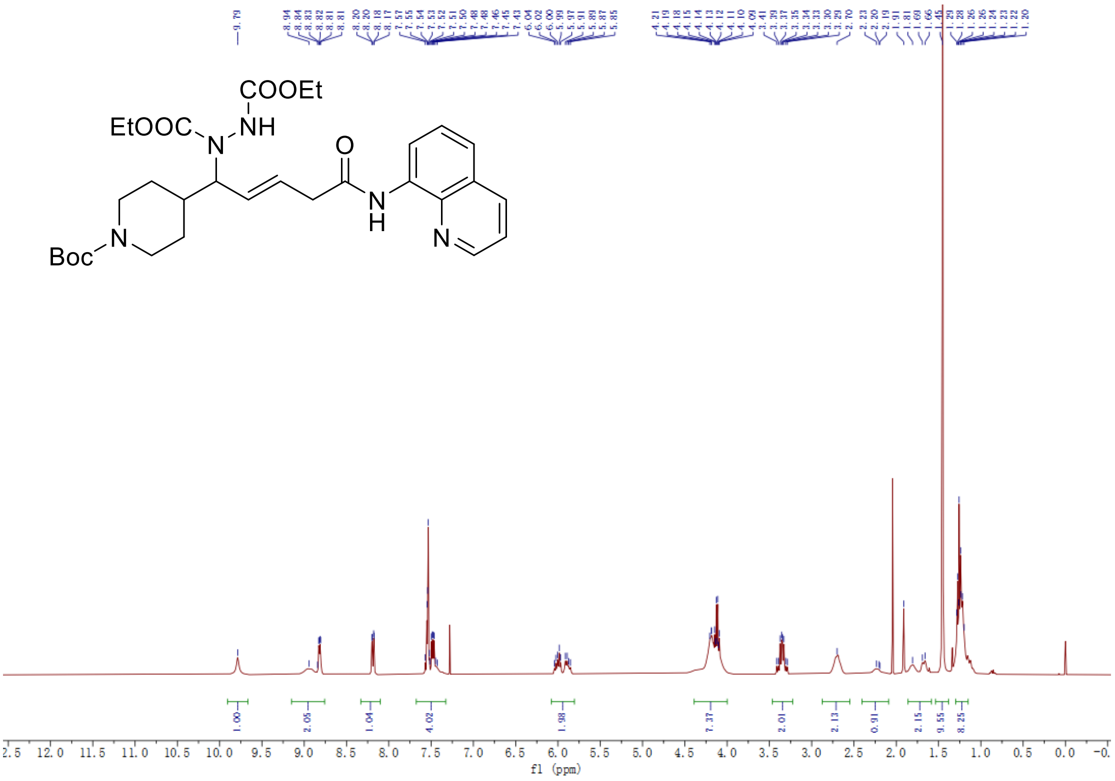

Supplementary Figure 74 ^{13}**C NMR**-spectrum (101 MHz, $CDCl_3$) of **38**

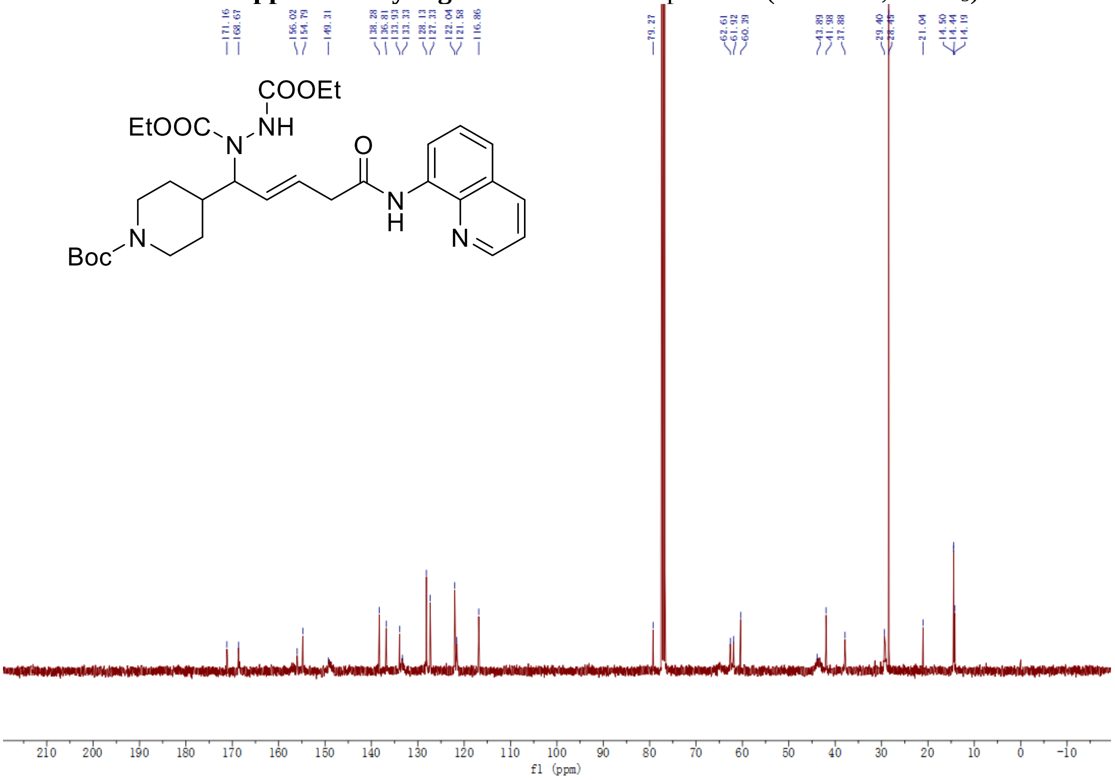

Supplementary Figure 75 ^{1}**H NMR**-spectrum (400 MHz, $CDCl_3$) of **39**

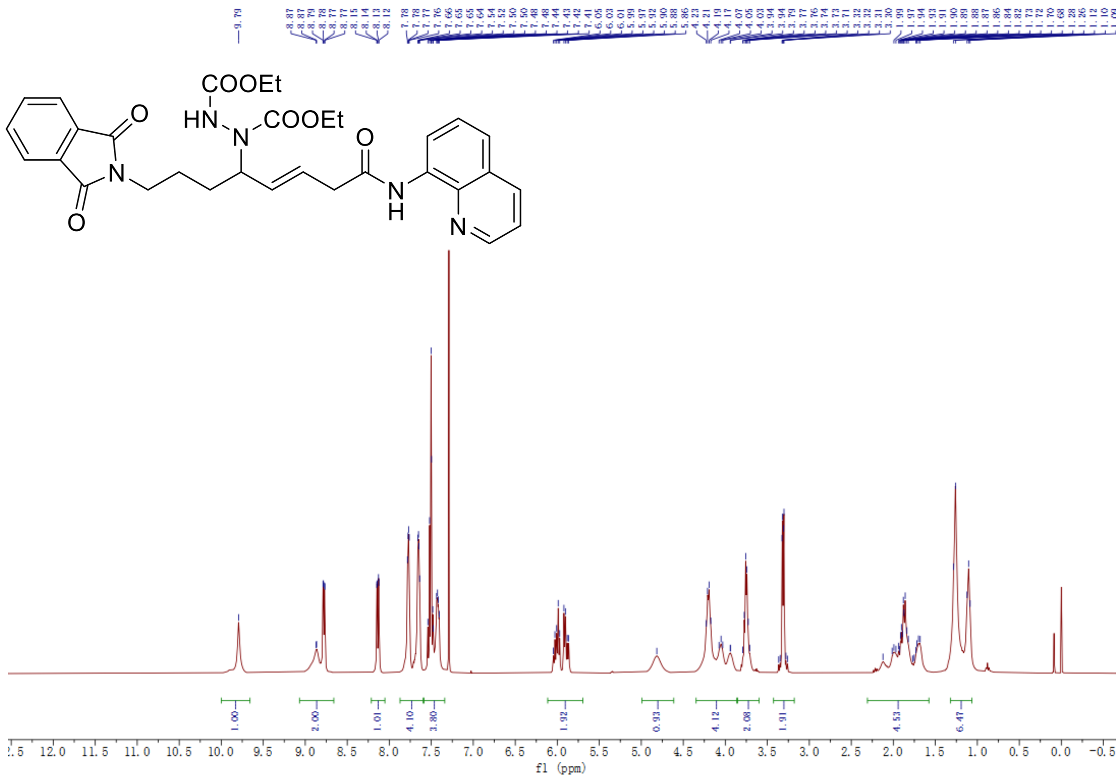

Supplementary Figure 76 ^{13}C NMR-spectrum (101 MHz, $CDCl_3$) of **39**

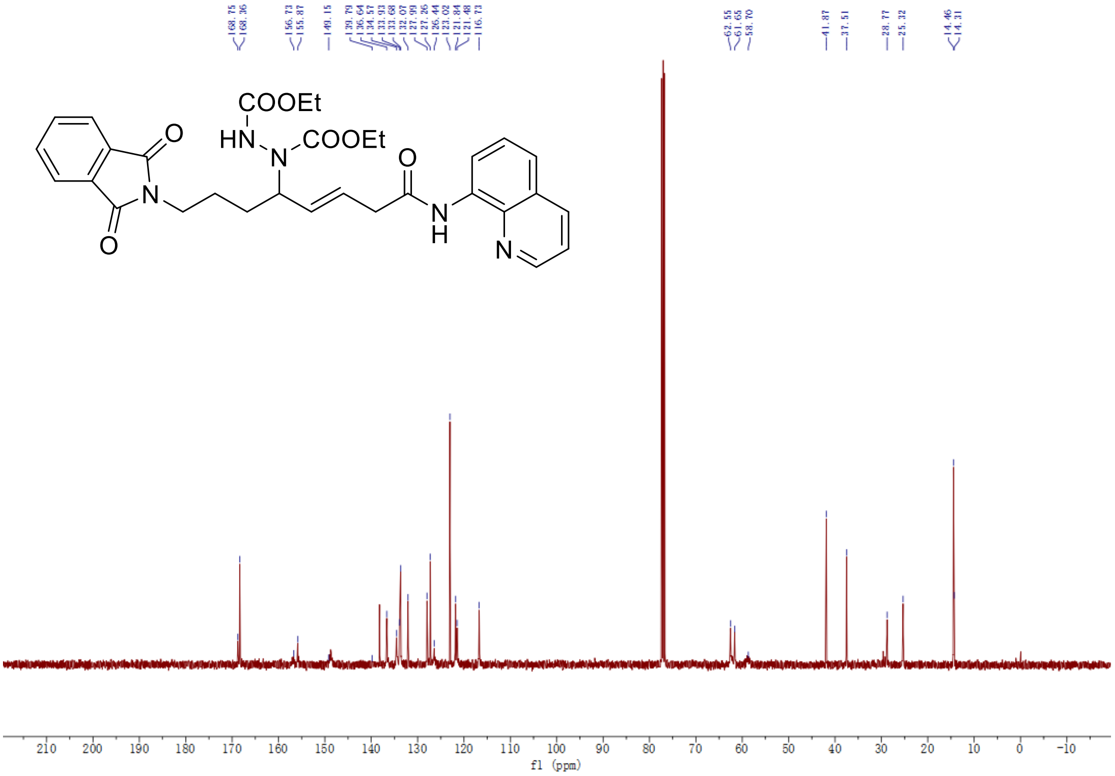

Supplementary Figure 77 ^{1}H NMR-spectrum (400 MHz, $CDCl_3$) of **40**

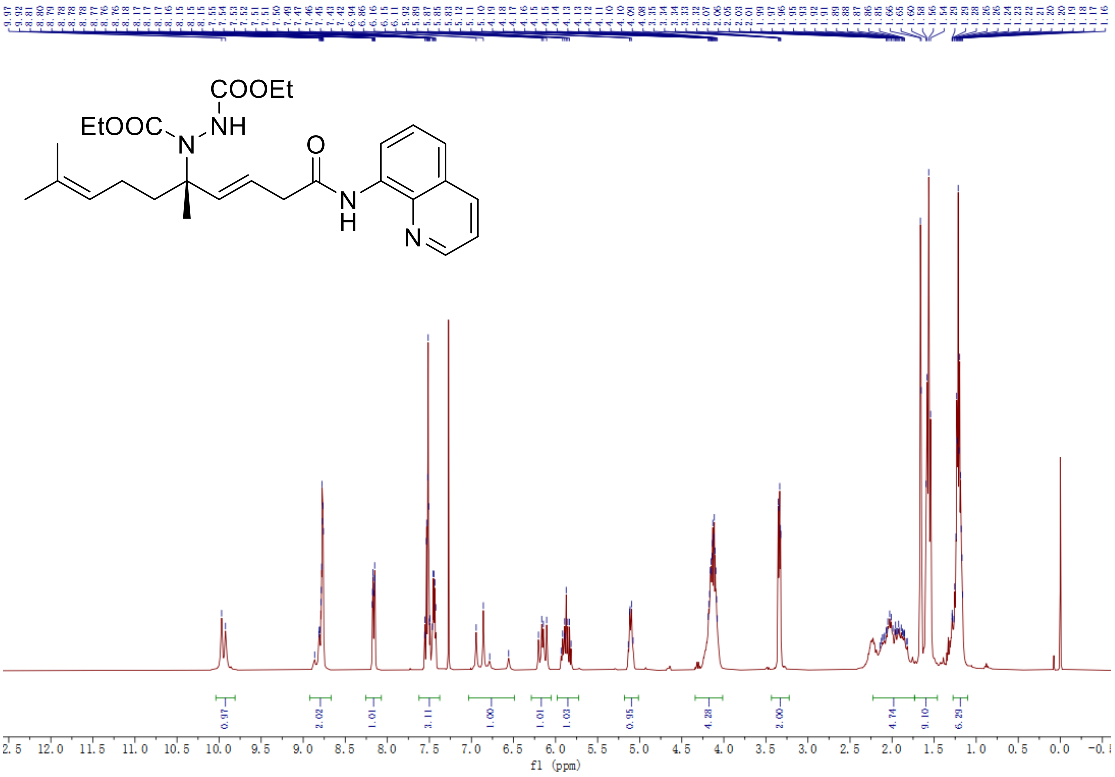

Supplementary Figure 78 ^{13}**C NMR**-spectrum (101 MHz, $CDCl_3$) of **40**

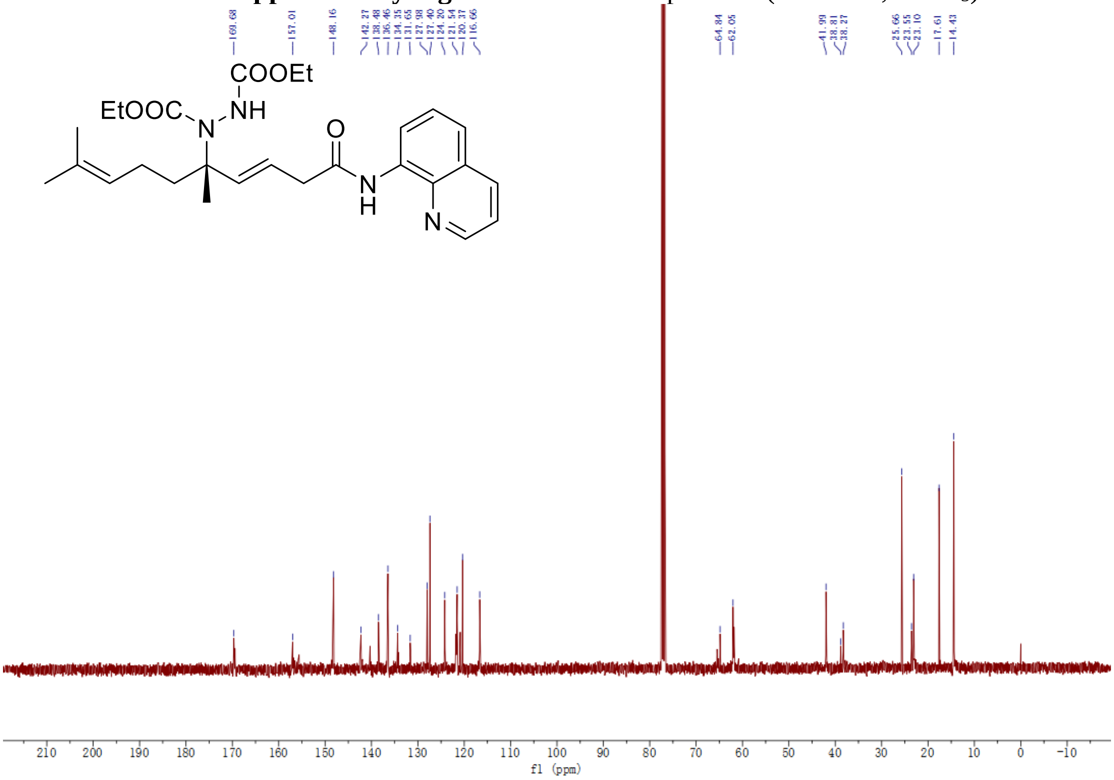

Supplementary Figure 79 ^{1}**H NMR**-spectrum (400 MHz, $CDCl_3$) of **41**

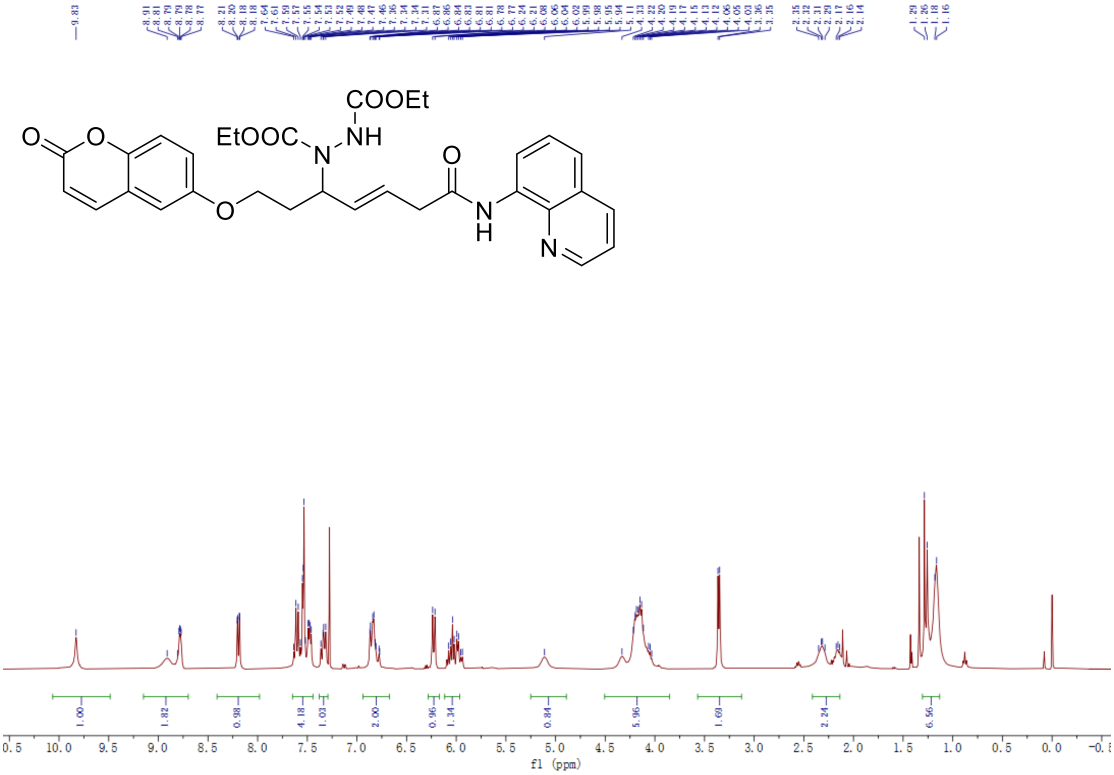

Supplementary Figure 80 ^{13}**C NMR**-spectrum (101 MHz, $CDCl_3$) of **41**

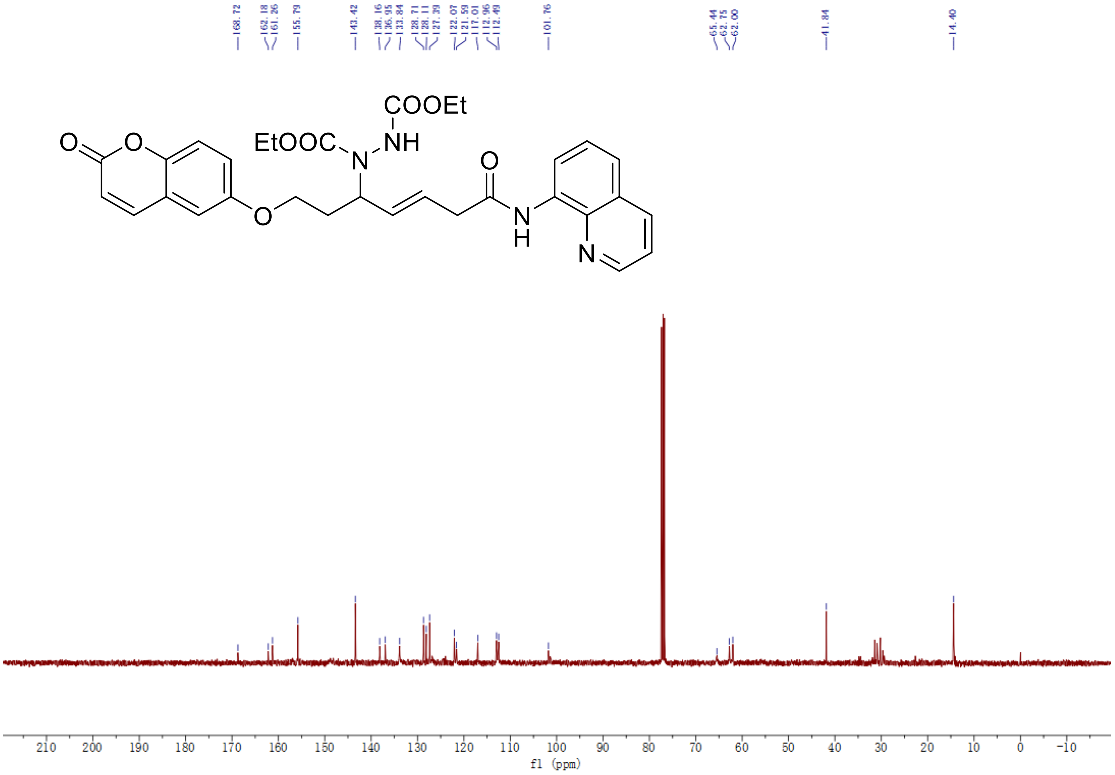

Supplementary Figure 81 ^{1}**H NMR**-spectrum (400 MHz, $CDCl_3$) of **42**

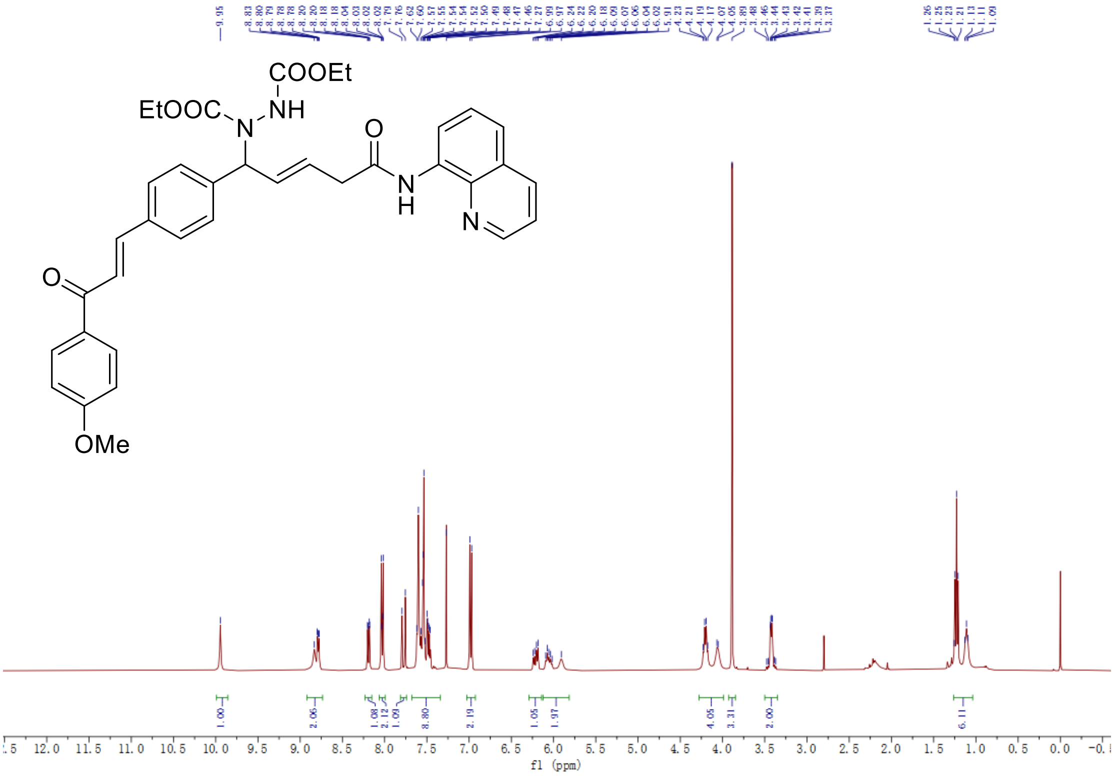

Supplementary Figure 82 ^{13}C **NMR**-spectrum (101 MHz, $CDCl_3$) of **42**

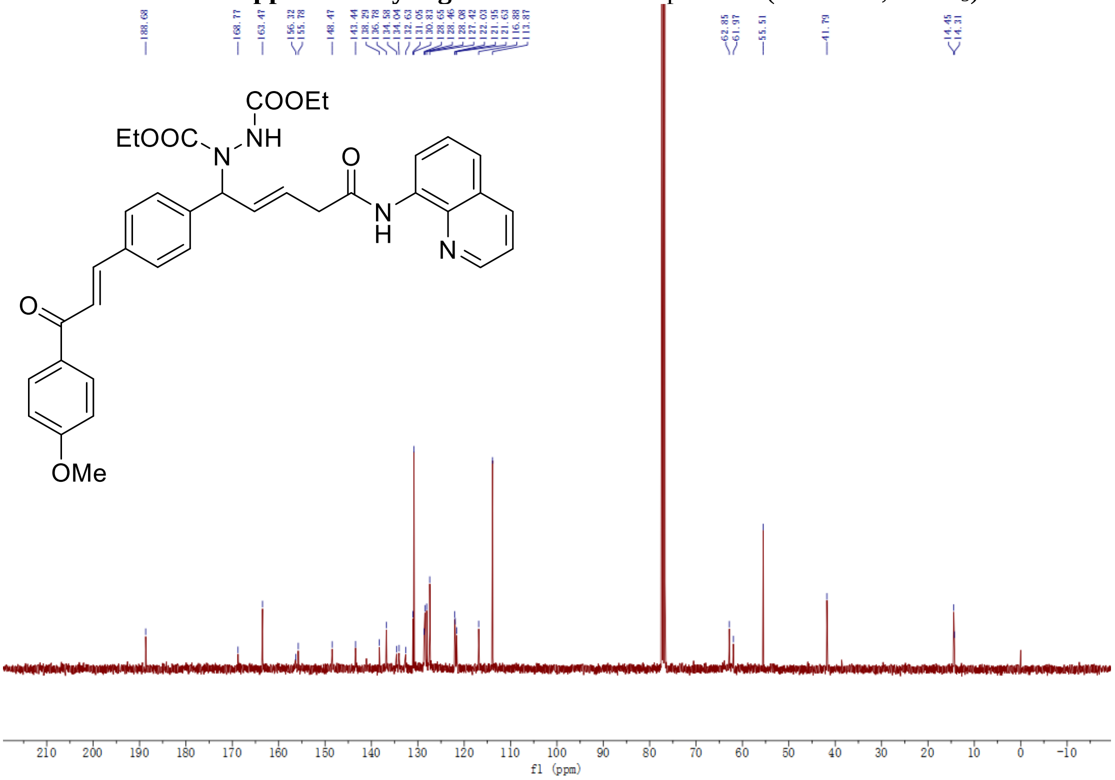

Supplementary Figure 83 ^{1}H **NMR**-spectrum (400 MHz, $CDCl_3$) of **43**

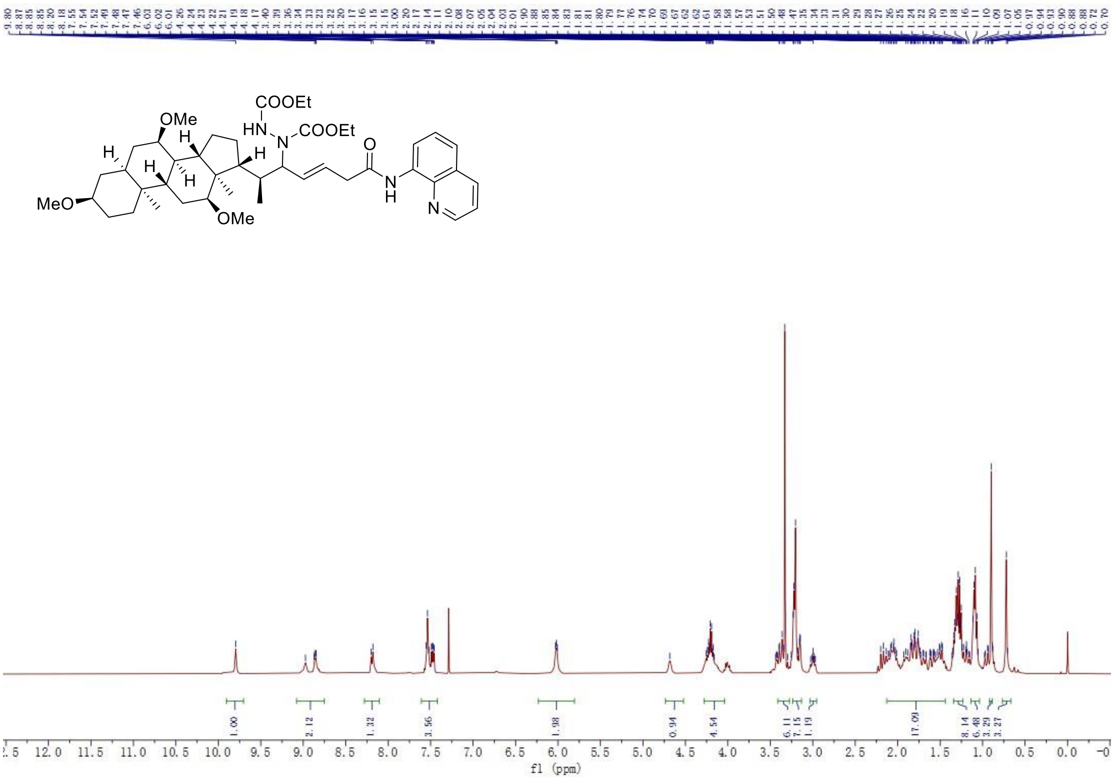

Supplementary Figure 84 ^{13}**C NMR**-spectrum (101 MHz, $CDCl_3$) of **43**

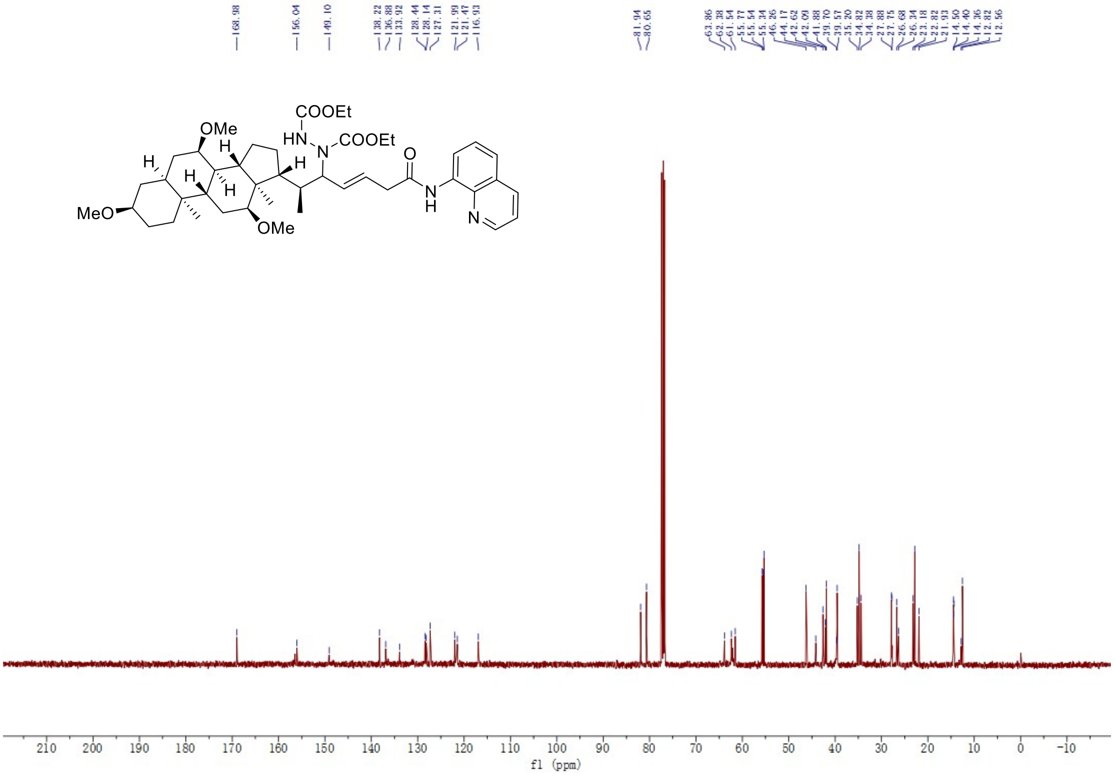

Supplementary Figure 85 ^{1}**H NMR**-spectrum (400 MHz, $CDCl_3$) of **44**

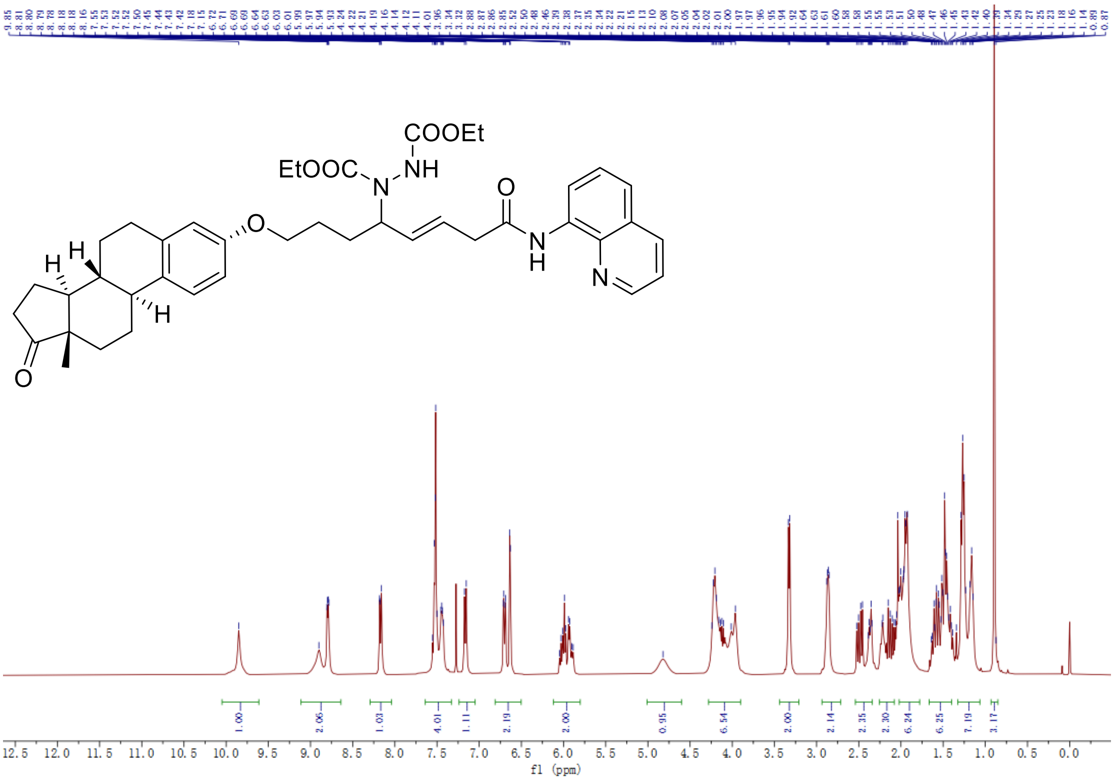

Supplementary Figure 86 **^{13}C NMR**-spectrum (101 MHz, $CDCl_3$) of **44**

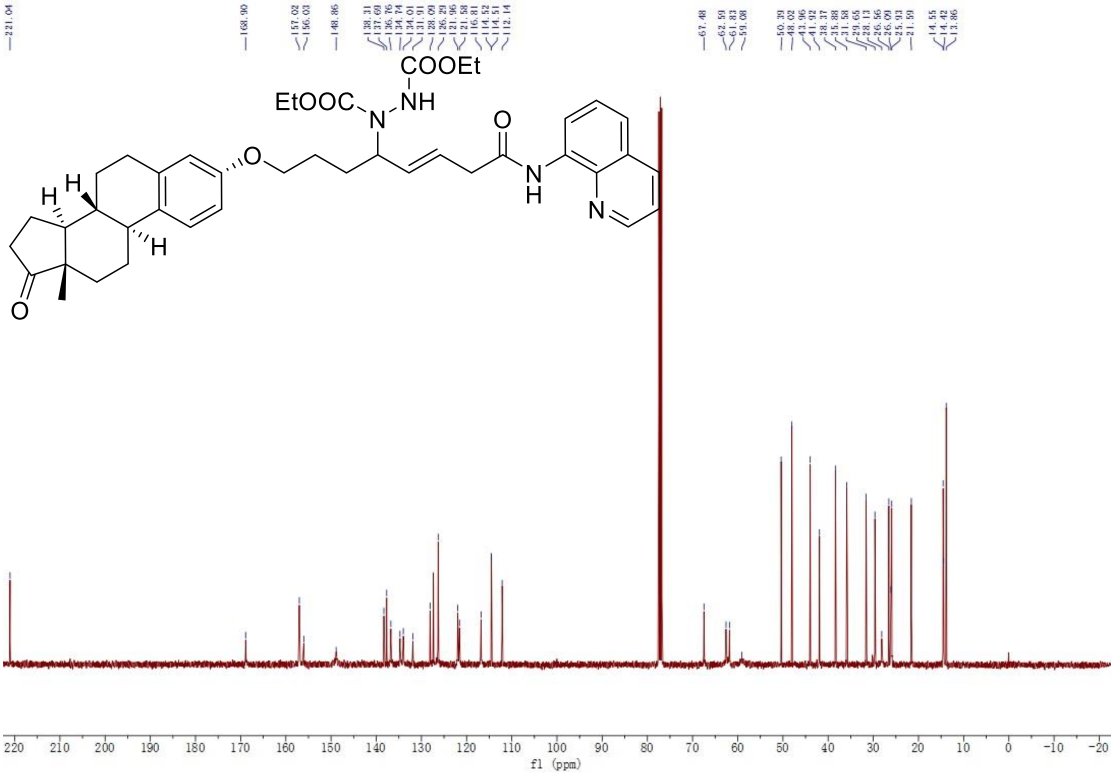

Supplementary Figure 87 **^{1}H NMR**-spectrum (400 MHz, $CDCl_3$) of **45**

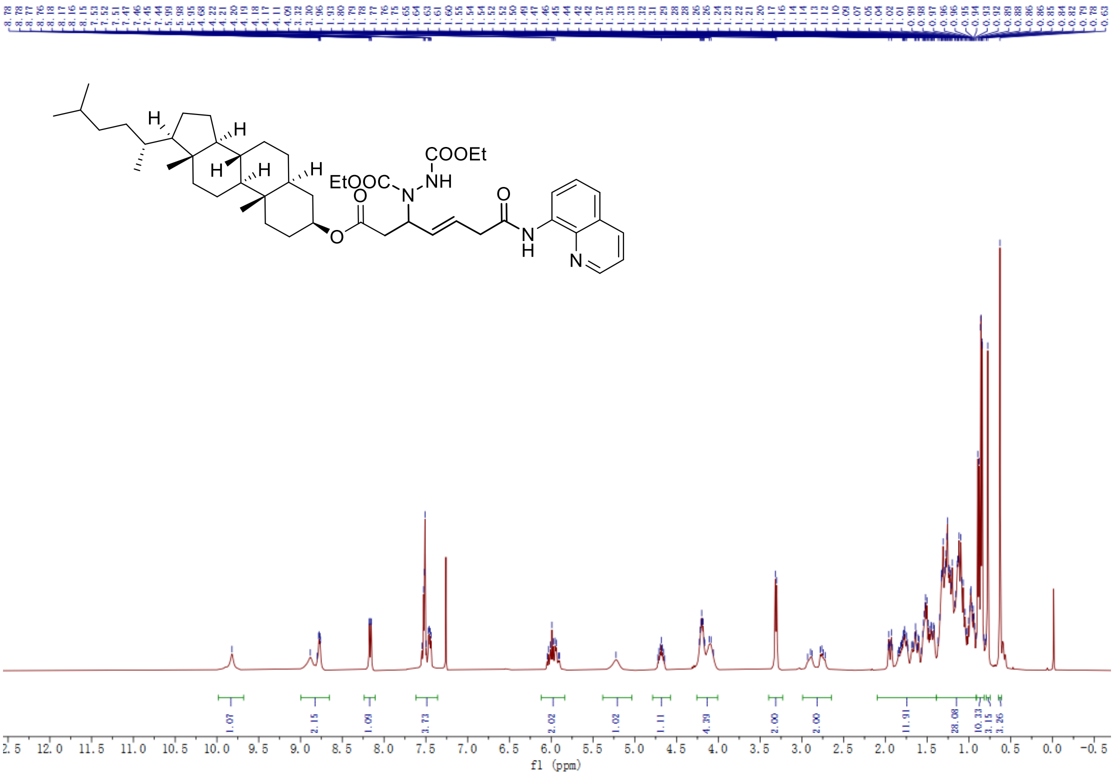

Supplementary Figure 88 ^{13}**C NMR**-spectrum (101 MHz, $CDCl_3$) of **45**

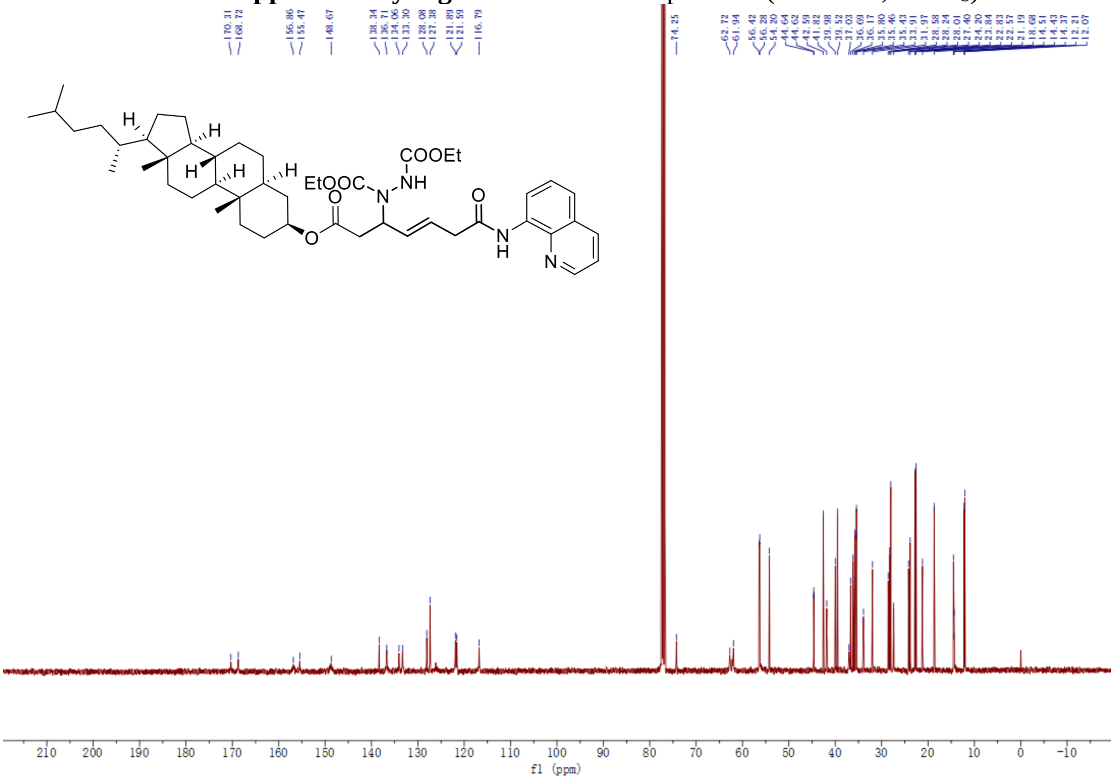

Supplementary Figure 89 ^{1}**H NMR**-spectrum (400 MHz, $CDCl_3$) of **46**

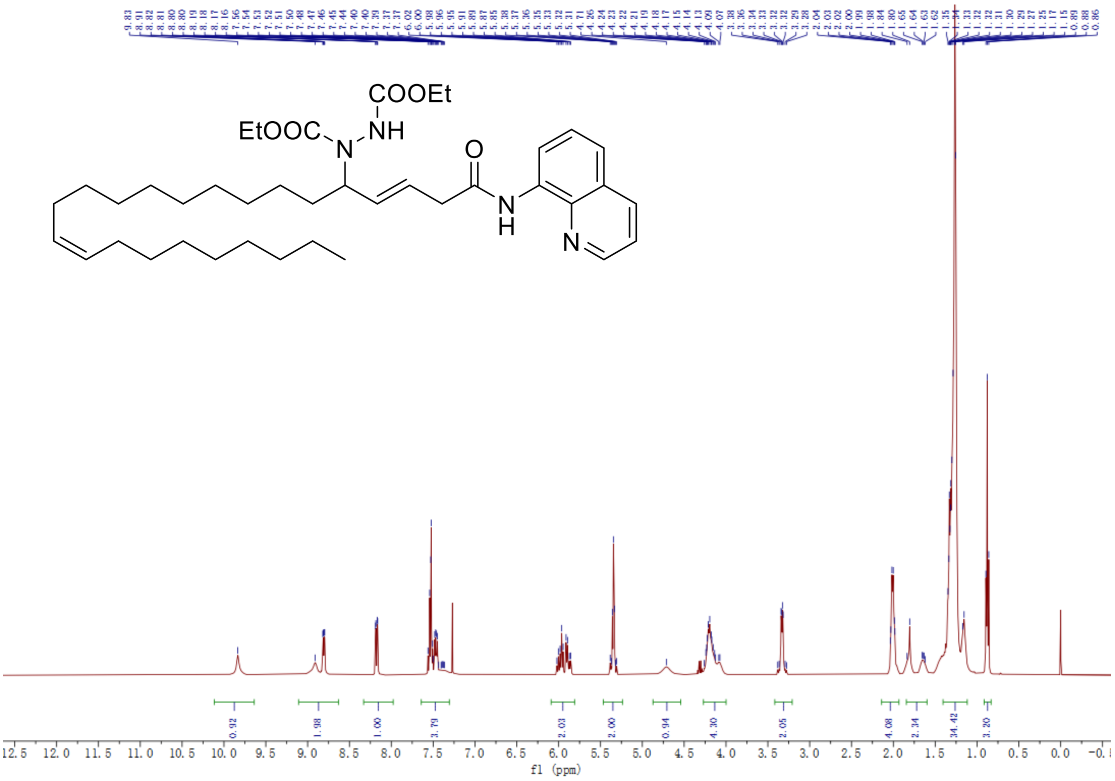

Supplementary Figure 90 ^{13}**C NMR**-spectrum (101 MHz, $CDCl_3$) of **46**

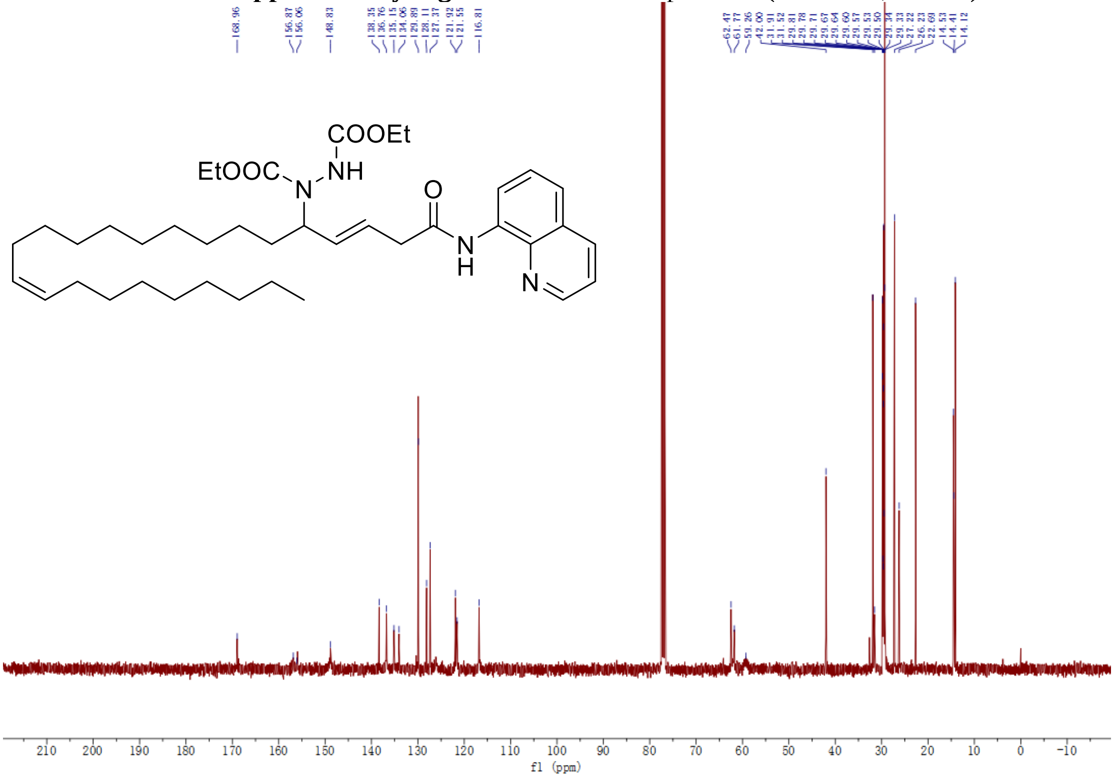

Supplementary Figure 91 ^{1}**H NMR**-spectrum (400 MHz, $CDCl_3$) of **47**

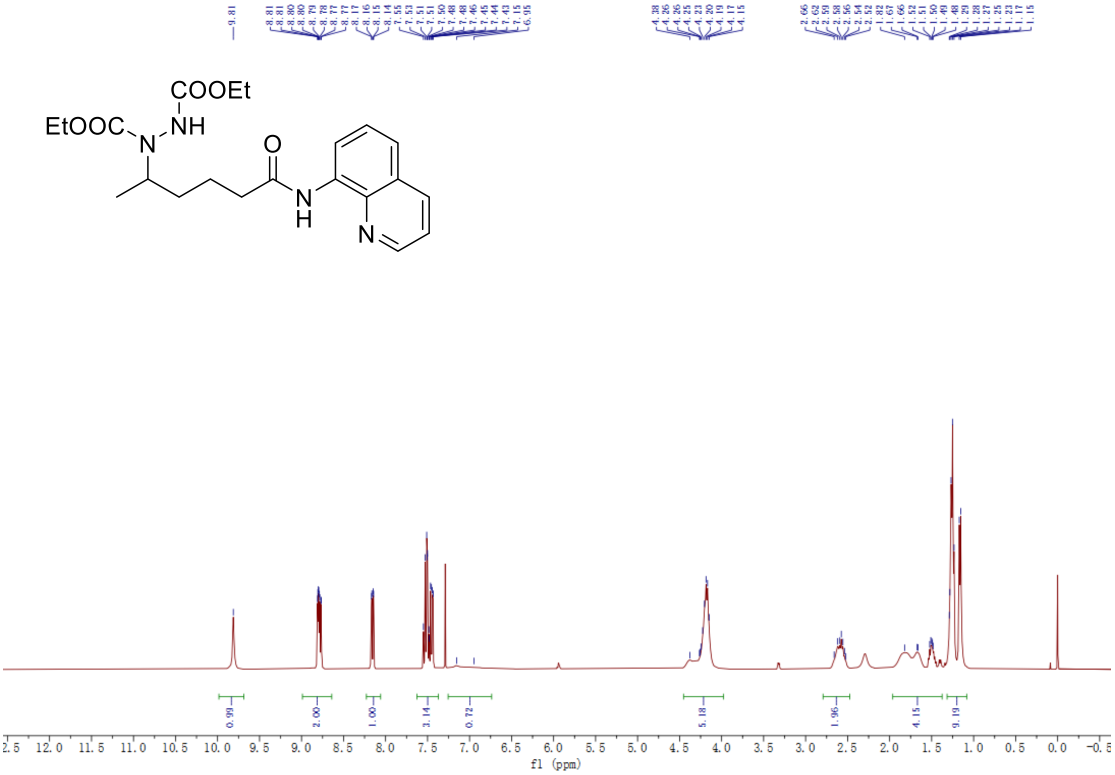

Supplementary Figure 92 ^{13}**C NMR**-spectrum (101 MHz, $CDCl_3$) of **47**

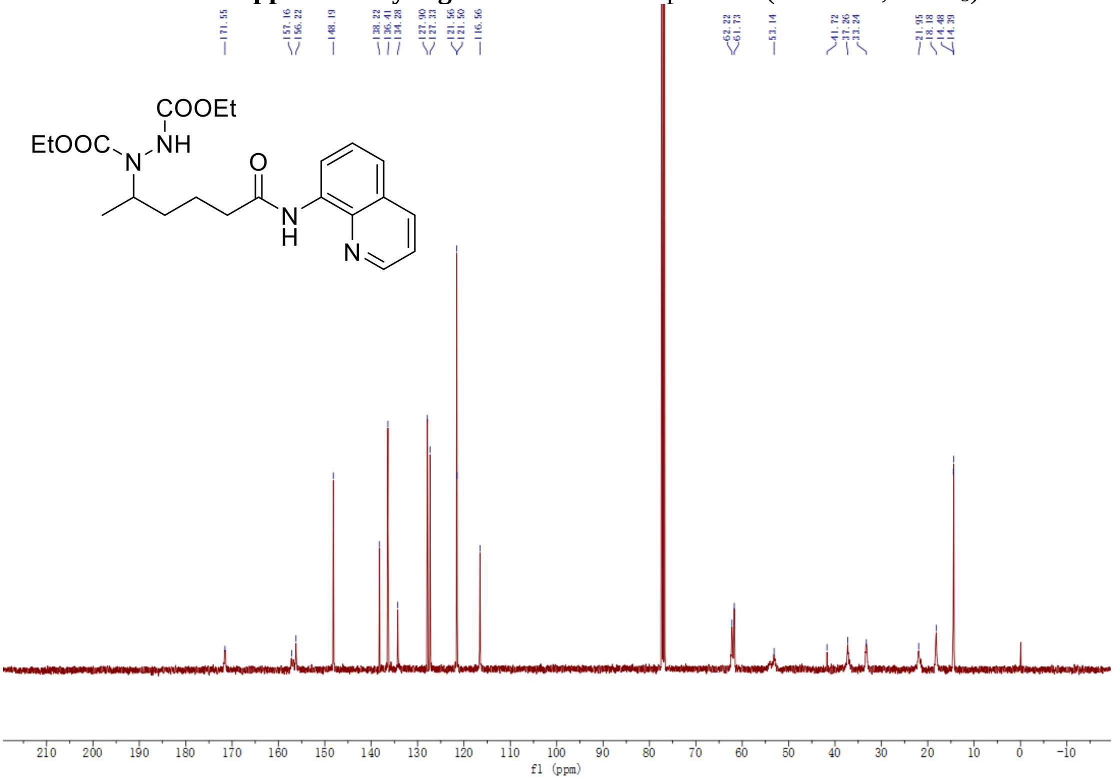

Supplementary Figure 93 ^{1}**H NMR**-spectrum (400 MHz, $CDCl_3$) of **48**

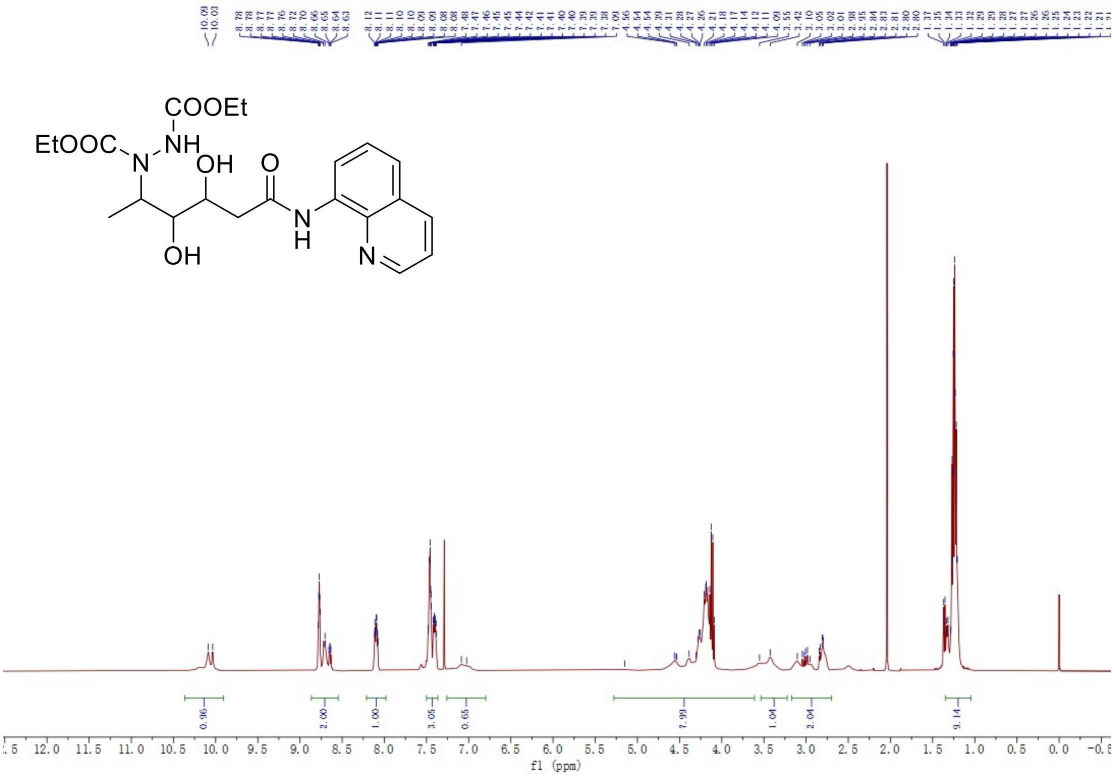

Supplementary Figure 94 **^{13}C NMR**-spectrum (101 MHz, $CDCl_3$) of **48**

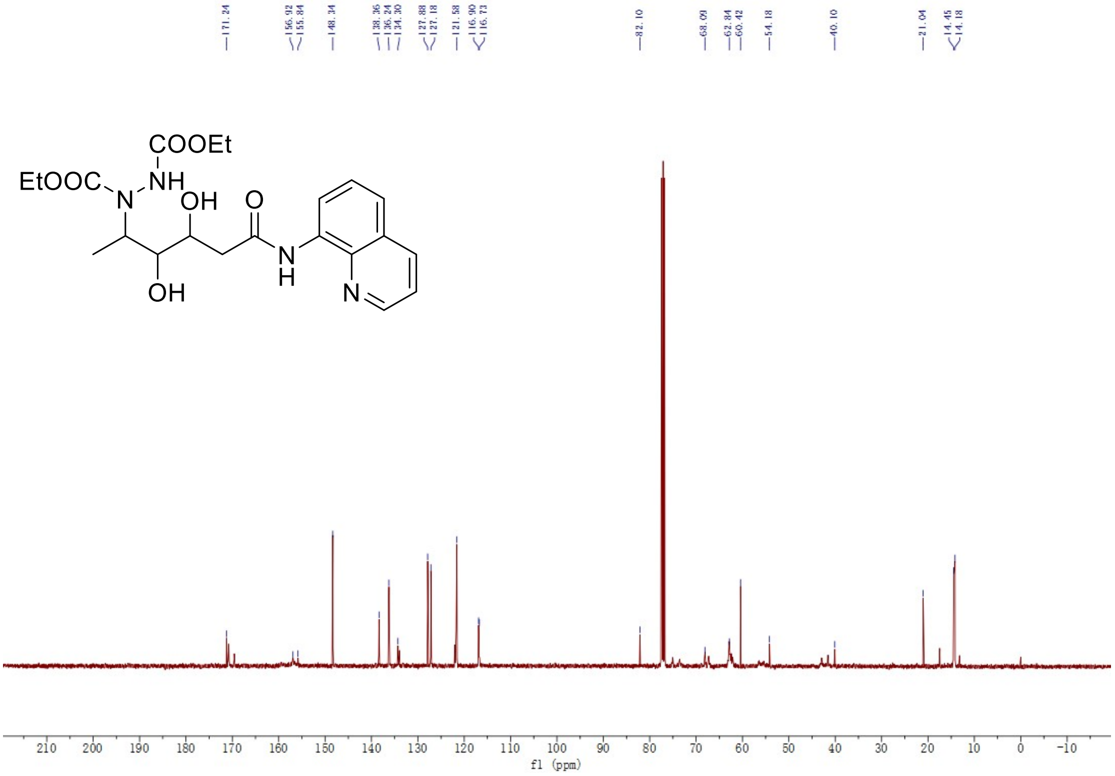

Supplementary Figure 95 **^{1}H NMR**-spectrum (400 MHz, $CDCl_3$) of **49**

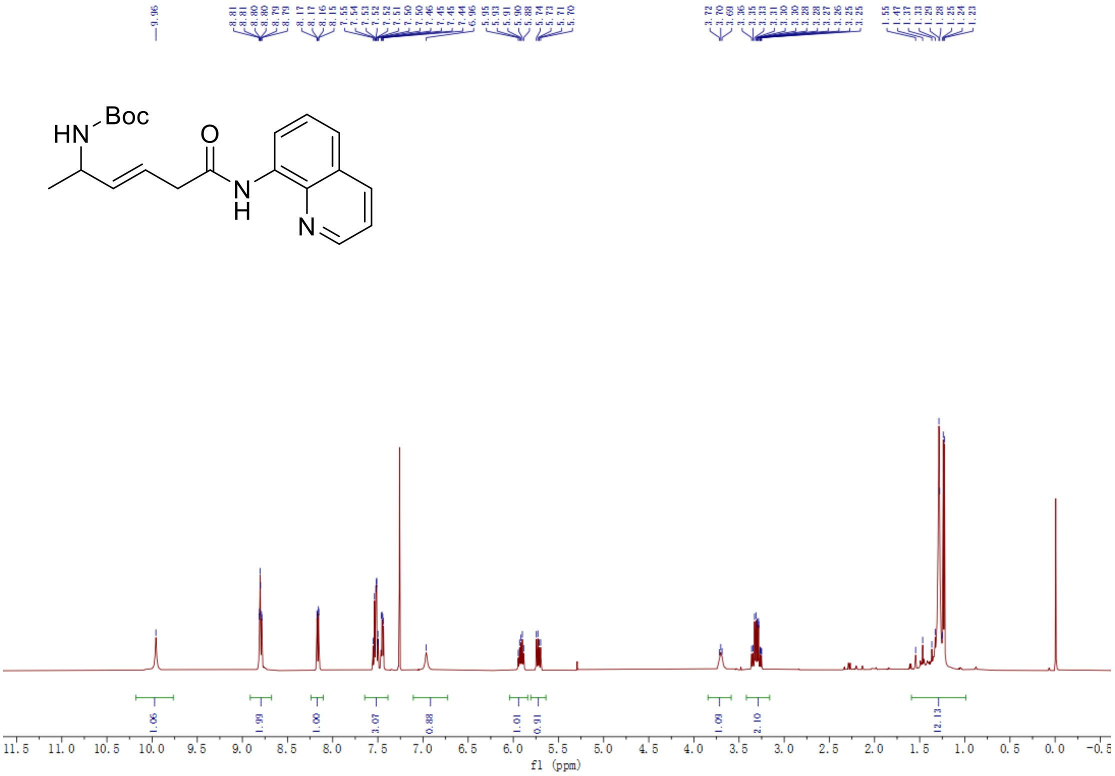

Supplementary Figure 96 ^{13}**C NMR**-spectrum (101 MHz, $CDCl_3$) of **49**

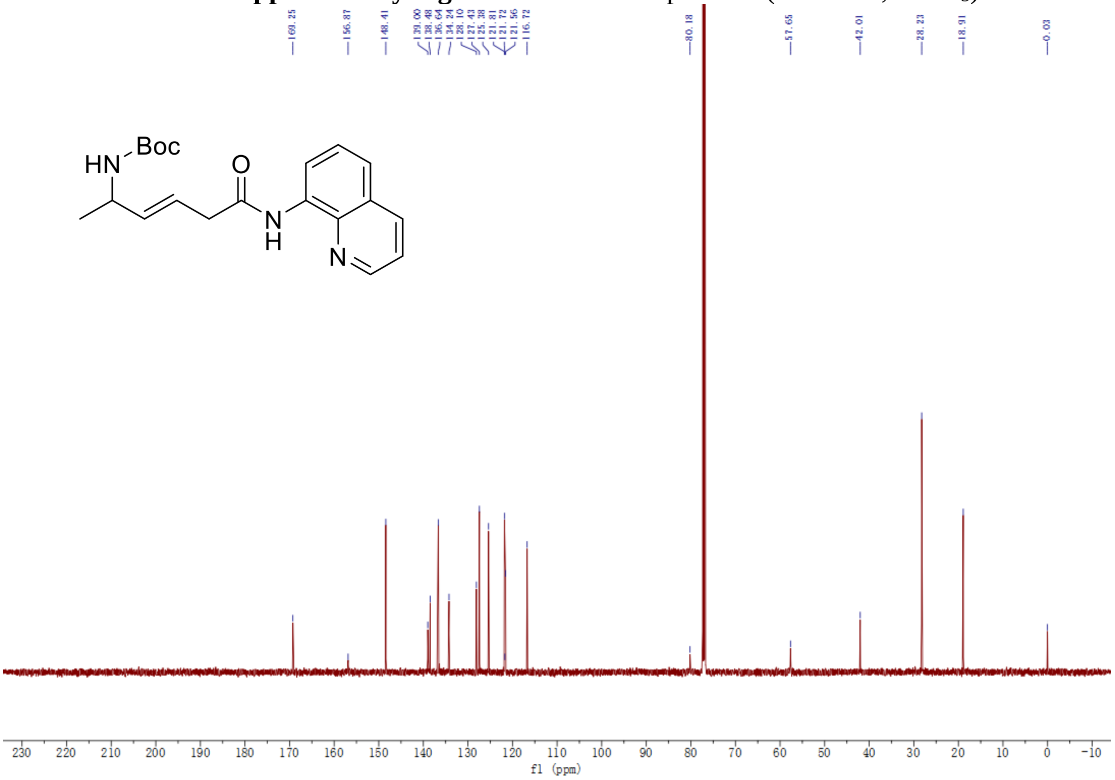

Supplementary Figure 97 ^{1}**H NMR**-spectrum (400 MHz, $CDCl_3$) of **50**

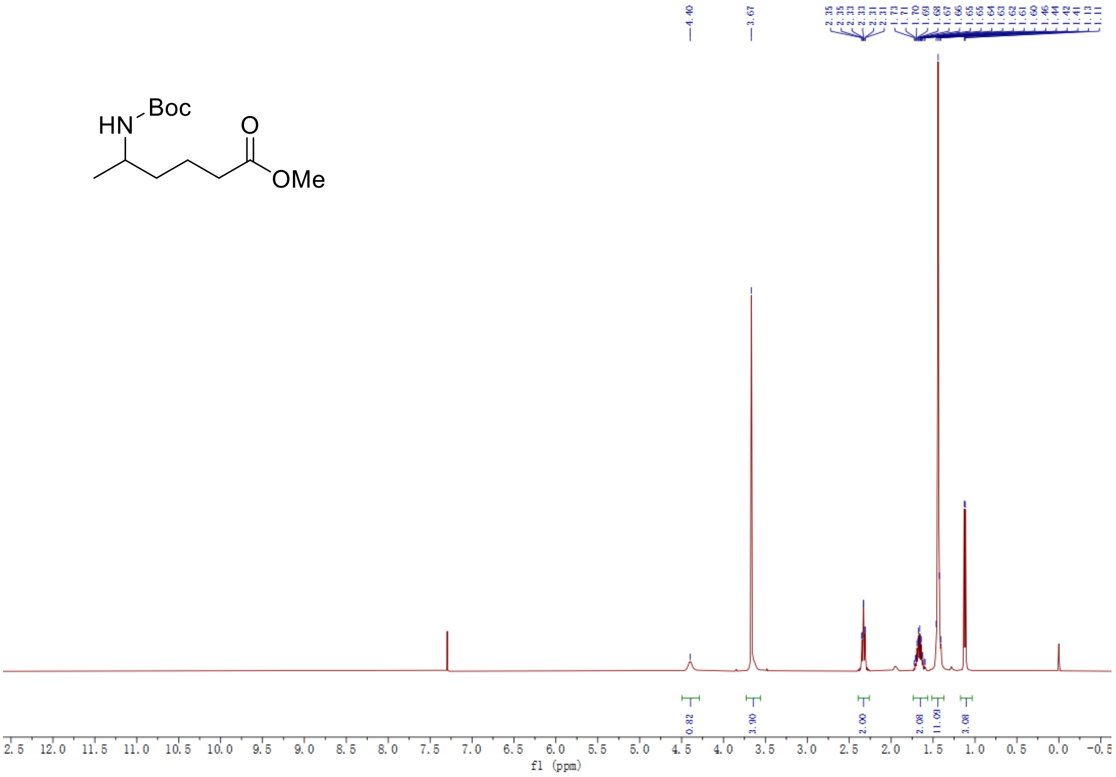

Supplementary Figure 98 ^{13}C NMR-spectrum (101 MHz, $CDCl_3$) of **50**

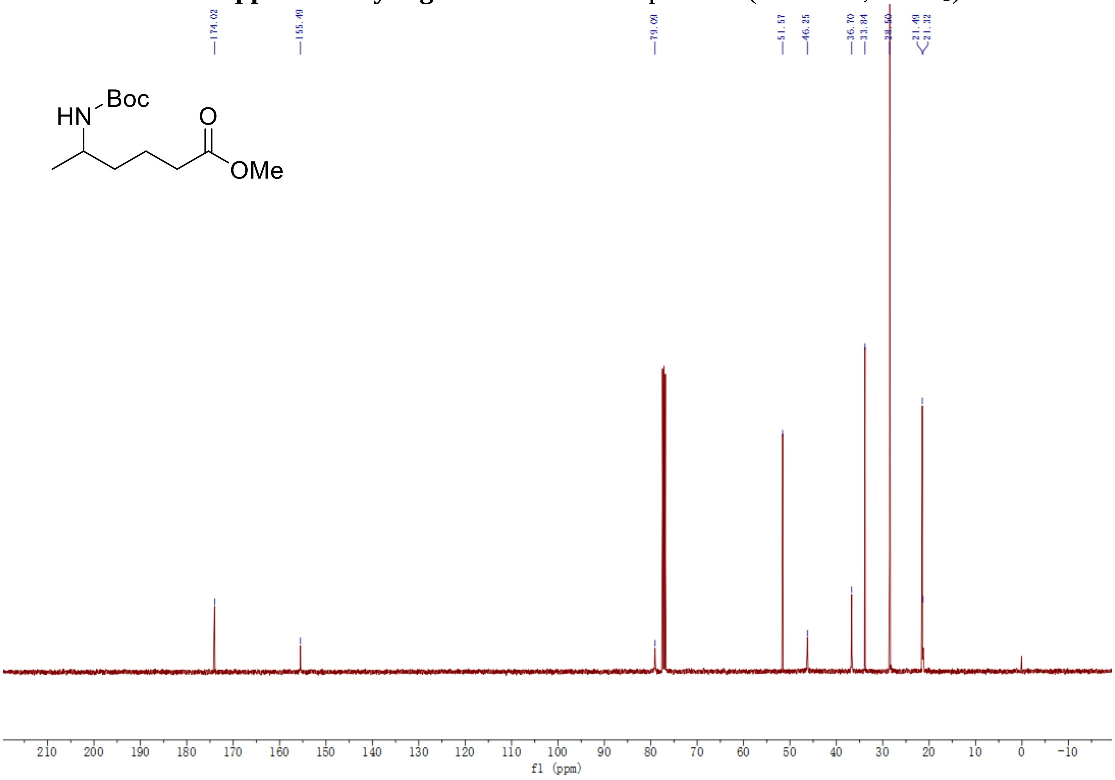

11. Supplementary References

1. Wang, H.; Bai, Z. B.; Jiao, T. Q.; Chen, G. Palladium-Catalyzed Amide-Directed Enantioselective Hydrocarbofunctionalization of Unactivated Alkenes Using a Chiral Monodentate Oxazoline Ligand. *J. Am. Chem. Soc.* **140,** 3542-3546 (2018).
2. Jeon, J.; Ryu, H.; Hong, S. Site-Selective 1,1-Difunctionalization of Unactivated Alkenes Enabled by Cationic Palladium Catalysis. *J. Am. Chem. Soc.* **141,** 10048-10059 (2019).
3. Kawamata, Y.; Hashimoto, T.; Maruoka, K. A Chiral Electrophilic Selenium Catalyst for Highly Enantioselective Oxidative Cyclization. *J. Am. Chem. Soc.* **138,** 5206-5209 (2016).
4. Zhao, Y.; Truhlar, D. G. The M06 suite of density functionals for main group thermochemistry, thermochemical kinetics, noncovalent interactions, excited states, and transition elements: two new functionals and systematic testing of four M06-class functionals and 12 other functionals. *Theor Chem Acc.* **120,** 215-241 (2008).
5. Hay, P. J.; Wadt, W. R. Ab Initio Effective Core Potentials for Molecular Calculations. Potentials for K to Au Including the outermost Core Orbitals. *J. Chem. Phys.* **82,** 299-310 (1985).
6. Roy, L. E.; Hay, P. J.; Martin, R. L. Revised Basis Sets for the LANL Effective Core Potentials. *J. Chem. Theory Comput*. **4,** 1029-1031 (2008).
7. Krishnan, R.; Binkley, J. S.; Seeger, R.; Pople, J. A. Self-consistent molecular orbital methods. XX. A basis set for correlated wave functions. *J. Chem. Phys.* **72,** 650-654 (1980).
8. McLean, A. D.; Chandler, G. S. Contracted Gaussian basis sets for molecular calculations. I. Second row atoms, Z=11–18. *J. Chem. Phys.* **72,** 5639-5648 (1980).
9. Marenich, A. V.; Cramer, C. J.; Truhlar, D. G. Universal Solvation Model Based on Solute Electron Density and on a Continuum Model of the Solvent Defined by the Bulk Dielectric Constant and Atomic Surface Tensions. *J. Phys. Chem. B.* **113,** 6378-6396 (2009).
10. Dolg, M.; Wedig, U.; Stoll, H.; Preuss, H. Energy-adjusted *ab* initio pseudopotentials for the first row transition elements. *J. Chem. Phys.* **86,** 866-872 (1987).
11. Andrae, D.; Häußermann, U.; Dolg, M.; Stoll, H.; Preuß, H. Energy-adjustedab initio pseudopotentials for the second and third row transition elements. *Theor. Chim. Acta*. **77,** 123-141 (1990).
12. Frisch, M. J.; Trucks, G. W.; Schlegel, H. B.; Scuseria, G. E.; Robb, M. A.; Cheeseman, J. R.; Scalmani, G.; Barone, V.; Mennucci, B.; Petersson, G. A.; Nakatsuji, H.; Caricato, M.; Li, X.; Hratchian, H. P.; Izmaylov, A. F.; Bloino, J.; Zheng, G.; Sonnenberg, J. L.; Hada, M.; Ehara, M.; Toyota, K.; Fukuda, R.; Hasegawa, J.; Ishida, M.; Nakajima, T.; Honda, Y.; Kitao, O.; Nakai, H.; Vreven, T.; Montgomery, J. A.; Peralta, J. E.; Ogliaro, F.; Bearpark, M.; Heyd, J. J.; Brothers, E.; Kudin, K. N.; Staroverov, V. N.; Keith, T.; Kobayashi, R.; Normand, J.; Raghavachari, K.; Rendell, A.; Burant, J. C.; Iyengar, S. S.; Tomasi, J.; Cossi, M.; Rega, N.; Millam, N. J.; Klene, M.; Knox, J. E.; Cross, J. B.; Bakken, V.; Adamo, C.; Jaramillo, J.; Gomperts, R.; Stratmann, R. E.; Yazyev,532 O.; Austin, A. J.; Cammi, R.; Pomelli, C.; Ochterski, J. W.; Martin, R. L.; Morokuma, K.; Zakrzewski, V. G.; Voth, G. A.; Salvador, P.; Dannenberg, J. J.; Dapprich, S.; Daniels, A. D.; Farkas, O.; Foresman, J. B.; Ortiz, J. V.; Cioslowski, J.; Fox, D. J.; Gaussian 09, Revision D.01, Gaussian, Inc., Wallingford CT, (2013).
